# coPLINK: A complementary tool to PLINK

**DOI:** 10.1371/journal.pone.0239144

**Published:** 2020-09-18

**Authors:** Han-Ming Liu, Zhao-Fa Liu, Jin-Ping Zheng, Dan Yang, Sheng-Zhou Hu, Shen-Hai Yan, Xian-Wen He

**Affiliations:** School of Mathematics and Computer Science, Gannan Normal University, Ganzhou, Jiangxi, China; University of North Carolina at Chapel Hill, UNITED STATES

## Abstract

In genome-wide association studies (GWAS), a wide variety of analysis tools have been designed, leading to various formats of GWAS data. How to convert a dataset in non-PLINK format into PLINK format to use its powerful analysis performance, or to convert a dataset in PLINK format into the format of other analysis tools, is a problem that needs to be faced and solved. To address this issue, we developed a tool called coPLINK, a complementary tool to PLINK, to cooperate with PLINK to implement the conversions of GWAS data formats and to provide some additional functions, such as data files comparison. The tool can implement mutual conversions not only between an existing data format and PLINK PED/BED, but also between a user-defined data format and PLINK PED. The usage and performance of the tool are similar to PLINK. The characteristics of the conversions of existing data formats and user-defined formats make it be a good assistant to PLINK or other tools and, have good potential for GWAS studies or other works.

## Introduction

Since the birth of the landmark [1] in 2005, a genome-wide association study (GWAS) has quickly become the focus of bioinformatics. GWAS has a wide range of uses in whether it is a complex disease [2] or a genetic breeding [3, 4]. To help researchers deal with GWAS-related works handily, Shaun Purcell et al. developed a whole genome association analysis toolset called PLINK to perform a range of basic, large-scale analyses [5, 6]. PLINK is a free, open source tool that has support for subsequent visualization, annotation, and results storage through integration with gPLINK and Haploview. Due to its powerful functions, it is widely used in bioinformatics. So far, the first generation of PLINK [5] has been cited as many as 13,949 times (Web of Science, 1,073 times per year), while the times cited count of the second generation [6] has reached 1,500 (Web of Science, 300 times per year).

PLINK mainly supports self-defined or some general formats, such as.ped,.bed and.dat. To exchange data between PLINK and other GWAS tools, we developed a complementary tool to PLINK, coPLINK. coPLINK is a cooperator of PLINK that mainly used to implement mutual conversions between non-PLINK data formats and the main formats of PLINK. The non-PLINK format can be any format, even a user-defined format. And, it can fulfill some tests and/or data preprocessings during the conversion, including Hardy-Weinberg equilibrium test, alleles missing rates test of samples and/or SNPs, normalizing and swapping. In addition to format conversions, we added some potentially useful functions to the tool, such as data rows and/or columns inserting, data rows and/or columns deleting and data file contents comparison. The tool supports 45 main functions distinguished by a term "parameter" (see Table 1 in S1 File), and each function can be divided into several sub-functions, a total of 192.

## Materials and methods

### Genotype data

The genotype data was used to test the software, it comes from the data of coronary artery disease (CAD) of the Wellcome Trust Case Control Consortium (WTCCC)[7], which contains 23 sets of autosome 1–22 and sex chromosome X. The datasets include 490,032 SNPs and 3,488 samples (1,988 cases and 1,500 controls), occupying ~4 GB storage. Since the format of the datasets is TPED containing.tped,.tfam and.tsnp files, and the case and control data are separately stored in two groups of files, we converted firstly the two groups of files into PED files and then spliced them into one group, to facilitate the subsequent test experiments and to make one group of data have two phenotypes. The WTCCC data were used here because they contain the optional.tsnp files can easily check the feasibility of the tool converting TPED with.tsnp to PED.

### Software testing

The testing includes a functional test and a performance test. A method of black-box testing was employed to test the software functions, and two generations of PLINK were used as the benchmarks to test whether the software performance has negative effect on PLINK. A black-box testing is executed without having an insight into the details of the underlying code and performed solely by executing the compiled code.

## Results

### Existing format conversion

coPLINK provides 39 format conversions (see S1 Table in S1 File), including the mutual conversions of PLINK PED/BED and 24 other data formats such as BEAM[8], BOOST[9] and MDR[10], as well as the mutual conversion between space-delimited text and csv format (Table 1). Moreover, each conversion has 1–7 options called "indicators" (see S2 Table in S1 File) to implement different sub-functions. These conversions make it possible to exchange data between PLINK and other GWAS analysis tools. Of course, using PLINK PED (or BED) format as an intermediate, we can easily implement a conversion of non-PLINK formats. Besides the conversions between PLINK PED/BED and other formats, the tool also provides the conversions of PLINK PED, BED and TPED formats to avoid changing the order of SNPs. A fixed order of SNPs may help to manually check and/or compare data and thus bringing a lot of convenience in certain applications such as a GWAS algorithm design. In addition, when converting TPED to PED (or BED), this tool will extract RS# from the optional.tsnp file.

**Table 1:** Supported formats by coPLINK.

Format	Supported by PLINK
BEAM	N
BOOST	N
ME	N
GEO	N
GS-linkage	N
Linkage	N
LogicReg^#^	N
MDR	N
ME	N
PrettyBase^##^	N
SVMSNPs^###^	N
User-defined TPED	N
CSV	N
Space-delimited	N
User-defined	N
BED	Y
PED	Y
TPED	Partly

^#^ 6 combinations of genetic models and phenotypes (Logited and non-logited).

^##^ includes Slider and SeattleSNPs versions.

^###^ includes letter order and major counting formats.

### Inserting, deleting and transposing

The software can easily insert or delete rows and/or columns in a plain-text file, and the number of rows (columns) inserted or deleted is unlimited. Furthermore, a transpose function was added to exchange the rows and columns of a text file.

### User-defined format conversion

Since the data format cannot be constant, new formats of GWAS data will be generated continuously. In order to deal with the format changes, we designed a function to convert mutually between the PLINK PED and a user-defined (or tool-defined) format. This function gives the user an opportunity to convert one or more files without limited formats to a group of PED files, and vice versa. Moreover, similar to the conversion of existing formats, we can use the PED format as the intermediate plus the functions of inserting, deleting and/or transposing (although not required in most cases) to convert data between any formats.

Some regular expressions can be used to represent the column and/or row names of a new format. The tool provides four wildcards "#", "$", "@", "^" and their escape characters to generate regular items (Table 2).

**Table 2:** Wildcards and escape characters.

Wildcard	Function	Escape char.^#^	Example
#	serial numbers of the allele columns/rows	\#	A# = A1, A2, …
$	serial numbers of the SNP columns/rows	\$	SNP$ = SNP1, SNP2, …
@	serial numbers of the sample columns/rows	\@	SAM@ = SAM1, SAM2, …
^	two alternate characters	\^	A^ab = Aa, Ab, Aa, …

^#^ In addition to these escape characters, the tool supports ‘\,’ and ‘\t’ for a comma and a tab character, respectively.

**Fig 1:**
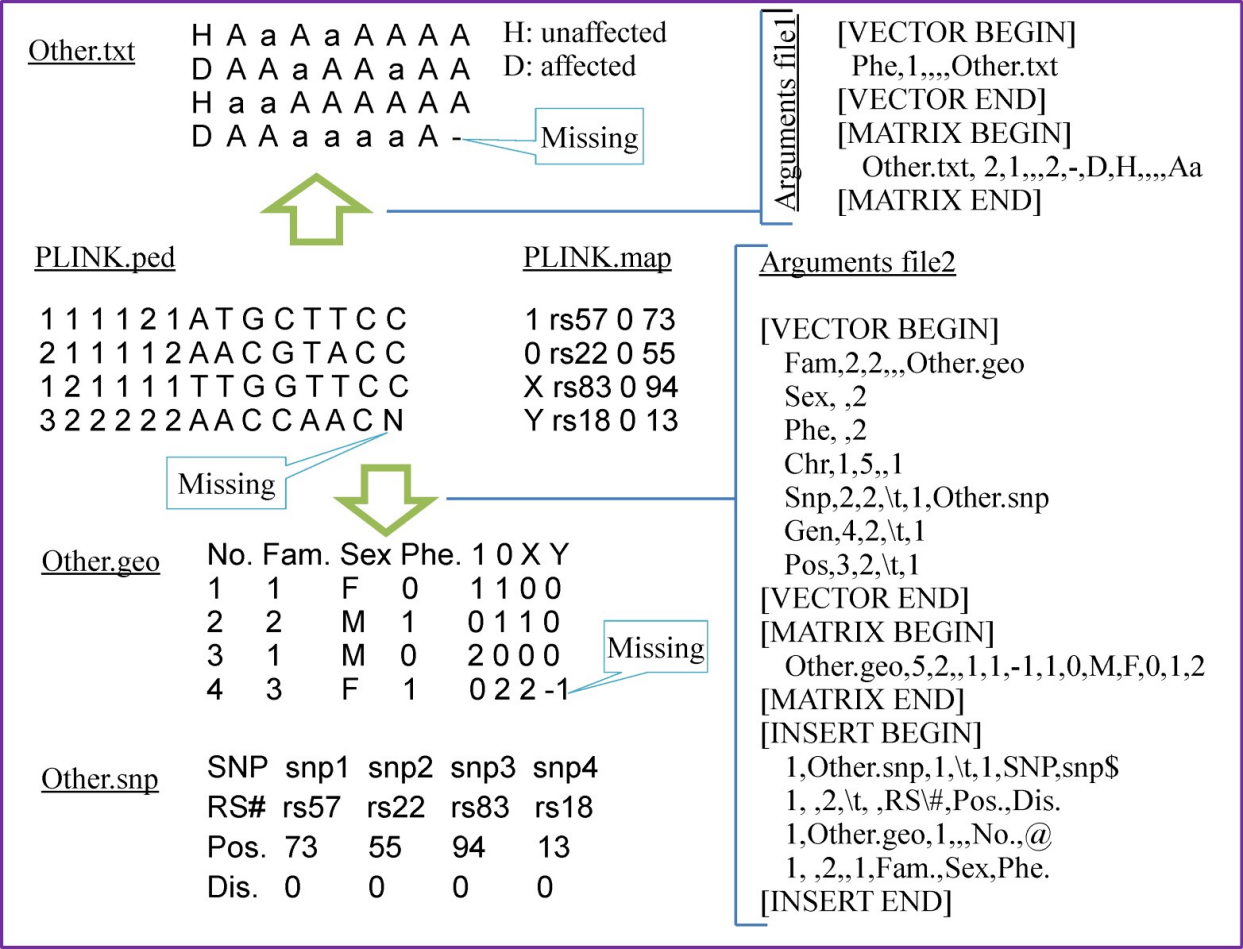
Example of converting PLINK PED to user-defined format. The files Other.geo and Other.txt are space delimited (more spaces were added for readability), and the Other.snp is tab delimited. The delimiter in the arguments files is a comma which can be ignored if there are no more items such as “Sex, 2” in Arguments file 2. The MATRIX section describes the components of the genotype matrix while the VECTOR section gives the other components of the data, and the INSERT section provides how to insert additional rows/columns.

Since the "user-defined format" is unknown to coPLINK, it needs a specification called "arguments file" to explain the format. As an example, Fig 1 shows the elements of an arguments file (see section "22—other2ped" and section "32—ped2other" of S1 File for details). Although the PLINK PED file illustrated in Fig 1 is a small-scale dataset containing only four samples and four SNPs, the arguments file can be applied to data of any size. Moreover, an arguments file can be usually used to convert mutually between PED and other format.

### File comparison

The file comparison of operating systems is usually a simple sequence analysis of equality. This tool can compare two plain-text or binary files by not only sequence but also a reference column (or field), and record in detail the rows and columns where the differences are. In addition, when the delimiter is a space (or tab), it will replace two (or more) delimiters with one and remove the leading and trailing spaces (or tabs) while comparing. Here, the reference-based comparison is an interesting feature in GWAS data analysis. For example, if the two files shown in Table 3 are compared sequentially, there are five different rows. However, when the second column is specified as the reference, coPLINK can easily find that only one row is different and the difference from the two files is in column 4.

**Table 3:** Two sample files.

Ex1.dat	Ex2.dat
0 SNP4 0 0	0 SNP1 0 0
0 SNP3 0 0	↲
↲^#^	0 SNP2 0 ***0***
0 SNP2 0 ***1***	0 SNP3 0 0
0 SNP1 0 0	0 SNP4 0 0

^#^'↲' means a null row.

### File splicing

PLINK can merge the genotypes of two (or more) PED files, that is, SNPs combination. The file splicing here is not to merge SNPs but to merge the samples of two PED files, namely samples combination. For example, for two files with four and six samples, coPLINK will produce the result of 10 samples after splicing. During the splicing, the tool will automatically align the SNPs of the two files based on the SNP order of the first file, to avoid failure or throwing an exception caused by the different SNP orders. Furthermore, if some SNPs or genotypes in one file do not exist in the other, those SNPs or genotypes in the other file will be marked as missing. Similarly, if the two files have the same sample, the spliced result will only save the sample of the first file to avoid duplication. Samples with the same first six columns are considered as the “same” sample, regardless of the genotype columns. This function allows splicing two files only, and we can call the function repeatedly to splice more than two files.

### Normalizing and swapping

Normalizing refers to the normalization of genotypes, which (1) uses letters "D", "d" and "N" to represent the major, minor and missing allele respectively which are the same as PLINK, (2) uses a space as the column delimiters and (3) treats the first character of a multi-character allele as the allele character. The normalizing helps to intuitively understand the composition of the major and minor alleles of each SNP when checking data manually.

The sub-function "swapping" swaps the characters of major and minor alleles when a SNP has the same number of major and minor alleles. For example, after swapping, the genotype sequence "AA AC CC" of a SNP will change into "CC CA AA". In fact, the nature of a SNP is the same in this case whether they are swapped or not. This sub-function is mainly used for data analysis, such as data comparison.

### Functional test

During the software development phase, we designed test cases to test its functions. And, in S1 File, the examples of each function can also be regarded as part of the functional test. The input data of test cases used in development phase were artificially designed, however, it is very important to employ real data for further functional test of software to ensure its correctness and robustness. The functional test cases and test results based on the spliced CAD data are shown in S1 Table in S2 File, and the test script is shown in S1 List in S2 File. S1 Table shows all functional tests passed.

### Performance test

In the performance test, we employed PLINK 1.07 (the first generation) and 1.9 (the second generation) as the benchmarks, and tested two important items of elapsed time and peak memory. The test used the function of PED to BED format that all the three testees have, to continuously convert the 23 chromosomes of the spliced CAD data. The test results are shown in Table 4.

**Table 4:** Average elapsed time and peak memory^#^.

Tool	Elapse (s)	Peak memory (KB)	Ratio^##^
coPLINK	37.3	154,604	0.516
PLINK 1.07	62.0	33,654	0.102
PLINK 1.9	9.3	21,708	0.0766

^#^Environment: Windows 7 x86 OS, Intel i3 CPU @ 3.7GHz, integrated video adapter.

^##^Ratio = Peak memory/File size

Table 4 shows that the elapsed time of coPLINK is between those of the first and the second generation of PLINK. Although coPLINK is not as fast as the second generation of PLINK, it seldom involves frequent format conversion in actual applications, and thus, as an assistant of PLINK, it hardly has a negative effect on PLINK, that is, its speed can still meet the application requirements. Moreover, the table also shows that the peak memory of coPLINK is much higher than those of the two generations of PLINK. It is because the software needs normalizing, swapping and other operations while converting so that the entire source files need to be loaded into the memory at the same time. However, from the average ratio of peak memory to file size (about 0.5), it is not difficult to implement the conversion of GB-level big data to modern computer systems. For example, on a Windows 64 bit computer with 16 GB memory, we successfully tested coPLINK to convert a big simulation data from TPED to PED format by consuming 15.2 GB physical memory and 92.7 GB virtual memory. The data includes 4 million SNPs and 3,000 samples, occupying 44.8 GB of storage space. The reason for converting TPED to PED format is that the conversion is the maximum ratio of peak memory to file size in all text format conversions (see S2 Table in S2 File). Although S2 Table shows the maximum ratio is the conversion—transpose (mode 0) of 10.050, it will automatically switch to mode 1 to reduce memory requirement for a big dataset.

**Fig 2:**
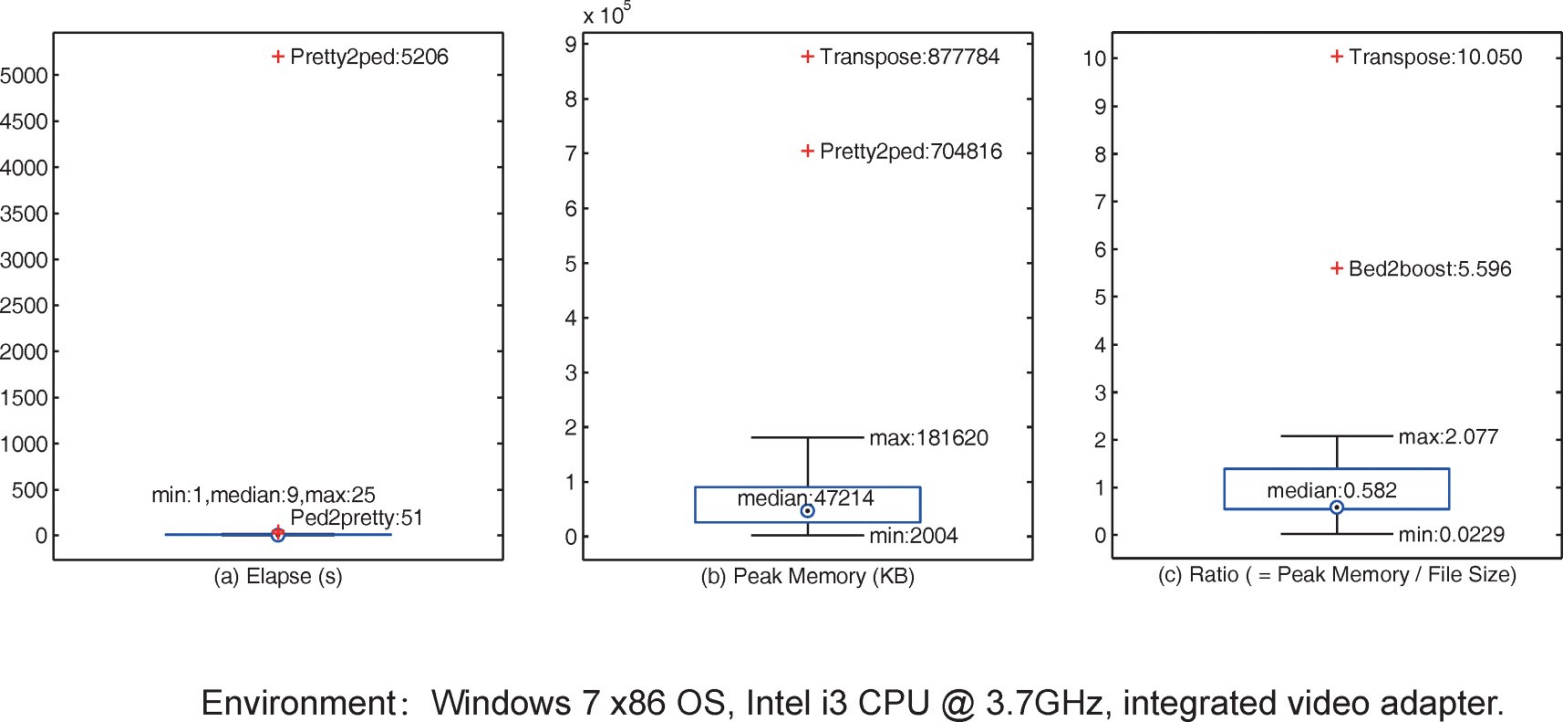
Elapsed time and peak memory of coPLINK.

In addition, we also employed the data of chromosome 19 as the input to test the elapsed time and peak memory for each conversion of this tool (Fig 2). The detailed elapsed time and peak memory usages are shown in S2 Table in S2 File, as well as the test script is shown in S2 List in S2 File.

A PrettyBase format (http://pga.mbt.washington.edu) file stores a lot of redundant information, its size is usually more than 6.5 times the size of a PED file, and thus it is necessary to consume a lot of computational resources to load and analyze the file. As shown in Fig 2(A) and 2(B), the elapsed time and peak memory usage of converting PrettyBase format to PED are significantly higher than the median. In addition to this conversion, Fig 2(B) and 2(C) show that the transpose function also consumes a considerable amount of memory which is 10 times the size of the source file. The reason is that PED format treats each allele of a SNP as a column resulting in a large number of columns in the dataset, and the memory consumption of the function depends heavily on the number of rows and columns in the source file. To transpose a big file with a large number of rows and columns, coPLINK provides a memory-first mode (lower memory consumption) in which the peak memory is reduced to 1.029 times the size of the source file (S2 Table in S2 File). Moreover, based on the available memory of the computer, coPLINK can automatically select the appropriate operating mode—speed-first mode (needs more memory) or memory-first mode to transpose a file. It is also worth noting that Fig 2(C) shows that when converting PLINK BED format to BOOST, the ratio of peak memory to file size is as high as 5.596. Fortunately, since the size of a BED format file is much smaller than that of the PED format (the former is about one-sixteenth of the latter), the peak memory of this function usually does not exceed the available memory and has no a negative impact. Fig 2(B) shows that the peak memory of this function does not significantly exceed the median approves this view.

## Discussion

As a complementary to PLINK, coPLINK enhances the functions of data format conversion for PLINK. It has the following distinctive features:

- It implements the conversions not only of the existing formats but also of user-defined formats.
- It provides some additional functions, such as file comparison and row/columns insertion and deletion.
- For the mutual conversion between PED and other formats and the inserting function, when the specified arguments file does not exist, the program will automatically generate a template of the arguments file and the template illustrates the structure and meaning of each argument in detail.
- It provides some automation, such as automatic version identification of PrettyBase format and automatic selection of operating mode of transposing.
- It provides dynamic and complete information prompts and a detailed log file in operation, which helps to track the running process of the program.

The above characteristics make the tool have good potential for GWAS data analysis and other works.

For the purpose of using—swap or other handles, coPLINK needs to read the entire files into memory, which results in it requiring more memory than the PLINKs (Table 4). coPLINK will use the virtual memory to simulate part of the physical memory when the physical memory is not enough to implement a conversion. As a way to avoid using virtual memory, we can (1) firstly, split a big data into smaller subsets through PLINK, (2) secondly, convert the subsets by coPLINK and (3) finally, merge/splice the results by PLINK/coPLINK. We will improve the function overloadings in the next generation to use memory dynamically based on handles, that is, coPLINK will load part (streamly, parallelly or part by part) or the entire files into the memory according to additional handles and the available memory to reduce the requirement of physical memory and avoid the usage of virtual memory.

## Supporting information

S1 File The manual of this tool. (PDF)

S2 File Detailed descriptions of the functional test and performance test. (DOCX)
