# Supplementary material for: coPLINK: A complementary tool to PLINK
Source: PLoS One. 2020 Sep 18;15(9):e0239144. doi: 10.1371/journal.pone.0239144 (PMC7500662; doi:10.1371/journal.pone.0239144)
Supplement: S1 File — (PDF) [file pone.0239144.s001.pdf]

coPLINK (V4) Manual

Bug-report: lhmgzjx@163.com

Feburay, 2020

Content

Part I Summary....4
1 Usage....4
2 Lists of Parameters and Indicators....7
Table 1. Parameters....7
Table 2. Indicators....11
Part II Specifications....12
1 --beam2ped....12
2 --beam2boost....16
3 --bed2beam....17
4 --bed2boost....19
5 --bed2me....20
6 --bed2tped....20
7 --boost2bed....21
8 --boost2ped....24
9 --compare....24
10 --csv2space....30
11 --delete....30
12 --geo2ped....33
13 --gs-linkage2ped....35
14 --insert....37
15 --linkage2boost....41
16 --linkage2ped....43
17 --logicreg2ped....45
18 --mdr2boost....48
19 --mdr2ped....50
20 --me2boost....51
21 --me2ped....53
22 --other2ped....54
23 --ped2beam....61
24 --ped2bed....63
25 --ped2boost....65
26 --ped2geo....66
27 --ped2gs-linkage....68
28 --ped2linkage....69
29 --ped2logicreg....70
30 --ped2mdr....72
31 --ped2me....73
32 --ped2other....74
33 --ped2ped....83
34 --ped2pretty....85
35 --ped2svmsnp....88
36 --ped2tped....91

37 --pretty2ped 93
38 --space2csv 97
39 --splice-ped 98
40 --svmsnp2ped 101
41 --test-allele 103
42 --tped2bed 105
43 --tped2ped 108
44 --tped2usr-tped 110
45 --transpose 112
History 116

Part I Summary

1 Usage

Basic format of coPLINK's command line:

coPLINK <parameter> <in> [[indicator1] [value1]] [[indicator2] [value2]] …

- coPLINK: required. Program name.
- parameter: required. Item of main function.
- in: required. Input filename (with a path if needed) or folder. If <in> contains spaces, enclose it in double quotes.
- indicator1: optional. Item 1 of sub-function 1.
- value1: optional. Value of indicator 1.

Cautions：

1. The help information for using coPLINK will be displayed on the screen while running the program without a parameter or with improper parameter or indicators.

```
#--------------------------------------------------------#
|          coPLINK          |       V4.0 Feb. 2020       |
|--------------------------------------------------------|
|  (C) 2020 Han-Ming LIU, GNU General Public License, V3  |
|--------------------------------------------------------|
|              Bug-report: lhmgzjx@163.com               |
|--------------------------------------------------------|
| Please cite the paper if you used this program in your |
| research for publication.                              |
#--------------------------------------------------------#
```

Invalid parameter!
Usage: coPLINK < parameter> <in> [--out <outputFilename>] [[indicator1] [value1]] [[indicator2] [value2]] …
The valid parameters and indicators are:
*Parameters:
--beam2boost: convert BEAM to BOOST
--beam2ped: convert BEAM to Plink ped
--bed2beam: convert Plink bed to BEAM

--bed2boost: convert Plink bed to BOOST

--bed2me: convert Plink bed to ME

--bed2tped: convert Plink bed to tped

--boost2bed: convert BOOST to Plink bed

--boost2ped: convert BOOST to Plink ped

--compare: compare two files

--csv2space: convert CSV to file with space delimiter

--delete: delete columns/rows from a plain-text file

--geo2ped: convert geo to Plink ped

--gs-linkage2ped: convert GWASimulator Linkage to Plink ped

--insert: insert columns/rows into a plain-text file

--linkage2boost: convert Linkage to BOOST

--linkage2ped: convert Linkage to Plink ped

--logicreg2ped: convert LogicReg to Plink ped

--mdr2boost: convert MDR to BOOST

--mdr2ped: convert MDR to Plink ped

--me2boost: convert ME to BOOST

--me2ped: convert ME to Plink ped

--other2ped: convert other format to Plink ped

--ped2beam: convert Plink ped to BEAM

--ped2bed: convert Plink ped to bed

--ped2boost: convert Plink ped to BOOST

--ped2geo: convert Plink ped to geo

--ped2gs-linkage: convert Plink ped to GWASimulator Linkage

--ped2linkage: convert Plink ped to Linkage

--ped2logicreg: convert Plink ped to LogicReg

--ped2mdr: convert Plink ped to MDR

--ped2me: convert Plink ped to ME

--ped2other: convert Plink ped to other format (indicator --file2 specifies the inputted PED)

--ped2ped: normalize genotypes (--nor), swap genotypes (--swap), generate sex randomly (--rnd-sex) or pre-process to Plink ped (--pre)

--ped2pretty: convert Plink ped to PrettyBase

--ped2svmsnp: convert Plink ped to SVMSNPs

--ped2tped: convert Plink ped to tped

--pretty2ped: convert PrettyBase to Plink ped

--space2csv: convert file with space delimiter to CSV

--splice-ped: splice two Plink ped files

--svmsnp2ped: convert SVMSNPs to Plink ped

--test-allele: test composition of heterozygotes in folder

--tped2bed: convert Plink tped to bed

--tped2ped: convert Plink tped to ped

--tped2usr-tped: convert Plink tped to user defined tped

--transpose: transpose a plain-text file

*Indicators:

--chr-no: specify chr. No. while converting to ped (default 0)

--col: specify colum No. or an interval of columns (default 0)

--delim: specify delimiter of columns for input or output file (default ' ')

--file2: specify the second input file

--hwe: cut-off of HWE (default 0.001)

--memo: specify memo (A pair of " is necessary while meeting a space in memo)

--mode: specify converting mode

--model: specify genetic model while converting to LogicReg (0-Additive, 1-Dominant, 2-Recessive; default 0)

--nor: specify to normalize output

--out: output file name

--phenotype: specify replacing phenotype for input or output file (default 0)

--pre: specify to preprocess to input file

--rnd-sex: generate gender randomly

--row: specify row No. or an interval of rows

--seed: random seed (default or 0 is current time)

--swap: swap minor and major alleles when the numbers of them are identical

Note: 1. The parameters may be arbitrary order and case insensitive.

2. The extension should be removed if the output is format Plink.

And, appending the default extensions of files is suggested except for format Plink.

I will exit after 20 seconds. ← Prevent the program from disappearing rapidly on the screen when you double-click it to run in a GUI OS.

2. A default filename will be used if the item <out> in null.
3. If the output file exists, it will be overwritten without any prompt.
4. All parameters produce a LOG file with the same main filename as the output file, but with the extension .log. The LOG file will record all information of coPLINK running. See --beam2ped in Part II.
5. Except for parameter --compare, all parameters will append their parameter name, input filename, cases count, controls count and SNPs count to the end of a statistical file named as coPLINK-RDstat.csv. See --beam2ped in Part II.

CUATION:

1) The format of the command lines in this manual is based on MS-DOS of Windows. If type the command lines in Linux, a leading string "./" need be added.
2) For readability, some TAB may be added into data or results in the examples.

2 Lists of Parameters and Indicators

coPLINK supports 45 parameters and 16 indicators. Each parameter means a main function which may derive many sub-functions from combining the indicators supported by the parameter. The parameters and indicators are listed in Table 1 and 2.

Table 1. Parameters

No.	Parameter	Supported Indicators
1	--beam2ped	--swap<br>--seed<br>--pre<br>--hwe
2	--beam2boost	--swap<br>--pre<br>--hwe
3	--bed2beam	
4	--bed2boost	--swap
5	--bed2me	
6	--bed2tped	
7	--boost2bed	--seed<br>--chr-no
8	--boost2ped	--chr-no<br>--seed
9	--compare	--file2<br>--memo<br>--mode<br>--delim<br>--col
10	--csv2space	
11	--delete	--delim<br>--row<br>--col<br>--mode
12	--geo2ped	--swap<br>--seed
13	--gs-linkage2ped	--chr-no<br>--pre<br>--hwe
14	--insert	--delim

No.	Parameter	Supported Indicators
		--mode
15	--linkage2boost	--swap
16	--linkage2ped	--chr-no<br>--pre<br>--hwe<br>--nor
17	--logicreg2ped	--model<br>--delim<br>--seed<br>--chr-no<br>--mode
18	--mdr2boost	--swap
19	--mdr2ped	--chr-no<br>--seed<br>--swap
20	--me2boost	--swap
21	--me2ped	--seed<br>--chr-no
22	--other2ped	--seed<br>--chr-no
23	--ped2beam	--swap<br>--pre<br>--hwe<br>--nor -1<br>--phenotype
24	--ped2bed	--nor<br>--pre<br>--hwe<br>--phenotype
25	--ped2boost	--swap<br>--pre<br>--hwe<br>--nor -1<br>--phenotype
26	--ped2geo	--swap<br>--pre<br>--hwe<br>--phenotype
27	--ped2gs-linkage	--swap<br>--pre<br>--hwe<br>--nor -1

No.	Parameter	Supported Indicators
		--phenotype
28	--ped2linkage	--swap<br>--nor<br>--pre<br>--hwe<br>--phenotype
29	--ped2logicreg	--swap<br>--mode<br>--model<br>--pre<br>--hwe<br>--nor -1<br>--phenotype
30	--ped2mdr	--swap<br>--pre<br>--hwe<br>--nor -1<br>--phenotype
31	--ped2me	--swap<br>--pre<br>--hwe<br>--nor -1<br>--phenotype
32	--ped2other	--file2<br>--mode<br>--phenotype
33	--ped2ped	--pre<br>--rnd-sex<br>--swap<br>--seed<br>--hwe<br>--nor<br>--phenotype
34	--ped2pretty	--nor<br>--swap<br>--pre<br>--hwe<br>--mode<br>--memo
35	--ped2svmsnp	--mode<br>--nor<br>--pre<br>--hwe

No.	Parameter	Supported Indicators
		--phenotype
36	--ped2tped	--swap<br>--mode<br>--nor<br>--pre<br>--hwe<br>--phenotype
37	--pretty2ped	--mode<br>--chr-no<br>--seed<br>--phenotype
38	--space2csv	
39	--splice-ped	-- phenotype<br>--nor<br>--file2
40	-- svmsnp2ped	--seed<br>--chr-no<br>--mode
41	--test-allele	--nor<br>--mode
42	--tped2bed	--pre<br>--phenotype<br>--hwe<br>--nor -1
43	--tped2ped	--pre<br>--nor<br>--rnd-sex<br>--seed<br>--phenotype<br>--hwe
44	--tped2usr-tped	--pre<br>--phenotype<br>--hwe<br>--nor -1
45	--transpose	--delim<br>--mode

Note: all parameters support indicator --out, although it does not list in the table.

Table 2. Indicators

No.	Indicator	Value
1	--chr-no x	1-22, X or Y.
2	--col x	m or m…n.
3	--delim x	Character.
4	- -file2 x	Filename.
5	--hwe x	Float number.
6	--memo x	Memo.
7	--model x	0, 2 or 3.
8	--mode x	Integer.
9	--nor x	Integer.
10	--out x	Filename.
11	--phenotype x	Character, usually 0, 1 or 2.
12	--pre x	Usually 0, 1 or float number.
13	--rnd-sex	
14	--row x	m or m…n.
15	--seed x	Unsigned integer
16	--swap	

Part II Specifications

All specifications of the parameters supported by coPLINK are listed here by a dictionary order. For each parameter, the functions, supported indicators and an example with several sub-examples are provided, as well as an introduction of data formats if need.

1 --beam2ped

This parameter is used to convert a file with BEAM format to a group of files with PED format. A BEAM file looks like this:

```
ID	Chr	Pos	1 1 0 1 0 1 0 1 ...
rs0	chr1	738547	2 2 1 2 0 0 0 0 ...
rs1	chr1	5597094	0 1 0 0 2 2 0 0 ...
rs2	chrX	9424115	0 2 2 0 0 0 0 0 ...
rs3	chrY	1387981	0 0 0 0 0 2 2 0 ...
```

- First row: the first 3 columns compose a tittle, and the additional columns are phenotypes represented by 0 - control and 1 - case.
- Second and subsequent rows: each row is a SNP (the first 3 columns are ID, Chr. No. and base-pair position respectively, and additional the genotypes of the SNP).
- Fourth and additional columns: each one is an individual.
- The first 4 columns are separated by TABs, and the additional columns by spaces.
- Genotype characters: 0 - Aa, 1 - aa, 2 - AA and character '-' is missing. Here, letters 'A' and 'a' are major and minor alleles respectively and the same hereinafter.

Note: a Chr. No. needs a leading string "chr" (case insensitive).

Four indicators are supported by the parameter, namely,

- --swap
  - Swap the characters of major and minor alleles when a SNP has the same numbers of major and minor alleles. For example, after swapping, the genotypes "AA AC CC" of a SNP will change into "CC CA AA", and its corresponding codes of BEAM are changed into "1 0 2" from "2 0 1".
  - In fact, the nature of a SNP is the same whether swapping or not in this case. This indicator is mainly used to analyze data such as a comparison.
- --seed
  - Not all data include Sexes such as BEAM. This indicator is used to generate Sexes randomly while converting a dataset without Sexes to

another one with Sexes, for example, converting BEAM to PED.
  - The value of this indicator is the seed of the random numbers generator. The default value is current time.
- --pre
  - Specify preprocessing to the source data.
  - A preprocessing refers to removing the individuals with alleles missing rates greater than the threshold, deleting the SNPs having MAFs less than the threshold, and discarding the SNPs failed in HWE test.
  - Its value tells the thresholds of MAF and alleles missing rate tests.
  - The default value is 0.05.
- --hwe
  - Specify the threshold of HWE.
  - This indicator need be used together with --pre.
  - If its value followed by a letter 'E' or 'e' means implementing an exact SNP test of Hardy-Weinberg Equilibrium as described in Wigginton, JE, Cutler, DJ, and Abecasis, GR (2005) A Note on Exact Tests of Hardy-Weinberg Equilibrium (American Journal of Human Genetics).
  - The default value is 0.001 (P-value).
  - "--hwe e" indicates an exact HWE test with default value.
- --out
  - Specify the output filename of PED, and usually only the main filename.

Example 1. Suppose we have a BEAM file a.txt listed as below.

```
ID          Chr     Pos     0 0 0 1 1 1
rs571227    chr1    738547  0 0 2 1 0 2
rs2827188   chr0    5597094 0 0 1 2 2 2
rs2835744   chrX    9424115 0 0 2 1 0 0
rs181146    chrY    1387981 2 2 2 2 2 -
```

a) Command "coPLINK --beam2ped a --out b" gives as follows:

```
#-------------------------------------------------------#
|         coPLINK          |      V4.0 Feb. 2020        |   ← version
|-------------------------------------------------------|
|  (C) 2020 Han-Ming LIU, GNU General Public License, V3  |
|-------------------------------------------------------|
|                 Bug-report: lhmgzjx@163.com           |
|-------------------------------------------------------|
| Please cite the paper if you used this program in your |
| research for publication.                             |
#-------------------------------------------------------#
Converting starts at 2020-3-4 22:53:3
Parameters:
```

--beam2ped a ← current parameter and the inputted file

Indicators: ← entered and default indicators

--out: b

--pre: False

--rnd-sex: True ← although the command does not specify --rnd-sex, it is forced to generate sex randomly since BEAM has no information of sex

--seed: Time ← a default value of current time was used as the seed of random number generator

--swap: False

Loading file a ...

Reading the file a.txt. Please wait ...

5... ← show the read numbers of rows dynamically (excluding null rows)

The BEAM file a read successfully.

Summary: 3 cases, 3 controls and 4 SNPs. ← information of input file

Converting ...

5 ...

The PED file saved as b.ped

4 ...

The MAP file saved as b.map

Converting ends at 2020-3-4 22:53:3

The prompting information above shows indicators --hwe, --pre, --rnd-sex, --seed and --swap that are not specified by the command. It is tells that these indicators are supported by the parameter and used the default values of following them. And, the program will print some information on the log file with the same main filename as the out file and ".log" as extension, which may be used to trace the status of coPLINK. For example, here is b.log:

```
#--------------------------------------------------------#
|          coPLINK          |        V4.0 Feb. 2020      |
|--------------------------------------------------------|
|  (C) 2020 Han-Ming LIU, GNU General Public License, V3  |
|--------------------------------------------------------|
|                Bug-report: lhmgzjx@163.com             |
|--------------------------------------------------------|
| Please cite the paper if you used this program in your |
| research for publication.                              |
#--------------------------------------------------------#
```

Converting starts at 2020-3-4 22:53:3

Parameter:

--beam2ped a

Indicators:

--out: b

--pre: False

--rnd-sex: True
--seed: Time
--swap: False
The BEAM file a read successfully. ← indicate "a.txt" to be read OK
The PED file saved as b.ped
The MAP file saved as b.map
Converting ends at 2020-3-4 22:53:3

Furthermore, the program records all information of reading data into a .csv file named as "coPLINK-RDstat.csv" to facilitate checking the results.

Created at 2020-3-4 22:53:3 via coPLINK (V4.0) by H.M. LIU et al. (Feb. 2020). Bug-report: lhmgzjx@163.com
Parameter,file,case,control,SNP
'--beam2ped,a,3,3,4

At the end of the program running, two PED files b.ped and b.map were produced.

b.ped:

```
1 1 0 0 2 1 d D d D d D D D
1 2 0 0 2 1 d D d D d D D D
1 3 0 0 1 1 D D d d D D D D
1 4 0 0 2 2 d d D D d d D D
1 5 0 0 1 2 d D D D d D D D
1 6 0 0 2 2 D D D D d D N N
```
← the missing genotype was coded as 'N N'

Note: the Individual IDs (column 2) were set serial No. and the Paternal ID (column 3) and Maternal ID (column 4) were set to 0 because of the absence of those in BEAM.

b.map:

```
chr1 rs571227   0 738547
chr0 rs2827188  0 5597094
chrX rs2835744  0 9424115
chrY rs181146   0 1387981
```

Note: the absent genetic distances in BEAM were set to 0.

b) Command "coPLINK --beam2ped a --out b --swap" will produce a Ped file as follows:

```
1 1 0 0 2 1 d D d D d D D D
1 2 0 0 1 1 d D d D d D D D
1 3 0 0 2 1 D D d d d d D D
1 4 0 0 2 2 d d D D D D D D
1 5 0 0 2 2 d D D D d D D D
1 6 0 0 1 2 D D D D d D N N
```

Compare with the previous result, the two genotypes (highlighted with red) of the third SNP has been swapped the characters.

c) Command "coPLINK --beam2ped a --out b --pre" will display like this:

…
Total 1 error(s) found after test (see Log file for details). ← tell preprocessing error count is 1
The BEAM file a read successfully.
Summary: 3 cases; 3 controls and 3 SNPs. ← indicate valid SNP of 3 after preprocessing
…

Then, the Log file b.log recorded the details of errors:

The 4-th SNP MAF tests error. ← tell the MAF test of the fourth SNP didn't pass.
Total 1 error(s) found after test.

When we enter "coPLINK --beam2ped a --out b --pre 0.4" to increase the cutoff of MAF test, the second SNP does not pass too. Similarly, if the command is changed into "coPLINK --beam2ped a --out b --pre --hwe 0.8" to increase the cutoff of HWE test, the third SNP would not pass the HWE test.

2 --beam2boost

This parameter is used to convert format BEAM to BOOST. The format of BOOST is defined an alone plain-text file, which is designed as:

```
1 2 2 0 0 0 0 2 2 1 2 0 2 2 0 1 …
0 1 2 0 1 0 0 2 2 0 2 0 1 2 2 1 …
0 2 1 0 0 0 1 0 2 0 2 1 1 2 0 2 …
1 2 1 1 1 0 1 0 2 1 2 0 2 2 0 1 …
```

- The first column is phenotypes which employ 0 and 1 to represent control and case.
- Starting from the second column, it uses the number of minor alleles to indicate a genotype. And, the genotype will be replaced with a "-1" if it is "missing".
- Each row is an individual, and each column after the second column is a SNP.
- The delimiter of the rows is a space.

The parameter supports 4 indicators (See "--beam2ped" to find their functions):

- --swap
- --pre
- --hwe
- --out
  - Specify the output filename of BOOST, and usually only the main filename.

Example 2: Suppose we have a BEAM file a.txt with the same data as example 1.

a) Command "coPLINK --beam2boost a --out b" will produce a BOOST file named as b.txt:

```
0 1 1 1 0
0 1 1 1 0
0 0 2 0 0
1 2 0 2 0
1 1 0 1 0
1 0 0 1 -1
```

b) Command "coPLINK --beam2boost a --out b --swap" will produce a BOOST file named as b.txt, which swapped the genotypes of the third SNP.

```
0 1 1 1 0
0 1 1 1 0
0 0 2 2 0
1 2 0 0 0
1 1 0 1 0
1 0 0 1 -1
```

c) Command "coPLINK --beam2boost a --out b --swap --pre" generates a BOOST file named as b.txt, which swapped the genotypes of the third SNP and removed the fourth SNP because of its failure on MAF test.

```
0 1 1 1
0 1 1 1
0 0 2 2
1 2 0 0
1 1 0 1
1 0 0 1
```

3 --bed2beam

It is used to convert PLINK Bed to BEAM format. The Bed format is binary,

includes 3 files, a .bed, a .fam and a .bim. The documents of "BED file format" and "PLINK Documentation" provided by Shaun Purcell give the detailed specifications of the format.

It supports only one indicator.

- --out
  - Specify the output filename of BEAM, and usually only the main filename.

Example 3: Suppose we have a Bed file a.bed with binary data as follows:

```
6C 1B 01 3A 0E CA 0F 3A 0A FF 07
```

The Fam file a.fam formatted with plain-text (here we gave several Family IDs and Individual IDs by design with multi-character) has the following data:

```
1r        1       0 0 1 1
2         1werwr  0 0 2 1
3         1       0 0 2 1
4         1fs     0 0 1 2
5         1       0 0 2 2
6sfsdfsd  1       0 0 1 2
```

And the corresponding a.bim file looks like this:

```
1  snp1   0  1  A  C
25 snp2   0  2  G  T
X  snp3   0  3  A  C
Y  snp4   0  4  N  C
```

a) Command "coPLINK --bed2beam a --out b" will produce a BEAM file named as b.txt:

```
ID     Chr    Pos  0 0 0 1 1 1
snp1   Chr1   1    0 0 2 1 0 2
snp2   Chr0   2    0 0 1 2 2 2    ← the invalid Chr. No. was replaced with 0
snp3   ChrX   3    0 0 2 1 0 0
snp4   ChrY   4    2 2 2 2 2 -    ← '-' indicates a missing genotype
```

Since a BEAM file does not include information of Family ID, Individual ID, Paternal ID, Maternal ID, Sex and Genetic distance, these information given by Bed format were all removed.

4 --bed2boost

This parameter is employed to convert a PLINK Bed format to BOOST.

Two indicators are supported by the parameter (See "--beam2ped" to find their functions):

- --swap
- --out
  - Specify the output filename of BOOST, and usually only the main filename.

Example 4: Suppose we have the Bed files a.bed, a.fam and a.bim, which are the same as example 3.

a) Command "coPLINK --bed2boost a --out b" will produce a BOOST file named as b.txt:

```
0 1 1 1 0
0 1 1 1 0
0 0 2 0 0
1 2 0 2 0
1 1 0 1 0
1 0 0 1 -1
```

The information except for the genotypes was saved only the phenotypes (column 1).

b) Command "coPLINK --bed2boost a --out b --swap" produced a BOOST data with swapped the genotype characters since the third SNP has the same numbers of the major and minor alleles.

```
0 1 1 1 0
0 1 1 1 0
0 0 2 2 0
1 2 0 0 0
1 1 0 1 0
1 0 0 1 -1
```

5 --bed2me

Parameter --bed2me is used to convert a PLINK bed format to ME format. A file with ME format is a single plain-text. E.g.,

```
1 1  1  1  1  ...
2 2  1  1  2  ...
```

- The first column is the serial No. of the individuals.
- The second column is phenotypes using 1 and 2 for controls and cases respectively.
- Starting from the third column, every two columns constitute an SNP.
- The genotypes of ME include 0/0 or 1/1 (AA), 1/2 or 2/1 (Aa) and 2/2 (aa). Here letters 'A' and 'a' represent major and minor alleles respectively. And, we use '-1' to indicate a missing allele.
- Each row is an individual.
- The delimiter of rows is a space.

It support --out indicator only.

- --out
    - Specify the output filename of ME, and usually only the main filename.

Example 5: Suppose we have the Bed files a.bed, a.fam and a.bim, which are the same as example 3.

a) Command "coPLINK --bed2me a --out b" will produce a ME file named as b.me:

```
1 1 1 2 1 2 1 2 1 1
2 1 1 2 1 2 1 2 1 1
3 1 1 1 2 2 1 1 1 1
4 2 2 2 1 1 2 2 1 1
5 2 1 2 1 1 1 2 1 1
6 2 1 1 1 1 1 2 -1 -1
```

6 --bed2tped

It is used to convert PLINK Bed to PLINK Tped format. The Tped format is a plain-text format, includes a .tped and a .tfam files. The documents of "PLINK Documentation" provided by Shaun Purcell give the detailed specifications of the

format.

It supports only one indicator.

- --out
  - Specify the main filename of TPED.

Example 6: Suppose we have the Bed files a.bed, a.fam and a.bim, which are the same as example 3.

a) Command "coPLINK --bed2tped a --out b" will produce a group of Tped files including b.tped and b.tfam.

b.tped:

```
1     snp1 0 1 A C A C C C A A A C C C
25    snp2 0 2 G T G T G G T T T T T T
X     snp3 0 3 A C A C C C A A A C A C
Y     snp4 0 4 C C C C C C C C C C N N
```

b.tfam

```
1r       1      0 0 1 1
2        1werwr 0 0 2 1
3        1      0 0 2 1
4        1fs    0 0 1 2
5        1      0 0 2 2
6sfsdfsd 1      0 0 1 2
```

7 --boost2bed

This parameter is used to convert a file with BOOST format to a group of PLINK Bed files.

The parameter has two indicators may be used to converting.

- --seed
  - There is no gender information in a BOOST file, so the parameter generates the information randomly. Indicator --seed sets an unsigned integer as the seed of the random number generator.
- --chr-no
  - No chromosome No. exists in BOOST, and this indicator may be employed to specify a chromosome No. for all SNPs in .bim file of BED.

- The No.es include numbers 1-22, letters 'X' and 'Y' (case insensitive).
- You may code the No. as multi-character when coding a No. with 1-22, such as "Chr2".
- A multi-character No. without 1-22 is not allowed, for example "ChrX".
- To specify the No. as X or Y, the letter must be the first character, namely, only the first character is valid.
- If a multi-character No. contains spaces, enclose it in double quotes.
- Since a genotype dataset usually employ a space as delimiters, try not to use spaces in --chr-no.

- --out
  - Specify the main filename of BED.

Example 7: Suppose we have a BOOST file a.txt with data as follows:

```
0 1 1 1 0
0 1 1 1 0
0 0 2 0 0
1 2 0 2 0
1 1 0 1 0
1 0 0 1 -1
```

a) Command "coPLINK --boost2bed a --out b" will produce a group of Bed files including b.bed, b.fam and b.bim, as listed below.

b.bed:

```
6C 1B 01 3A 0E CA 0F 3A 0A FF 07
```

b.fam:

```
1 1 0 0 2 1
1 2 0 0 2 1
1 3 0 0 2 1
1 4 0 0 1 2
1 5 0 0 1 2
1 6 0 0 1 2
```

b.bim:

```
0  snp1  0  1  d  D
0  snp2  0  2  d  D
0  snp3  0  3  d  D
0  snp4  0  4  N  D
```

b) Run the command "coPLINK --boost2bed a --out b" again, a .fam file

different from step a) was gotten.

```
1 1 0 0 1 1
1 2 0 0 1 1
1 3 0 0 1 1
1 4 0 0 2 2
1 5 0 0 2 2
1 6 0 0 1 2
```

Compare with step a), the above step gives a different sex column because it used a different seed of the random number generator.

c) Command "coPLINK --boost2bed a --out b --seed 1586" may produce a .fam file with a new sex column.

```
1 1 0 0 2 1
1 2 0 0 2 1
1 3 0 0 2 1
1 4 0 0 2 2
1 5 0 0 1 2
1 6 0 0 2 2
```

d) Re-run the Command "coPLINK --boost2bed a --out b --seed 1586".

```
1 1 0 0 2 1
1 2 0 0 2 1
1 3 0 0 2 1
1 4 0 0 2 2
1 5 0 0 1 2
1 6 0 0 2 2
```

Compare with step c), the .fam file produced by this step is the same as step c) because of the same seed of the random number generator.

e) In step a), all the chromosome No.es was set to 0 at the first column since the BOOST file a.txt has no the No.es. Command "coPLINK --boost2bed a --out b --chr-no "Chr 2"" may specify the No.es as "Chr 2" in b.bim. Here, the "Chr 2" is enclosed in double quotes because it includes a space.

```
Chr 2   snp1   0   1   d   D
Chr 2   snp2   0   2   d   D
Chr 2   snp3   0   3   d   D
Chr 2   snp4   0   4   N   D
```

8 --boost2ped

The parameter is used to convert a file with BOOST format to a group of PLINK Ped files.

It supports two indicators (see parameter --boost2bed).

- --seed
- --chr-no
- --out
  - Specify the main filename of PED.

Example 8: Suppose we have the same BOOST file a.txt used for example 7.

a) Command "coPLINK --boost2ped a --out b" will produce a group of Ped files including b.ped and b.map

b.ped:

```
1 1 0 0 2 1 d D d D d D D D
1 2 0 0 1 1 d D d D d D D D
1 3 0 0 2 1 D D d d D D D D
1 4 0 0 2 2 d d D D d d D D
1 5 0 0 2 2 d D D D d D D D
1 6 0 0 2 2 D D D D d D N N
```

b.map:

```
0 SNP1 0 0
0 SNP2 0 0
0 SNP3 0 0
0 SNP4 0 0
```

9 --compare

This parameter is used to compare two files, which is the extension of the OS comparison. Its command line usually looks like this:

coPLINK --compare <in> --file2 <file2> [--out [out]] [indicator1 [value1]] …

The value <in> is the compared file while <file2> is the comparing file. Their extensions cannot be ignored.

The comparison mode of this parameter is categorized into two classes: by order and by reference. The mode by reference can compare by specifying a column as a criterion. For example, two plain-text files ('↲' repsenting a null row) have different row-order in column 2:

1.dat:

0 SNP4 0 0
0 SNP3 0 0
↲
0 SNP2 0 1
0 SNP1 0 0

2.dat:

0 SNP1 0 0
↲
0 SNP2 0 0
0 SNP3 0 0
0 SNP4 0 0

If we compare the files by order, it will report five differences. However, if the mode by reference with column 2 of 1.dat is used as a criterion, the comparison will search the matched value in 2.dat according to the value of column 2 in 1.dat and thus find only one difference between the files and report the difference locating at column 4, row 4 of 1.dat and row 3 of 2.dat.

It supports indicators as listed below.

- --file2
  - Specify the comparing filename (with extension).
  - Use a pathname if needed. And, enclose its value in double quotes if it contains spaces.
- --memo
  - Specify a string as memo recorded into the output file. If it contains spaces, enclose it in double quotes.
  - The default is "compared filename-comparing filename".
- --mode
  - Comparison mode including: 0 - row-by-row for plain-text files, 1 - byte-by-byte for binary files, 2 - row-by-row with records of detailed differences for plain-text files, 3 - byte-by-byte with records of detailed differences for binary files.
- --delim
  - Specify the delimiter of the two files
  - The default is space and/or TAB. The compared file is used as the

reference file.

- The leading and tailing spaces/TABs will be removed and the other spaces/TABs will be replaced with one space, when a space/TAB was specified as a delimiter.

- --col
  - Specify the reference column No. when performing a comparison by reference.
  - The default is 0.
  - If a No. is less than or equal to 0, it means a comparison by order, otherwise means a comparison by reference.
  - When performing a comparison by reference, rows (including null rows) with fewer columns than the No. will not be compared but counted.
- --out
  - Specify the output filename to save comparison results, whose default extension is .csv.
  - The comparison results will be written into the .csv file by appending mode.

Example 9: Suppose we have two plain-text files and two binary files, and the differences were highlighted in red as follows:

a.txt (plain-text):

```
0 1 1 1 0
0 1 1 1 0
0 0 2 0 0
1 2 0 2 0
1 1 0 1 0
1 0 0 1 -1
```

b.txt (plain-text):

```
0 1 1 1 0
0 1 1 1 0
0 0 2 0 1
1 2 0 2 0
1 1 0 1 0
1 0 0 1 -1
2 2 0 2 1
```

c.bed:

```
6C 1B 01 3A 0E CA 0F 3A 0A FF 07
```

d.bed:

6C 1B 01 3A 0E CA 0F 3A 0A FA 07 8A

a) Command "coPLINK --compare a.txt --file2 b.txt --out e" compares a.txt with b.txt, and produces the comparison results named as e.csv.

```
...
Indicators:
    --col: 0
    --delim: ' '
    --file2: b.txt
    --memo: a.txt-b.txt ← default memo
    --mode: 0           ← comparison mode 0: row-by-row for plain-text files
    --out: e
Comparing file a.txt with b.txt by order ...
The sizes of file1 a.txt and file2 b.txt are 61 and 71 bytes, respectively.
    6 ...  ← display progress dynamically
Compared rows/bytes are 6 which of 1 rows/bytes are different.    ← prompt results
...
```

Open the result file e.csv.

Created at 2020-3-6 14:35:13 via coPLINK (V4.0) by ...

Mode,	File1,	File2,	Compared,	Diff Cnt,	Size1/*#row1,	Size2/*row2##,	column###,	Memo
Text,	a.txt,	b.txt,	6,	1,	61,	71,	-,	Summary of a.txt-b.txt

Note: #If a number in that column has a '*', it means that the number represents a row No. or a byte No.; ##The fourth column is the size/row No. of/in compared file, and the fifth column is that of/in comparing file. ###This column records the column No. where the difference exists.

The fourth and fifth columns record the size of compared file (Size1) and the size of comparing file (Size2). The results tell that the command compared 6 rows and find 1 row is different.

The comparison will terminate while encountering one of the EOFs of the two input files.

b) Command "coPLINK --compare c.bed --file2 d.bed --out e --mode 1 --memo "Bin cmp"" compares c.ped with d.ped. And produces the comparison results as listed below.

Mode,	File1,	File2,	Compared,	Diff Cnt,	Size1/*row1,	Size2/*row2,	column,	Memo
Text,	a.txt,	b.txt,	6,	1,	61,	71,	-,	Summary of a.txt-b.txt
****************************** ← dividing line between two alone commands								
Binary,	c.bed,	d.bed,	11,	1,	11,	12,	-,	Summary of Bin cmp

Similar to step a), the command did not compare 12 bytes but 11 bytes.

c) Command "coPLINK --compare c.bed --file2 d.bed --out e --mode 3 --memo "Bin cmp"" compares c.bed with d.bed. And produces the comparison results as listed below.

Mode,	File1,	File2,	Compared,	Diff Cnt,	Size1/*row1,	Size2/*row2,	column,	Memo
Text,	a.txt,	b.txt,	6,	1,	61,	71,	-,	Summary of a.txt-b.txt
******************************								
Binary,	c.bed,	d.bed,	11,	1,	11,	12,	-,	Summary of Bin cmp
******************************								
Binary,	c.bed,	d.bed,	10,	1,	*10,	*10,	-,	Bin cmp
Binary,	c.bed,	d.bed,	11,	1,	11,	12,	-,	Summary of Bin cmp

The result tells the 10th byte is different between the two files.

d) Command "coPLINK --compare a.txt --file2 b.txt --out e --mode 2" compares a.txt with b.txt, and produces the comparison results named as e.csv including the position of difference.

Mode,	File1,	File2,	Compared,	Diff Cnt,	Size1/*row1,	Size2/*row2,	column,	Memo
Text,	a.txt,	b.txt,	6,	1,	61,	71,	-,	Summary of a.txt-b.txt
******************************								
Binary,	c.bed,	d.bed,	11,	1,	11,	12,	-,	Summary of Bin cmp
******************************								
Binary,	c.bed,	d.bed,	10,	1,	*10,	*10,	-,	Bin cmp
Binary,	c.bed,	d.bed,	11,	1,	11,	12,	-,	Summary of Bin cmp
******************************								
Text,	a.txt,	b.txt,	3,	1,	*3,	*3,	5,	a.txt-b.txt
Text,	a.txt,	b.txt,	6,	1,	61,	71,	-,	Summary of a.txt-b.txt

The results indicate the difference between a.txt and b.txt is at row 3, column 5.

e) Suppose we have two plain-text files, and the differences were highlighted in red as follows:

f.csv:

```
0,SNP4,0,0
0,SNP3,0,0
0,SNP2,0,1
0,SNP1,0,0
```

g.csv:

0,SNP1,0,0
0,SNP2,0,0
0,SNP3,0,0
0,SNP4,0,0

Firstly, we type command "coPLINK --compare f.csv --file2 g.csv --out e" to compare the files by mode 0.

Mode,	File1,	File2,	Compared,	Diff Cnt,	Size1/*row1,	Size2/*row2,	column,	Memo
...								
******************************								
Text,	f.csv,	g.csv,	4,	4,	44,	44,	-,	Summary of f.csv-g.csv

The result file shows there are 4 differences between the two files. Then, we change the command into "coPLINK --compare f.csv --file2 g.csv --out e --col 2 --delim , --mode 2".

Mode,	File1,	File2,	Compared,	Diff Cnt,	Size1/*row1,	Size2/*row2,	column,	Memo
...								
******************************								
Text,	f.csv,	g.csv,	2,	1,	*3,	*2,	4,	f.csv-g.csv
Text,	f.csv,	g.csv,	4,	1,	44,	44,	-,	Summary of f.csv-g.csv

Above resulta show 1 difference was found in the comparison, which is at the column 4 of the row 3 of the compared file f.csv and the same column of the row 2 of the comparing file g.csv.

Suppose the data of g.csv changed into as follows:

0,SNP1,0,0
0,SNP5,0,0
0,SNP3,0,0
0,SNP4,0,0

Run the command into "coPLINK --compare f.csv --file2 g.csv --out e --col 2 --delim , --mode 2" again.

Mode,	File1,	File2,	Compared,	Diff Cnt,	Size1/*row1,	Size2/*row2,	column,	Memo
...								
******************************								
Text,	f.csv,	g.csv,	2,	1,	Not found,	*2,	-,	f.csv-g.csv
Text,	f.csv,	g.csv,	4,	2,	*3,	Not found,	-,	f.csv-g.csv
Text,	f.csv,	g.csv,	4,	2,	44,	44,	-,	Summary of f.csv-g.csv

Now, the results indicate 2 differences were found: one is the row 2 of the

comparing file was not found in the compared file, the other is the row 3 of the compared file was not found in the comparing file.

10 --csv2space

This parameter is used to convert a file with a ',' as the delimiters to with a space as the delimiters.

The indicator includes:

- --out
  - Specify the main filename of output with a space as the delimiters.

Example 10: Suppose we have a file f.csv as listed in example 9.

a) Command "coPLINK --csv2space f.csv --out f.txt" will generate a file with a space as the delimiter.

```
0 SNP4 0 0
0 SNP3 0 0
0 SNP2 0 1
0 SNP1 0 0
```

11 --delete

This parameter is used to delete rows or columns from a plain-text file.

The indicators supported by the parameter include:

- --delim
  - Specify the delimiter of the rows in the input file.
  - It is for deleting columns only.
  - The escape character "\t" represents a TAB character.
  - The default value is a space.
  - If the source file (input file) has several spaces or/and TABs between two columns, it is only one wiil be reserve. The default reserved delimiter is a space, and you can reserve a TAB by using "--delim \t".
- --row
  - Specify the row No. to delete.
  - A negative No. means a row No. in reverse order.

- The symbol "..." is used to represent a range of rows. For example, "--row 3…6" represents to delete continuous 4 rows starting from row 3 (inclusive). Caution: The range symbol may be corrected as an ellipsis by Word.
- The negative No.es may be used in a range.
- If a false range was specified, only the beginning row of the range is deleted. For example, the "--row 5…3" is equivalent to "--row 5".
- The default value is 0 which indicates no rows will be removed.
- If the specified row No. exceeds the maximum row No. of the input file, no rows will be deleted.

- --col
  - Specify the column No. to delete.
  - A negative No. means a column No. in reverse order.
  - The symbol "…" is used to represent a range of columns.
  - The negative No.es may be used in a range.
  - If a false range was specified, only the beginning column of the range is deleted.
  - The default value is 0 which indicates no columns will be removed.
  - If the specified column No. exceeds the maximum column No. of the input file, no columns will be deleted.
- --mode
  - Specify a deleting mode.
  - The default value is 0 which indicates do not delete the null rows at the end of the input file.
  - The value 1 tells the parameter to delete the null rows at the end of the input file.
- --out
  - Specify the output filename of deleted rows or/and columns.

Example 11: Suppose we have a data file a.dat as listed below.

```
1,2,3,4,5,6,7,8,9,10,11,12,13,14,15,16,17,18,19,20
2,2,3,4,5,6,7, 8,9    ,10,    11,12,13      ← has several spaces
3,2,3,4,5
4,2,3,4,5,    6,      7 ,8,9                ← has several spaces and a TAB
5,2,3,4,5,6,7,8,9,10,11,12,13,14,15,16,17,18,19,20
6,2,3,4,5,6,7,8,9,10,11,      12,13         ← has a TAB
7,2,3,4,5,6,7,8,9,10,11,12,13,14,15,16,17,18
8,2 ,3    ,          4,     5               ← has several spaces and TABs
9,2,3,4,5,6,7,8,9,10,11,12
10,2,3,4,5,6,7,8,9,10,11,12,13,14
↵
↵
↵
```

Note: there are 3 null rows repsented by '↵' at the end of the file.

a) Command "coPLINK --delete a.dat --row 2 --col 11 --out b.txt --delim ," will generate a file b.txt.

```
1,2,3,4,5,6,7,8,9,10,12,13,14,15,16,17,18,19,20
3,2,3,4,5
4,2,3,4,5,6,7,8,9
5,2,3,4,5,6,7,8,9,10,12,13,14,15,16,17,18,19,20
6,2,3,4,5,6,7,8,9,10,12,13
7,2,3,4,5,6,7,8,9,10,12,13,14,15,16,17,18
8,2,3,4,5
9,2,3,4,5,6,7,8,9,10,12
10,2,3,4,5,6,7,8,9,10,12,13,14
↵
↵
↵
```

The result shows the second row was deleted, and the column 11 in all rows having column 11 was deleted. Furthermore, all spaces and TABs were removed.

b) The result of command "coPLINK --delete a.dat --row -2 --col -11 --out b.txt --delim , --mode 1" with a negative row No. and a negative column No..

```
1,2,3,4,5,6,7,8,9,11,12,13,14,15,16,17,18,19,20
2,2,3,4,5,6,7,8,9,11,12,13
3,2,3,4,5
4,2,3,4,5,6,7,8,9
5,2,3,4,5,6,7,8,9,11,12,13,14,15,16,17,18,19,20
6,2,3,4,5,6,7,8,9,11,12,13
7,2,3,4,5,6,7,8,9,11,12,13,14,15,16,17,18
8,2,3,4,5
10,2,3,4,5,6,7,8,9,11,12,13,14
↵
```

Note: a null row at the end of the result has been retained (Windows only).

Indicator "--mode 1" removed the null rows at the end of the source file, and a null row at the end of the result was retained to indicate the end of the file when the deleting was run on Windows OS.

c) The result of command "coPLINK --delete a.dat --row 2...-2 --col -11...13 --out b.txt --delim , --mode 1" with ranges of row and column No..

```
1,2,3,4,5,6,7,8,9,14,15,16,17,18,19,20
10,2,3,4,5,6,7,8,9,14
↵
```

12 --geo2ped

This parameter is used to convert a group of files with GEO format to PED format. The GEO format is a user-defined plain-text format, which includes two files: one is .geo and the other is .map.

A .geo file looks like this:

```
1,1,0,1,0,1,0,1,…
2,2,1,2,0,0,0,0,…
0,1,0,0,2,2,0,0,…
0,2,2,0,0,0,0,0,…
0,0,0,0,0,2,2,0,…
```

- The first row: Phenotypes. 0 - control, 1 - case.
- Starting from the second row: Each one is a SNP. 0 - Aa, 1 - aa, 2 - AA, <0 - missing.
- The delimiter among columns is a ','.

A .map file includes the information of SNPs in the .geo file:

- The first row is the tittle: Affymetrix ID, Target Description, Gene Title and Gene Symbol.
- The four columns of the file are Base-pair Position, Genetic Distance, Chr. No. and RS# or SNP ID, respectively.
- The delimiter among columns is a space.

The indicators supported by the parameter include (See previous specifications to find the functions of the top 2 indicators):

- -- swap
- -- seed
- --out
  - Specify the output main filename of PED format.

Example 12: Suppose we have a group of GEO files with main filename a.

a.geo:

```
0,0,0,1,1,1
0,0,2,1,0,2
0,0,1,2,2,2
0,0,2,1,0,0
```

2,2,2,2,2,-1

a.map:

Affymetrix ID,Target Description,Gene Title,Gene Symbol
1,0,1,SNP1
2,0,0,SNP2
3,0,X,SNP3
4,0,Y,SNP4

a) Command "coPLINK --geo2ped a --out b" will generate a group of files with PED format.

b.ped:

```
1 1 0 0 1 1 d D d D d D D D
1 2 0 0 2 1 d D d D d D D D
1 3 0 0 1 1 D D d d D D D D
1 4 0 0 2 2 d d D D d d D D
1 5 0 0 1 2 d D D D d D D D
1 6 0 0 1 2 D D D D d D N N
```

b.map:

```
1 SNP1 0 1
0 SNP2 0 2
X SNP3 0 3
Y SNP4 0 4
```

b) Command "coPLINK --geo2ped a --out b --swap" with indicator --swap:

b.ped:

```
1 1 0 0 2 1 d D d D d D D D
1 2 0 0 2 1 d D d D d D D D
1 3 0 0 1 1 D D d d d d D D
1 4 0 0 2 2 d d D D D D D D
1 5 0 0 1 2 d D D D d D D D
1 6 0 0 2 2 D D D D d D N N
```

b.map:

```
1 SNP1 0 1
0 SNP2 0 2
X SNP3 0 3
Y SNP4 0 4
```

13 --gs-linkage2ped

This parameter is used to convert a file with GS-linkage format to PED format. The GS-linkage format is a single document plain-text format, generated by GWASimulator, and includes a .dat file usually.

A GS-linkage file looks like this:

```
1 1 0 0 1 1 1 2 1 2 …
2 2 0 0 1 1 1 2 1 2 …
1 3 0 0 1 1 2 2 0 2 …
1 4 0 0 1 2 1 1 1 1 …
…
```

- Each row is an individual.
- The first column: Family ID.
- The second column: Individual ID.
- The third column: Paternal ID.
- The fourth column: Maternal ID.
- The fifth column: Sex. 1 - male, 2 - female.
- The sixth column: Phenotypes. 0 - unknown, 1 - control and 2 - case.
- Starting from the seventh column: Every two columns are a SNP. 0 - missing allele, 1 - allele 0, 2 - allele 1.
- The delimiter among columns is a space.

The indicators supported by the parameter include (See previous specifications to find the functions of the top 3 indicators):

- --chr-no
- --pre
- --hwe
- --nor
    - Specify to normalize the output file.
    - "Normalization" refers to normalize genotypes, which (1) uses letters "D", "d" and "N" to represent the major allele, minor allele and missing allele respectively, (2) uses a space as the delimiters among columns and (3) treats the first character of an allele with multi-character as the allele character.
- --out
    - Specify the output main filename of PED format.

Example 13: Suppose we have a GS-linkage file a.dat.

```
1r        1       0 0 1 1 1 2 1 2 2 1 2 2
2         1werwr  0 0 1 1 1 2 1 2 2 1 2 2
3         1       0 0 1 1 2 2 2 2 2 2 2 2
4         1fs     0 0 1 2 1 1 1 1 1 1 2 2
5         1       0 0 1 2 2 1 1 1 1 2 2 2
6sfsdfsd  1       0 0 1 2 2 2 1 1 1 2 0 2
```

a) Command "coPLINK --gs-linkage2ped a --out b" will generate a group of files with PED format.

b.ped:

```
1r        1      0 0 1 1 d D d D D d D D
2         1werwr 0 0 1 1 d D d D D d D D
3         1      0 0 1 1 D D D D D D D D
4         1fs    0 0 1 2 d d d d d d D D
5         1      0 0 1 2 D d d d d D D D
6sfsdfsd  1      0 0 1 2 D D d d d D N D
```

b.map:

```
0 SNP1 0 0
0 SNP2 0 0
0 SNP3 0 0
0 SNP4 0 0
```

b) Type command "coPLINK --gs-linkage2ped a --out b --nor" to generalize the .ped file.

```
1r        1      0 0 1 1 d D D d D d D D
2         1werwr 0 0 1 1 d D D d D d D D
3         1      0 0 1 1 D D d d D D D D
4         1fs    0 0 1 2 d d D D d d D D
5         1      0 0 1 2 D d D D d D D D
6sfsdfsd  1      0 0 1 2 D D D D d D N D
```

c) Use command "coPLINK --gs-linkage2ped a --out b --pre --chr-no 2" to preprocess the .ped file and specify a chromosome No. for .map file.

b.ped:

```
1r  1      0 0 1 1  d D d D D d
2   1werwr 0 0 1 1  d D d D D d
3   1      0 0 1 1  D D D D D D
4   1fs    0 0 1 2  d d d d d d
```

```
5    1     0 0 1 2 D d d d d D
```

b.map:

```
2 SNP1 0 0
2 SNP2 0 0
2 SNP3 0 0
```

It shows the fourth SNP and the sixth sample have not passed the preprocessing and the chromosome No. was set to 2.

14 --insert

The parameter may insert rows or columns in a plain-text file. The format of using this parameter is:

```
coPLINK --insert <in> ...
```

It is different from the previous command lines that the <in> is not the inserted file, but an arguments file. The inserted file is specified in the arguments file. An arguments file tells what and where to insert. If the <in> you gave does not exist, a template file of arguments named coPLINK-insert.txt will be produced at current path/directory. The template explains the format of an argument line and the usage of the items in the line, and gives some examples. It looks like this:

```
# This section is for "--ped2other" and "--insert", which gives the row(s)/col.(s) inserted into
#     the destination file(s).      ← symbol '#' means current line is a comment
# Format:
#     <row/col. No.>,[filename],[begin],[delimiter],[direction],[item1],[item2],...
#     row/col.No. - Position in <filename>.It cannot be null. 0, null or a nonnumeric
#          character represents being next the row / col. from above line. A negative number
#          means inverse order. When the specified position exceeds the maximum
#          row/col. No., the inserted text will be appended to the end.
#     filename - Filename of the inserted file. (including the path if need).Null means
#          using the filename specified in the nearest line from above (for "--ped2other")
#          or the first one (for "--insert").
#     begin - Beginning position of the row/column in "other" file(s). Null, 0 or a negative
#          number indicates the head of the row/column.
#     delimiter - Delimiter of columns. Null means a space, and "\," represents a comma
#          and "\t" a tab.
#     direction - Direction of the inserted texts in <filename>. 0 or null means in column,
#          otherwise in row.
```

```
#     item1, item2, ... - Inserted items. Their number may be or not limited, and a
#           regular expression may be used here. Read the manual for details, please.
#     CAUTION:
#           a. This section may be null and be allowed any number of lines.
#           b. The null lines in destination needs be counted in [begin].
#           c. The [filename] cannot be null in the first argument line, and the first one is
#               accepted only for "--insert".
#           d. For an argument line here, only ONE wildcard can be included in ONE item.
#
# Example:
#     Let Alleles.txt listed in section [VECTOR] as the "other" file, we may code like this:
#         1,alleles.txt,1, ,1,Family,Individual,Paternal,Maternal,Sex,A1,A2,A3,A4,Phenotype
#         3,,1,,1
#     Or,
#         1,alleles.txt,1,,1,Family,Individual,Paternal,Maternal,Sex,A#,Phenotype
#         3,,1,,1
#     At last, we put the lines at the next line of [INSERT BEGIN].

[INSERT BEGIN]      ← section name, do not rename or remove it
        ← put argument lines here
[INSERT END]        ← section name, do not rename or remove it
```

The inserted rows/columns are putted between the section name [INSERT BEGIN] and [INSERT END]. A line lies between the two section-names is named as an argument line. Each line indicates one row/column will be inserted into the source file specified by the item [filename] in the line. Please note that the parameter allows only one destination. If more than one destination is given, the destination located at the first line will be accepted. You can insert multiple rows/columns by putting multiple argument lines which are order insensitive. The number of inserted rows/columns is not limited. The null arguments lines and spaces or TABs invovled in an arguments line will be ignored by the program.

The parameter supports two wildcards to allow inserting some regular items.

- #: Serial No. staring from 1. The maximum No. depends on the capacity of the inserted row or column. For example, suppose the inserted file has a maximum column No. of 5, the item "A#,Class" for inserting a row means " A1,A2,A3,A4,Class".
- ^: Insert alternately the two characters following the wildcard until the end of the inserted row or column. The default values of these two characters are '0' and '1'. If the number of characters following the wildcard is less than 1, the default values will be used. For example, suppose the source file (inserted file) is the same as above. The item "A^,Class" for inserting a row means " A1,A2,A1,A2,Class".

Sine a ',' is used as the delimiter in arguments line, you can employ an escape character '\,' to indicate an inserted comma. And, the escape characters of the wildcards are \# and \^ which allow you to insert symbols '#' and/or '^'. Only one wildcard is allowed in one argument line. If there are multiple wildcards, they will be treated as ordinary characters except for the first one.

If you specify a row or column No. exceeding the maximum row or column No. of the result, it means to append the inserted row or column to the last one.

It is not allowed that the number of rows of an inserted column is greater than the number of rows of the destination.

The parameter supports indicators as listed below.

- --delim
  - Specify the delimiter of the source file.
  - The default value is a space or an admixture of a space and a TAB. The escape character '\t' represents a TAB.
  - Note that the delimiter of the destination is specified in the first arguments line.
- --mode
  - Specify whether reserves the null rows located at the end of the source file.
  - The value of 0 is default, which means to reserve the null rows. And, the value of 1 indicates to remove these null rows.
- --out
  - Specify the destination filename (including path and extension).

Example 14: Suppose we have a file a.dat with data as listed below.

```
1,2,3,4,5,6,7,8,9,10,11,12,13,14,15,16,17,18,19,20
2,2,3,4,5,6,7, 8,9    ,10,    11,12,13        ← has spaces
3,2,3,4,5
4,2,3,4,5,    6,       7 ,8,9                 ← has several spaces and a TAB
5,2,3,4,5,6,7,8,9,10,11,12,13,14,15,16,17,18,19,20
6,2,3,4,5,6,7,8,9,10,11,       12,13          ← has a TAB
7,2,3,4,5,6,7,8,9,10,11,12,13,14,15,16,17,18
8,2 ,3    ,            4,     5               ← has several spaces and TABs
9,2,3,4,5,6,7,8,9,10,11,12
10,2,3,4,5,6,7,8,9,10,11,12,13,14
↵
↵
↵
```

Note: there are 3 null rows represented by '↵' at the end of the file.

a) We want to insert the following row before the second row, and to name the destination as b.txt.

1,2,3,4,5,6,7,8,9,10,11,12,13,14,15,16,17,18,19,20

We design firstly an arguments file named ins.txt like this:

```
[INSERT BEGIN]
2,a.dat,1,\,,1,#      ← the delimiter is a comma, so we use the escape character '\,'
[INSERT END]
```

Then, we type command "coPLINK --insert ins.txt --out b.txt --delim ," to insert the row.

```
1,2,3,4,5,6,7,8,9,10,11,12,13,14,15,16,17,18,19,20
1,2,3,4,5,6,7,8,9,10,11,12,13,14,15,16,17,18,19,20
2,2,3,4,5,6,7, 8,9 ,10, 11,12,13
3,2,3,4,5
4,2,3,4,5, 6, 7 ,8,9
5,2,3,4,5,6,7,8,9,10,11,12,13,14,15,16,17,18,19,20
6,2,3,4,5,6,7,8,9,10,11, 12,13
7,2,3,4,5,6,7,8,9,10,11,12,13,14,15,16,17,18
8,2 ,3 , 4, 5
9,2,3,4,5,6,7,8,9,10,11,12
10,2,3,4,5,6,7,8,9,10,11,12,13,14
↵
↵
↵
```

b) We change the ins.txt as follows and rename it as ins1.txt:

```
[INSERT BEGIN]
10,a.dat,1,\,,0,Row^abcd,Sumary
[INSERT END]
```

c) Command "coPLINK --insert ins1.txt --out b.txt --mode 1 --delim ," will insert a column before the 10th column and remove the null rows at the end of the file a.dat.

```
1,2,3,4,5,6,7,8,9,Rowacd,10,11,12,13,14,15,16,17,18,19,20
2,2,3,4,5,6,7, 8,9 ,Rowbcd,10, 11,12,13
3,2,3,4,5,,,,,Rowacd
4,2,3,4,5, 6, 7 ,8,9,Rowbcd
5,2,3,4,5,6,7,8,9,Rowacd,10,11,12,13,14,15,16,17,18,19,20
6,2,3,4,5,6,7,8,9,Rowbcd,10,11, 12,13
```

```
7,2,3,4,5,6,7,8,9,Rowacd,10,11,12,13,14,15,16,17,18
8,2 ,3 , 4, 5,,,,,Rowbcd
9,2,3,4,5,6,7,8,9,Rowacd,10,11,12
10,2,3,4,5,6,7,8,9,Sumary,10,11,12,13,14
↵
```

Like parameter --delete, indicator --mode 1 reserved a null row at the end of the file (Windows only) to indicate the EOF (End Of File).

d) Command "coPLINK --insert ins2.txt --out b.csv --mode 1 --delim ," with the following arguments file ins2.txt will add a row headings, a column headings and a summary column to a.dat.

```
[INSERT BEGIN]
1,a.dat,2,\,,0,R#
100,,2,\,,0,Sum#
1, ,1,\,,1,Row\#,X#,Summary
[INSERT END]
```

100,,2,\,,0,Sum# ← column No. 100 exceeds the total number of the columns, so it is appended to the end. And, [filename] is null means it is the same as above.

1, ,1,\,,1,Row\#,X#,Summary ← escape character '\#'

Then, we open the file b.csv by Excel or other electronic sheet tool. From the result, we can learn the null rows at the end of a.dat were not added to the row headings (i.e., were removed).

Row#	X1	X2	X3	X4	X5	X6	X7	X8	X9	X10	X11	X12	X13	X14	X15	X16	X17	X18	X19	X20	Summary
R1	1	2	3	4	5	6	7	8	9	10	11	12	13	14	15	16	17	18	19	20	Sum1
R2	2	2	3	4	5	6	7	8	9	10	11	12	13								Sum2
R3	3	2	3	4	5																Sum3
R4	4	2	3	4	5	6	7	8	9												Sum4
R5	5	2	3	4	5	6	7	8	9	10	11	12	13	14	15	16	17	18	19	20	Sum5
R6	6	2	3	4	5	6	7	8	9	10	11	12	13								Sum6
R7	7	2	3	4	5	6	7	8	9	10	11	12	13	14	15	16	17	18			Sum7
R8	8	2	3	4	5																Sum8
R9	9	2	3	4	5	6	7	8	9	10	11	12									Sum9
R10	10	2	3	4	5	6	7	8	9	10	11	12	13	14							Sum10

15 --linkage2boost

This parameter is used to convert a file with the Linkage Pedigree (pre MAKEPED) format to the BOOST format. The Linkage format includes two files: one is .ped (data file) and the other is .info (locus information or marker information file).

A Linkage .ped file looks like this:

```
1 1 0 0 1 1 1 2 3 4 …
2 2 0 0 1 1 1 2 3 4 …
1 3 0 0 1 1 2 2 0 4 …
1 4 0 0 1 2 1 1 3 3 …
…
```

- Each row is an individual.
- The first column: Family ID.
- The second column: Individual ID.
- The third column: Paternal ID. 0 - unknown.
- The fourth column: Maternal ID. 0 - unknown.
- The fifth column: Sex. 1 - male, 2 - female.
- The sixth column: Phenotypes. 0 - unknown, 1 - control and 2 - case.
- Starting from the seventh column: Every two columns are a SNP, using bases to represent alleles, usually 0 - missing base, 1 - base 'A', 2 - base 'C', 3 - base 'G' and 4 - base 'T'.
- The delimiter among columns is a space.

And, an .info file looks like this:

```
IGR1118a_1 274044
IGR1119a_1 274541
…
```

An .info file records the positions of base-pairs. It includes two columns, the first one is the SNP labels and the second one is the positions of the base-pairs in the SNPs.

The indicators supported by the parameter include (See previous specifications to find the functions of --swap indicator):

- --swap
- --out
  - Specify the output main filename of the BOOST format.

Example 15: Suppose we have a Linkage file a.ped

```
1 1 1 1 1 1 1 3 2 4 3 1 4 4
2 1 1 1 2 1 1 3 4 2 3 1 4 4
1 2 1 1 1 1 3 3 2 2 3 3 4 4
1 2 2 2 2 2 1 1 4 4 1 1 4 4
5 1 1 1 1 2 3 1 4 4 1 3 4 4
6 1 1 1 1 2 3 3 4 4 1 3 0 4
```

And its base-pair position file a.info is

rs571227 738547
rs2827188 5597094
rs2835744 9424115
rs181146 1387981

a) Command "coPLINK --linkage2boost a --out b" will produce the b.txt with the BOOST format.

0 1 1 1 0
0 1 1 1 0
0 0 2 0 0
1 2 0 2 0
1 1 0 1 0
1 0 0 1 -1

b) The result tells that the SNP 3 has the same numbers of the major and minor alleles. Command "coPLINK --linkage2boost a --out b --swap" may swap its codes of major and minor alleles.

0 1 1 1 0
0 1 1 1 0
0 0 2 2 0
1 2 0 0 0
1 1 0 1 0
1 0 0 1 -1

16 --linkage2ped

This parameter is used to convert a file with the Linkage Pedigree format to the PED format.

The indicators supported by the parameter include (See previous specifications to find the functions of the top 4 indicators):

- --chr-no
- --pre
- --hwe
- --nor
- --out
  - Specify the output main filename of PED format.

Example 16: Suppose we have a group of Linkage files are the same as Example 15.

a) Command "coPLINK --linkage2ped a --out b" will generate a group of files with PED format.

b.ped:

```
1 1 1 1 1 1 A G C T G A T T
2 1 1 1 2 1 A G T C G A T T
1 2 1 1 1 1 G G C C G G T T
1 2 2 2 2 2 A A T T A A T T
5 1 1 1 1 2 G A T T A G T T
6 1 1 1 1 2 G G T T A G N T
```

b.map:

```
0 rs571227   0 738547
0 rs2827188 0 5597094
0 rs2835744 0 9424115
0 rs181146   0 1387981
```

b) Type command "coPLINK --linkage2ped a --out b --nor" to generalize the .ped file.

```
1 1 1 1 1 1 d D d D D d D D
2 1 1 1 2 1 d D D d D d D D
1 2 1 1 1 1 D D d d D D D D
1 2 2 2 2 2 d d D D d d D D
5 1 1 1 1 2 D d D D d D D D
6 1 1 1 1 2 D D D D d D N D
```

c) Use command "coPLINK --linkage2ped a --out b --pre --chr-no 2" to preprocess the .ped file and specify a chromosome No. for .map file.

b.ped:

```
1 1 1 1 1 1  A G C T G A
2 1 1 1 2 1  A G T C G A
1 2 1 1 1 1  G G C C G G
1 2 2 2 2 2  A A T T A A
5 1 1 1 1 2  G A T T A G
```

b.map:

```
2 rs571227   0 738547
2 rs2827188 0 5597094
2 rs2835744 0 9424115
```

It shows the fourth SNP and the last individual have not passed the preprocessing and the chromosome No. was set to 2.

17 --logicreg2ped

This parameter is used to convert a file with the LogicReg format to the PED format. The LogicReg is a single document format.

A LogicReg file stores the genotypes which uses 0 or 1 to represent a genotype of an allele. It looks like this:

```
0 0 1 0 1 0 1 0 0 …
0 0 1 0 1 0 1 0 0 …
0 0 0 1 1 0 0 0 0 …
1 1 1 0 0 1 1 0 0 …
1 0 1 0 0 0 1 0 0 …
1 0 0 0 0 0 1 - - …
…
```

Or

```
0 1 1 1 0 …
0 1 1 1 0 …
0 0 1 0 0 …
1 1 0 1 0 …
1 1 0 1 0 …
1 0 0 1 - …
…
```

- Each row is an individual.
- The first column: Phenotypes. Here, we set 0 - control and 1 - case. The phenotypes may the values after Logit transforming.
- Starting from the second column (letters 'A' and 'a' are major and minor alleles respectively): it has 3 types of encoding based on genetic models,
  - Additive genetic model: Every two columns are a SNP. For each SNP, 0 0 - AA, 0 1 - Aa and aA, 1 1 - aa. And, we set "-" to represent a missing allele.
  - Dominant genetic model: Each column is a SNP. 0 - AA, 1 - Aa, aA and aa. And, we set "-" to represent a missing genotype.
  - Recessive genetic model: Each column is a SNP. 0 - AA, Aa and aA, 1 - aa. And, we set "-" to represent a missing genotype.
- The delimiter among columns is a space.

The indicators supported by the parameter include (See previous specifications to find the functions of the top 3 indicators):

- --delim
- --seed
- --chr-no
- --mode
  - Specify whether the phenotypes are Logitted. 0 - no, 1 - yes.
  - The default value is 0.
- ---model
  - Specify the genetic model of the inputted data. 0 - additive (default), 1 - dominant, 2 - recessive.
- --out
  - Specify the output main filename of the PED format.

Example 17: Suppose we have a LogicReg a.txt.

```
0 0 1 0 1 0 1 0 0
0 0 1 0 1 0 1 0 0
0 0 0 1 1 0 0 0 0
1 1 1 0 0 1 1 0 0
1 0 1 0 0 0 1 0 0
1 0 0 0 0 0 1 - -
```

a) Suppose the genetic model of the data is additive. Use command "coPLINK --logicreg2ped a.txt --out b --chr-no 2" to generate a group of PED files with a main filename b.

b.ped:

```
1 1 0 0 2 1 D d D d D d D D
1 2 0 0 2 1 D d D d D d D D
1 3 0 0 1 1 D D d d D D D D
1 4 0 0 1 2 d d D D d d D D
1 5 0 0 2 2 D d D D D d D D
1 6 0 0 1 2 D D D D D d N N
```

b.map:

```
2 SNP1 0 0
2 SNP2 0 0
2 SNP3 0 0
2 SNP4 0 0
```

b) Here is another LogicReg file named as c.txt.

```
-13.8155095579638 1 1 1 0
-13.8155095579638 1 1 1 0
-13.8155095579638 0 1 0 0
+13.8155095579350 1 0 1 0
+13.8155095579350 1 0 1 0
+13.8155095579350 0 0 1 -
```

The pattern of the file tells that it may be a LogicReg file with loggited phenotypes and its genetic model cannot be confirmed.

- Suppose its genetic model is additive, we type command "coPLINK --logicreg2ped c.txt --out b --chr-no 2 --mode 1".

b.ped:

```
1 1 0 0 1 1 d d d D
1 2 0 0 2 1 d d d D
1 3 0 0 2 1 D d D D
1 4 0 0 1 2 d D d D
1 5 0 0 1 2 d D d D
1 6 0 0 2 2 D D d N
```

b.map:

```
2 SNP1 0 0
2 SNP2 0 0
```

- Suppose its genetic model is dominant, we change the command to "coPLINK --logicreg2ped c.txt --out b --chr-no 2 --mode 1 --model 1".

b.ped:

```
1 1 0 0 1 1 d d d d d d D D
1 2 0 0 1 1 d d d d d d D D
1 3 0 0 1 1 D D d d D D D D
1 4 0 0 1 2 d d D D d d D D
1 5 0 0 1 2 d d D D d d D D
1 6 0 0 1 2 D D D D d d N N
```

b.map:

```
2 SNP1 0 0
2 SNP2 0 0
2 SNP3 0 0
2 SNP4 0 0
```

- Suppose its genetic model is recessive, we change the command to "coPLINK --logicreg2ped c.txt --out b --chr-no 2 --mode 1 --model 2".

b.ped:

```
1 1 0 0 2 1 d d d d d d D D
1 2 0 0 1 1 d d d d d d D D
1 3 0 0 2 1 D D d d D D D D
1 4 0 0 2 2 d d D D d d D D
1 5 0 0 1 2 d d D D d d D D
1 6 0 0 1 2 D D D D d d N N
```

b.map:

```
2 SNP1 0 0
2 SNP2 0 0
2 SNP3 0 0
2 SNP4 0 0
```

18 --mdr2boost

This parameter is used to convert a file with MDR format to the BOOST format. The MDR format includes either a single document or two documents.

The data file is a required document with a default of .dat as its extension, which looks like this:

```
0	1	…	1	1
0	1	…	0	0
1	1	…	2	1
0	1	…	1	0
```

- The delimiter in a MDR file is a TAB.
- The first column: Phenotypes. Here we use 0 - control, 1 - case.
- The additional columns: Genotypes. Each column is a SNP. These values range from 0 to the MAXLOCIVALUE defined by MDR. Here we use 0 - AA, 1 - Aa or aA, 2 - aa, -1 - missing.
- Each row is a separate individual.

Except for the .dat file, a .map file may be included as an optional file. It allows names to be specified for the variables in the data file. Usually, the first column is the chromosome number, second column the name and third column the base-pair

position of the SNP.

The indicators supported by the parameter include (See previous specifications to find the functions of the first indicator):

- --swap
- --out
  - Specify the output main filename of the BOOST format.

Example 18: Suppose we have a group of files with MDR fromat.

a.dat:

0	1	1	1	0
0	1	1	1	0
0	0	2	0	0
1	2	0	2	0
1	1	0	1	0
1	0	0	1	-1

a.map:

1	rs571227	0
0	rs2827188	0
X	rs2835744	0
Y	rs181146	0

a) Use command "coPLINK --mdr2boost a --out b" to generate a BOOST file b.txt.

```
0 1 1 1 0
0 1 1 1 0
0 0 2 0 0
1 2 0 2 0
1 1 0 1 0
1 0 0 1 -1
```

b) Type command "coPLINK --mdr2boost a --out b --swap" to generate a new BOOST file b.txt.

```
0 1 1 1 0
0 1 1 1 0
0 0 2 0 0
1 2 0 2 0
1 1 0 1 0
```

```
1 0 0 1 -1
```

19 --mdr2ped

This parameter is used to convert a file with MDR format to the PED format.

The indicators supported by the parameter include (See previous specifications to find the functions of the top 2 indicators):

- --swap
- --seed
- --chr-no
  - It is valid when the source does not include a MAP file.
- --out
  - Specify the output main filename of the BOOST format.

Example 19: Suppose we have the same group of files with MDR format as Example 18.

a) Use command "coPLINK --mdr2ped a --out b" to generate a group of PED files with main filename b.

b.ped:

```
1 1 0 0 2 1 d D d D d D D D
1 2 0 0 1 1 d D d D d D D D
1 3 0 0 1 1 D D d d D D D D
1 4 0 0 1 2 d d D D d d D D
1 5 0 0 1 2 d D D D d D D D
1 6 0 0 2 2 D D D D d D N N
```

b.map:

```
1 rs571227  0 0
0 rs2827188 0 0
X rs2835744 0 0
Y rs181146  0 0
```

b) Type command "coPLINK --mdr2ped a --out b --swap" to generate a new group of files with PED format.

b.ped:

```
1 1 0 0 1 1 d D d D d D D D
1 2 0 0 1 1 d D d D d D D D
1 3 0 0 1 1 D D d d d d D D
1 4 0 0 2 2 d d D D D D D D
1 5 0 0 2 2 d D D D d D D D
1 6 0 0 2 2 D D D D d D N N
```

b.map:

```
1 rs571227  0 0
0 rs2827188 0 0
X rs2835744 0 0
Y rs181146  0 0
```

c) Change above command into "coPLINK --mdr2ped a --out b --chr-no 2" to generate another group of files with PED format.

b.ped:

```
1 1 0 0 1 1 d D d D d D D D
1 2 0 0 1 1 d D d D d D D D
1 3 0 0 1 1 D D d d D D D D
1 4 0 0 1 2 d d D D d d D D
1 5 0 0 1 2 d D D D d D D D
1 6 0 0 2 2 D D D D d D N N
```

b.map:

```
1 rs571227  0 0
0 rs2827188 0 0
X rs2835744 0 0
Y rs181146  0 0
```

The results tell us that the .ped file is the same as step a) (except the sex column generated randomly) and, the .map the same too because the source files include a .map file resulting in the indicator --chr-no to be invalid.

20 --me2boost

This parameter is used to convert a file with ME format to the BOOST format. The ME is a single document format, which looks like this:

```
0 1   …   1   1
```

0 1 … 0 0
1 1 … 2 1
0 1 … 1 0

- The delimiter in a ME file is a space.
- The first column: Serial No. of the individuals.
- The second column: Phenotypes. 1 - control, 2 - case.
- The additional columns: Genotypes. Every two columns are a SNP. 0/0 or 1/1 - AA, 1/2 - Aa, 2/1 - aA, 2/2 - aa. Here we use, -1/-1 - missing.
- Each row is a separate individual.

The indicators supported by the parameter include (See previous specifications to find the functions of the first indicator):

- --swap
- --out
  - Specify the output main filename of the BOOST format.

Example 20: Suppose we have a.me file with ME format.

1 1 2 1 2 1 1 2 1 1
2 1 2 1 1 2 1 2 1 1
3 1 1 1 2 2 1 1 1 1
4 2 2 2 1 1 2 2 1 1
5 2 1 2 1 1 2 1 1 1
6 2 1 1 1 1 2 1 -1 1

a) Use command "coPLINK --me2boost a.me --out b" to generate a BOOST file b.txt.

0 1 1 1 0
0 1 1 1 0
0 0 2 0 0
1 2 0 2 0
1 1 0 1 0
1 0 0 1 -1

b) Use command "coPLINK --me2boost a.me --out b --swap" to generate a new BOOST file b.txt.

0 1 1 1 0
0 1 1 1 0
0 0 2 2 0
1 2 0 0 0
1 1 0 1 0

```
1 0 0 1 -1
```

21 --me2ped

This parameter is used to convert a file with ME format to PED format.

The indicators supported by the parameter include (See previous specifications to find the functions of the top 4 indicators):

- --seed
- --chr-no
- --swap
- --out
    - Specify the output main filename of PED format.

Example 21: Suppose we have the file with ME format is the same example as 20.

```
1 1 2 1 2 1 1 2 1 1
2 1 2 1 1 2 1 2 1 1
3 1 1 1 2 2 1 1 1 1
4 2 2 2 1 1 2 2 1 1
5 2 1 2 1 1 2 1 1 1
6 2 1 1 1 1 2 1 -1 1
```

a) Use command "coPLINK --me2ped a.me --out b --chr-no X" to generate two files with PED format.

b.ped:

```
1 1 0 0 2 1 d D d D D d D D
1 2 0 0 1 1 d D D d D d D D
1 3 0 0 2 1 D D d d D D D D
1 4 0 0 1 2 d d D D d d D D
1 5 0 0 1 2 D d D D d D D D
1 6 0 0 1 2 D D D D d D N D
```

b.map:

```
X SNP1 0 0
X SNP2 0 0
X SNP3 0 0
X SNP4 0 0
```

b) Use command "coPLINK --me2ped a.me --out b --chr-no X --swap" to generate a new .ped file.

```
1 1 0 0 1 1 d D d D d D D D
1 2 0 0 1 1 d D D d d D D D
1 3 0 0 1 1 D D d d d d D D
1 4 0 0 1 2 d d D D D D D D
1 5 0 0 2 2 D d D D D d D D
1 6 0 0 2 2 D D D D D d N D
```

22 --other2ped

The tools of analyzing genotypes are developing continuously, which may lead to developing formats. How to follow to the developing formats is an interesting significantly work. This parameter gives you an opportunity to use your imagination to convert one or more files without limited formats to a group of PED files. The format of using this parameter is:

```
coPLINK --other2ped <in> ...
```

It is like the command line of parameter --insert that the <in> is not the "other" file but an arguments file. And, the usage of the parameter is similar to the --insert too.

- The "other" files are specified in an arguments file which tells how to generate the components of the destination (PED files) from which formats of the "other" files and where in the "other" files.
- If the <in> you typed does not exist, a template file of arguments named coPLINK-2ped.txt will be produced at current path/directory. The template explains the format of an argument line and the usage of the items involed in the line, and gives some examples. It looks like this:

```
# coPLINK (V4.0) by H.M. LIU et al. (Feb. 2020). Bugs report: lhmgzjx@163.com
# Arguments for parameter "--other2ped"    ← symbol '#' means current line is a comment
# Note:
# 1. Start with "#" comments a row.
# 2. Section names need be enclosed by a pair of [].
# 3. A null line will be ignored.
# 4. It represents absent if an argument line is not given.
# 5. All spaces/tabs in the argument line will be ignored, except for a delimiter.
# 6. The section names cannot be deleted or renamed, even if it is null.
# 7. To "--ped2other", the genotype matrix in PED must be stored as a whole into
```

```
#       "other" file, that is, it cannot have other rows or columns in its area, but
#       it can be transposed.
# 8. To "--ped2other", the column in the MAP must be stored as an entire row or
#       column into "other" file, that is, there cannot be other rows or columns in
#       the middle of it, but it can be turned into a row, and each column does not need
#       to be stored continuously.

# This section specifies the top 6 columns in PED and the whole 4 columns in MAP.
# Each argument line specifies a column in PED/MAP.
# Format:
#     <type>,[row/col. No.],[begin], [delimiter],[direction],[filename]
#     type - Type of a column. It may be "fam"(Family ID), "ind"(Individual ID), "pat"
#         (Paternal ID), "mat"(Maternal ID), "sex"(Sex), "phe"(Phenotype), "chr"(Chromosome),
#         "snp"(rs# or snp identifier), "gen"(Genetic distance) and "pos"(Base-pair position).
#         If the type "sex" is not given, will generate randomly.
#     row/col. No. - Row/column No. in [filename]. 0, null or a nonnumeric character represents
#         being next the row/col. from above line. A negative number means inverse order.
#     begin - Beginning position of the row/column in [filename]. Null, 0 or a negative number
#         indicates the head of the row/column.
#     delimiter - Delimiter of columns in [filename]. Null means a space/tab will be used,
#         and "\," represents a comma while "\t" is a tab (tab is used to "--ped2other").
#         Only the first character is valid when meeting more than 1.
#     direction - Direction of the data in [filename]. 0 or null means in column,
#         otherwise in row.
#     filename - Filename including the type (including the path if need). Null means
#         using the filename specified in the nearest line from above.
#     CAUTIONS:
#         a. There are 10 argument lines at most in this section. The excess will be ignored.
#         b. The <type> cannot be null.
#         c. The [filename] in the first line cannot be null.
#         d. It is case insensitive.
#         e. To "--other2ped", the null rows in [filename] will be skipped, but needs be counted
#             in argument lines.
#         f. To "--ped2other", the null rows in "other" file(s) need be counted in argument
#             lines.
#Example:
#     We have two files as follows,
#        Alleles.txt:
#        Family Individual Paternal Maternal Sex A1 A2 A1 A2 Phenotype
#        1 1 0 0 1 A A G T 1
#        <--- null line
#        2 1 0 0 1 A C T G 2
#        3 1 0 0 1 C C - G 2
#        End
```

```
#     and,
#         Snps.dat:
#         Platform,Illumina,Agilent
#         Chr,1,1
#         Rs#,snp1,snp2
#         Material,lung,musle
#         <--- null line
#         Genetic distance,0,0
#         Position,5000650,5000830
#     Then, 10 argument lines may be coded as
#         Fam,1,2, ,,alleles.txt
#         Ind,2,2
#         Pat,3,2
#         Mat,4,2
#         Sex,5,2
#         Phe,10,2
#         Chr,2,2,\,,1,snps.dat
#         Snp,3,2,\,,1
#         Gen,6,2,\,,1
#         Pos,7,2,\,,1
#     Or,
#         Fam,1,2, ,,alleles.txt
#         Ind,0,2
#         Pat,0,2
#         Mat,0,2
#         Sex,0,2
#         Phe,-1,2
#         Chr,2,2,\,,1,snps.dat
#         Snp,0,2,\,,1
#         Gen,6,2,\,,1
#         Pos,0,2,\,,1
#     At last, we put the 10 lines at the next line of [VECTOR BEGIN].

[VECTOR BEGIN]      ← section name, do not rename or remove it
    Fam,        ← the type names of columns in the PED were given by the template, if the "other"
                files have not a column, delete or comment it
    Ind,
    Pat,
    Mat,
    Sex,
    Phe,
    Chr,
    Snp,
    Gen,
```

```
    Pos,

[VECTOR END]        ← section name, do not rename or remove it

# This section specifies the rectangle area of the genotypes in the source file for PED.
# Format:
#     <filename>,[left],[top], [delimiter],[SNP rows/col.s],[character count],[missing],[case],
[ctrl],[male],[female],[AA],[Aa],[aa]
#     filename - Filename including the genotypes (adding the path if need). It cannot be null.
#     left - Left column No. of the rectangle in the source file. 1 will be used while meeting
#         null, 0 or a negative number.
#     top - Top row No. of the rectangle in the source file. 1 will be used while meeting null,
#         0 or a negative number.
#     delimiter - Delimiter of columns in <filename>. Null means a space, and "\," represents
#         a comma while "\t" is a tab(tab is used to "--ped2other").
#     SNP rows/col.s - Number of rows/col.s in <filename>. 0, null or out of 0~3 means
#         every two columns is a SNP; 1 is each column a SNP; 2 is every two rows a SNP;
#         and 3 is each row a SNP.
#     character count - Number of characters in a genotype. 1 or 2 is allowed. 0, null or
#         out of 1 and 2 means 2. Its value will be fixed to 2 when [SNP rows/col.s] = 0 or 2.
#     missing - String representing missing alleles. Null means PLINK's missing characters.
#     case - Character of cases. The first character will be used only. Null means using PLINK's.
#     ctrl - Character of controls. The first character will be used only. Null means using PLINK's.
#     male - Character of males. The first character will be used only. Null means using PLINK's.
#     female - Character of females. The first character will be used only. Null means using
#         PLINK's.
#     AA - Characters of genotype AA. When [character count] = 1, only the first character
#         is valid and null is 0. When [character count] = 2, only the first 2 characters are
#         valid and null or only one character means the two characters are the same as
#         the first character in [Aa]. The item is case sensitive.
#     Aa - Characters of genotype Aa. When [character count] = 1, only the first character is
#         valid and null is 1. When [character count] = 2, only the first 2 character are valid
#         and the first one is major allele, the other is minor allele; And, null or only one
#         character means the characters in source are used. The item is case sensitive.
#     aa - Characters of genotype aa. When [character count] = 1, only the first character
#         is valid and null is 2. When [character count] = 2, only the first 2 characters are
#         valid and null or only one character means the two characters are the same as
#         the second character in [Aa]. The item is case sensitive.
#     CAUTION:
#         a. This section cannot be null, and only the first line is valid while being more than 1.
#         b. To "--other2ped", the null rows in [filename] will be skipped, but needs be counted
#             in argument lines.
#         c. To "--ped2other", the null rows in "other" file(s) need be counted in argument
#             lines.
```

```
#
# Example:
#     Let Alleles.txt listed in section [VECTOR] as the source file, we may code like this:
#         alleles.txt,6,2, ,0,2,-,2,1,1,2
#     At last, we put the line at the next line of [MATRIX BEGIN].

[MATRIX BEGIN]      ← section name, do not rename or remove it
        ← put argument line here
[MATRIX END]        ← section name, do not rename or remove it
```

Notes:

- Read the specifications in the template carefully.
- The arguments lines of the components of PED files such as Family ID, Individual ID, and Chr.-Number need be putted between the section-names [VECTOR BEGIN] and [VECTOR END], while the arguments line of the genotypes in .ped file should be putted between [MATRIX BEGIN] and [MATRIX END].
- A line lies between the two section-names is named as an argument line.
- The null arguments lines and the spaces or TABs involved in an arguments line will be ignored by the program.
- For VETOR section:
    - A repeated type name is not allowed.
    - The columns in the destination (PED files) may come from different "other" files, which are specified by the item [filename]. The [filename]s except the first one may be null which means using the same filename above line.
    - If the type Sex is not specified, the program will generate sexes randomly. The seed of the generator may be specified by indicator --seed.
    - The escape character "\," represents a comma.
- The genotypes are considered a matrix, specified in the section MATTRIX.
    - Only one line is allowed, namely, the genotypes cannot be separated into several "other" files. If there are multiple lines in the section, only the first one is accepted.
    - Item [SNP rows/col.s] is used to specify the construct of the genotypes of a SNP in the "other" file including 0 - every two columns are a SNP, 1 - each column a SNP, 2 - every two rows a SNP and 3 - each row a SNP. Note that the value of the item may be 1 or 3 only when item [character count] was set to 1, and the case [character count] = 2 is similar.
- If the parameter throws any error or produces incorrect results, check the arguments file.

The indicators supported by the parameter include (See previous specifications to find the functions of the top first indicator):

- --seed
- --chr-no
  - If the "other" files do not include Chr. No., use the indicator to specify one. Otherwise, it will be ignored.
- --out
  - Specify the output main filename of PED format.

Example 22: Suppose we have a group of files with MDR format are the same as example 19.

a.dat (the first column is phenotypes, and each additional column a SNP. The delimiter is a TAB):

```
0 1  1  1  0
0 1  1  1  0
0 0  2  0  0
1 2  0  2  0
1 1  0  1  0
1 0  0  1  -1
```

a.map (the first column is Chr. No., second column the name and third column the base-pair position of the SNP. The delimiter is also a TAB.):

```
1  rs571227   0
0  rs2827188  0
X  rs2835744  0
Y  rs181146   0
```

a) We design the arguments file (named as a.txt):

```
[VECTOR BEGIN]  ← <type>,[row/col. No.],[begin], [delimiter],[direction],[filename]
phe,   1,   1,   \t,   0,   a.dat
chr,   1,   1,   \t,   ,    a.map
snp    ← all items are null after <type> means column No. is next type CHR, the whole
         column, delimiter is a space or TAB and filename the same as above
pos
[VECTOR END]
[MATRIX BEGIN]      ← <filename>,[left],[top], [delimiter], [SNP rows/col.s], [character
                      count],[missing],[case],[ctrl],[male], [female],[AA],[Aa],[aa]
a.dat,2,1, \t,1,1,-1,1,0,,,0,1,2
[MATRIX END]
```

b) Use command "coPLINK --other2ped a.txt --out b" to generate two files with PED format.

b.ped:

```
1 1 0 0 1 1 d D d D d D D D
1 2 0 0 1 1 d D d D d D D D
1 3 0 0 2 1 D D d d D D D D
1 4 0 0 1 2 d d D D d d D D
1 5 0 0 2 2 d D D D d D D D
1 6 0 0 2 2 D D D D d D N N
```

b.map:

```
1 rs571227  0 0
0 rs2827188 0 0
X rs2835744 0 0
Y rs181146  0 0
```

Compare to example 19, the results above are the same.

c) Some examples of arguments files:

Tped to ped:

```
[VECTOR BEGIN]
    Fam,1,,,,1.tfam
    Ind
    Pat
    Mat
    Sex
    Phe
    Chr,1,,,,1.tped
    Snp
    Gen
    Pos
[VECTOR END]
[MATRIX BEGIN]
1.tped,5,1, ,3,2
[MATRIX END]
```

Boost to ped:

```
[VECTOR BEGIN]
    Phe,1,1,,,1.txt
[VECTOR END]
[MATRIX BEGIN]
1.txt,2,1, ,1,1,-1,1,0,,,0,1,2
```

```
[MATRIX END]
```

Beam to ped:

```
[VECTOR BEGIN]
    Snp,1,2,\t,,1.txt
   Chr,0,2,\t
     Pos,0,2,\t
    phe,1,4,,1
  [VECTOR END]
[MATRIX BEGIN]
1.txt,4,2, ,3,1,-,1,0,,,2,0,1
[MATRIX END]
```

Me to ped:

```
  [VECTOR BEGIN]
      Phe,2,2,,,me.txt
  [VECTOR END]
  [MATRIX BEGIN]
me.txt,3,2, ,0,2,-1,2,1,,,11,12,22
[MATRIX END]
```

Geo to ped:

```
[VECTOR BEGIN]
      Phe,1,1,\,,1,1.geo
      Chr,3,2,\,,,1.gmap
      Snp,4,2,\,
      Gen,2,2,\,
      Pos,1,2,\,
[VECTOR END]
  [MATRIX BEGIN]
1.geo,1,2, \,,3,1,-1,1,0,,,2,0,1
[MATRIX END]
```

23 --ped2beam

This parameter is used to convert a file with PED format to BEAM format.

The indicators supported by the parameter include (See previous specifications to find the functions of the top 3 indicators):

- --swap
- --pre
- --hwe
- --phenotype
  - Specify the phenotypes of inputted PED to a constant.
- --nor -1
  - Tell the program not to count the numbers of major and minor alleles thus to reduce run-time.
  - Used only when the source is a normalized data.
- --out
  - Specify the output main filename of BEAM format.

Example 23: Suppose we have the files with PED format.

a.ped:

```
1 1 1 1 1 1 A T G C T A C C
2 1 1 1 2 1 A T C G T A C C
1 2 1 1 1 1 T T G G T T C C
1 2 2 2 2 2 A A C C A A C C
5 1 1 1 1 2 T A C C A T C C
6 1 1 1 1 2 T T C C A T - C
```

a.map:

```
1 rs571227  0 738547
0 rs2827188 0 5597094
X rs2835744 0 9424115
Y rs181146  0 1387981
```

a) Use command "coPLINK --ped2beam a --out b" to generate BEAM file b.txt.

ID	Chr	Pos	0 0 0 1 1 1
rs571227	Chr1	738547	0 0 2 1 0 2
rs2827188	Chr0	5597094	0 0 1 2 2 2
rs2835744	ChrX	9424115	0 0 2 1 0 0
rs181146	ChrY	1387981	2 2 2 2 2 -

b) Command "coPLINK --ped2beam a --out b --swap" can swap genotypes of the third SNP above.

ID	Chr	Pos	0 0 0 1 1 1
rs571227	Chr1	738547	0 0 2 1 0 2
rs2827188	Chr0	5597094	0 0 1 2 2 2
rs2835744	ChrX	9424115	0 0 1 2 0 0

rs181146	ChrY	1387981	2 2 2 2 2 -

c) Command "coPLINK --ped2beam a --out b --pre" uses --pre to preprocess the data.

ID	Chr	Pos	0 0 0 1 1
rs571227	Chr1	738547	0 0 1 2 0
rs2827188	Chr0	5597094	0 0 1 2 2
rs2835744	ChrX	9424115	0 0 2 1 0

The fourth SNP and the sixth individual were removed from the data because of the errors of MAF and HWE test and of alleles missing rate test in an individual respectively.

24 --ped2bed

This parameter is used to convert a file with PED format to PLINK BED format. PLINK has the same function, but the following features may sometimes not satisfy demands for some operation such as an algorithm comparison.

- Sort .bim file by chromosome No.;
- Change chromosome No. X and Y into 23 and 24 respectively;
- Exit when .ped has more than 2 missing allele characters in one SNP.

This parameter does not sort .bim file and thus keeps the same SNP order with the source, does not change the chromosome No., and supports 0, - and N (case insensitive) as missing allele characters in one SNP. Furthermore, it supports normalizing allele characters and preprocessing while converting.

The indicators supported by the parameter include (See previous specifications to find the functions of the top 3 indicators):

- --nor
- --pre
- --hwe
- --phenotype
  - Specify the phenotypes of inputted PED to a constant.
- --out
  - Specify the output main filename of BED format.

Note: if the value of '--nor' is greater than 0, the parameter will produce normalized PED files when producing BED files.

Example 24: Suppose we have the files with PED format have a little different from example 23.

a1.ped:

```
1 1 1 1 1 1 A T G C T A C C
2 1 1 1 2 1 A T C G T A C C
1 2 1 1 1 1 T T G G T T C C
1 2 2 2 2 2 A A C C A A C N
5 1 1 1 1 2 T A C C A T C C
6 1 1 1 1 2 T T C C A T - C
```

a1.map:

```
1 rs571227  0 738547
0 rs2827188 0 5597094
X rs2835744 0 9424115
Y rs181146  0 1387981
```

a) Use command "coPLINK --ped2bed a1 --out b" to generate a group of BED files with main filename b.

b.bed:

```
6C 1B 01 3A 0E CA 0F 3A 0A 7F 07
```

b.bim:

```
1   rs571227    0   738547   A   T
0   rs2827188   0   5597094  G   C
X   rs2835744   0   9424115  A   T
Y   rs181146    0   1387981  N   C
```

b.fam:

```
1 1 1 1 1 1
2 1 1 1 2 1
1 2 1 1 1 1
1 2 2 2 2 2
5 1 1 1 1 2
6 1 1 1 1 2
```

b) Use command "coPLINK --ped2bed a1 --out b --nor --pre 0.2" to re-generate BED files with main filename b.

b.bed:

6C 1B 01 3A 0E CA 0F CA 0A

b.bim:

1	rs571227	0	738547	d	D
0	rs2827188	0	5597094	d	D
X	rs2835744	0	9424115	D	d

b.fam:

1 1 1 1 1 1
2 1 1 1 2 1
1 2 1 1 1 1
1 2 2 2 2 2
5 1 1 1 1 2
6 1 1 1 1 2

25 --ped2boost

This parameter is used to convert a file with PED format to BOOST format.

The indicators supported by the parameter include (See previous specifications to find the functions of the top 3 indicators):

- --swap
- --pre
- --hwe
- --phenotype
  - Specify the phenotypes of inputted PED to a constant.
- --nor -1
  - Tell the program not to count the numbers of major and minor alleles thus to reduce run-time.
  - Used only when the source is a normalized data.
- --out
  - Specify the output main filename of BOOST format.

Example 25: Suppose we have the files with PED format are the same as example 24.

a) Use command "coPLINK --ped2boost a1 --out b" to generate a BOOST file b.txt.

0 1 1 1 0
0 1 1 1 0

0 0 2 0 0
1 2 0 2 -1
1 1 0 1 0
1 0 0 1 -1

b) Use command "coPLINK --ped2boost a1 --out b --swap" to generate a swapped BOOST file b.txt.

0 1 1 1 0
0 1 1 1 0
0 0 2 2 0
1 2 0 0 -1
1 1 0 1 0
1 0 0 1 -1

c) Use command "coPLINK --ped2boost a1 --out b --pre" to generate a preprocessed BOOST file b.txt.

0 1 1 1
0 1 1 1
0 0 0 0
1 1 2 1

26 --ped2geo

This parameter is used to convert a file with PED format to GEO format.

The indicators supported by the parameter include (See previous specifications to find the functions of the top 3 indicators):

- --swap
- --pre
- --hwe
- --phenotype
  - Specify the phenotypes of inputted PED to a constant.
- --out
  - Specify the output main filename of GEO format.

Example 26: Suppose we have the files with PED format are the same as example 24.

a) Use command "coPLINK --ped2geo a1 --out b" to generate a group of GEO files with a main filename b.

b.geo:

```
0,0,0, 1,1, 1
0,0,2, 1,0, 2
0,0,1, 2,2, 2
0,0,2, 1,0, 0
2,2,2,-1,2,-1
```

b.map:

```
Affymetrix ID, Target Description, Gene Title,  Gene Symbol
738547,        0,                  1,           rs571227
5597094,       0,                  0,           rs2827188
9424115,       0,                  X,           rs2835744
1387981,       0,                  Y,           rs181146
```

b) Use command "coPLINK --ped2geo a1 --out b --swap" to generate a swapped .geo file.

b.geo:

```
0,0,0, 1,1, 1
0,0,2, 1,0, 2
0,0,1, 2,2, 2
0,0,1, 2,0, 0
2,2,2,-1,2,-1
```

c) Use command "coPLINK --ped2geo a1 --out b --pre" to generate a new group of GEO files after preprocessing.

b.geo:

```
0,0,0,1
0,0,2,0
0,0,1,2
0,0,2,0
```

b.map:

```
Affymetrix ID,  Target Description, Gene Title,  Gene Symbol
738547,         0,                  1,           rs571227
5597094,        0,                  0,           rs2827188
9424115,        0,                  X,           rs2835744
```

27 --ped2gs-linkage

This parameter is used to convert a file with PED format to GS-linkage format. Here, we use a minor allele in PED as the 'allele0' of GS-linkage and a major allele as the 'allele1'.

The indicators supported by the parameter include (See previous specifications to find the functions of the top 3 indicators):

- --swap
- --pre
- --hwe
- --phenotype
  - Specify the phenotypes of inputted PED to a constant.
- --nor -1
  - Tell the program not to count the numbers of major and minor alleles thus to reduce run-time.
  - Used only when the source is a normalized data.
- --out
  - Specify the output main filename of GS-linkage format.

Example 27: Suppose we have the files with PED format are the same as example 24.

a) Use command "coPLINK --ped2gs-linkage a1 --out b" to generate a GS-linkage file b.dat.

```
1 1 1 1 1 1 1 2 1 2 2 1 2 2
2 1 1 1 2 1 1 2 2 1 2 1 2 2
1 2 1 1 1 1 2 2 1 1 2 2 2 2
1 2 2 2 2 2 1 1 2 2 1 1 2 0
5 1 1 1 1 2 2 1 2 2 1 2 2 2
6 1 1 1 1 2 2 2 2 2 1 2 0 2
```

b) Use command "coPLINK --ped2gs-linkage a1 --out b --swap" to generate a swapped GS-linkage file b.dat.

```
1 1 1 1 1 1 1 2 1 2 1 2 2 2
2 1 1 1 2 1 1 2 2 1 1 2 2 2
1 2 1 1 1 1 2 2 1 1 1 1 2 2
1 2 2 2 2 2 1 1 2 2 2 2 2 0
5 1 1 1 1 2 2 1 2 2 2 1 2 2
6 1 1 1 1 2 2 2 2 2 2 1 0 2
```

c) Use command "coPLINK --ped2gs-linkage a1 --out b --pre" to generate a new

GS-linkage file after preprocessing.

```
1 1 1 1 1 1 1 2 1 2 2 1
2 1 1 1 2 1 1 2 2 1 2 1
1 2 1 1 1 1 2 2 1 1 2 2
5 1 1 1 1 2 2 1 2 2 1 2
```

28 --ped2linkage

This parameter is used to convert a file with PED format to Linkage format.

The indicators supported by the parameter include (See previous specifications to find the functions of the top 4 indicators):

- --swap
- --nor
- --pre
- --hwe
- --phenotype
  - Specify the phenotypes of inputted PED to a constant.
- --out
  - Specify the output main filename of Linkage format.

Example 28: Suppose we have the files with PED format are the same as example 24.

a) Use command "coPLINK --ped2linkage a1 --out b" to generate a group of Linkage files with a main filename b.

b.ped:

```
1 1 1 1 1 1 1 4 3 2 4 1 2 2
2 1 1 1 2 1 1 4 2 3 4 1 2 2
1 2 1 1 1 1 4 4 3 3 4 4 2 2
1 2 2 2 2 2 1 1 2 2 1 1 2 0
5 1 1 1 1 2 4 1 2 2 1 4 2 2
6 1 1 1 1 2 4 4 2 2 1 4 0 2
```

b.info:

```
rs571227    738547
rs2827188   5597094
rs2835744   9424115
rs181146    1387981
```

b) Use command "coPLINK --ped2linkage a1 --out b --swap" to generate a swapped .ped Linkage file.

1 1 1 1 1 1 1 4 3 2 1 4 2 2
2 1 1 1 2 1 1 4 2 3 1 4 2 2
1 2 1 1 1 1 4 4 3 3 1 1 2 2
1 2 2 2 2 2 1 1 2 2 4 4 2 0
5 1 1 1 1 2 4 1 2 2 4 1 2 2
6 1 1 1 1 2 4 4 2 2 4 1 0 2

c) Use command "coPLINK --ped2linkage a1 --out b --pre --nor" to generate a new group of Linkage files.

b.ped:

1 1 1 1 1 1 1 3 1 3 3 1
2 1 1 1 2 1 1 3 3 1 3 1
1 2 1 1 1 1 3 3 1 1 3 3
5 1 1 1 1 2 3 1 3 3 1 3

b.info:

rs571227 738547
rs2827188 5597094
rs2835744 9424115

29 --ped2logicreg

This parameter is used to convert a file with PED format to LogicReg format.

The indicators supported by the parameter include (See previous specifications to find the functions of the top 3 indicators):

- --swap
- --pre
- --hwe
- --phenotype
  - Specify the phenotypes of inputted PED to a constant.
- --nor -1
  - Tell the program not to count the numbers of major and minor alleles thus to reduce run-time.
  - Used only when the source is a normalized data.
- --mode

- Specify whether to make logit transformation. 0 - No (default), 1 - Yes.

- --model
  - Specify the genetic model of the destination. 0 - additive (default), 1 - dominant, 2 - recessive.
- --out
  - Specify the output filename of LogicReg format.

Example 29: Suppose we have the files with PED format are the same as example 24.

a) Use command "coPLINK --ped2logicreg a1 --out b.txt" to generate a LogicReg file b.tx.

```
0 0 1 0 1 0 1 0 0
0 0 1 0 1 0 1 0 0
0 0 0 1 1 0 0 0 0
1 1 1 0 0 1 1 0 -
1 0 1 0 0 0 1 0 0
1 0 0 0 0 0 1 - 0
```

b) Use command "coPLINK --ped2logicreg a1 --out b.txt --swap" to generate a swapped LogicReg file b.txt.

```
0 0 1 0 1 0 1 0 0
0 0 1 0 1 0 1 0 0
0 0 0 1 1 1 1 0 0
1 1 1 0 0 0 0 0 -
1 0 1 0 0 0 1 0 0
1 0 0 0 0 0 1 - 0
```

c) Use command "coPLINK --ped2logicreg a1 --out b.txt --mode 1" to generate a logitted LogicReg file b.tx.

```
-13.8155095579638 0 1 0 1 0 1 0 0
-13.8155095579638 0 1 0 1 0 1 0 0
-13.8155095579638 0 0 1 1 0 0 0 0
+13.8155095579350 1 1 0 0 1 1 0 -
+13.8155095579350 0 1 0 0 0 1 0 0
+13.8155095579350 0 0 0 0 0 1 - 0
```

d) Use command "coPLINK --ped2logicreg a1 --out b.txt --model 2" to generate a LogicReg file b.txt with a recessive model.

```
0 0 0 0 0
0 0 0 0 0
0 0 1 0 0
```

1 1 0 1 -
1 0 0 0 0
1 0 0 0 -

e) Use command "coPLINK --ped2logicreg a1 --out b.txt --pre" to generate a new LogicReg file after preprocessing.

0 0 1 0 1 0 1
0 0 1 0 1 0 1
0 0 0 1 1 0 0
1 0 1 0 0 0 1

30 --ped2mdr

This parameter is used to convert a file with PED format to MDR format.

The indicators supported by the parameter include (See previous specifications to find the functions of the top 3 indicators):

- --swap
- --pre
- --hwe
- --phenotype
  - Specify the phenotypes of inputted PED to a constant.
- --nor -1
  - Tell the program not to count the numbers of major and minor alleles thus to reduce run-time.
  - Used only when the source is a normalized data.
- --out
  - Specify the output filename of MDR format.

Example 30: Suppose we have the files with PED format are the same as example 24.

a) Use command "coPLINK --ped2mdr a1 --out b" to generate a group of MDR files.

b.dat:

0	1	1	1	0
0	1	1	1	0
0	0	2	0	0
1	2	0	2	-1
1	1	0	1	0

```
1 0 0 1 -1
```

b.map:

```
1 rs571227 0 0
0 rs2827188 0 0
X rs2835744 0 0
Y rs181146 0 0
```

b) Use command "coPLINK --ped2mdr a1 --out b --swap" to generate a swapped MDR file b.dat.

```
0 1 1 1 0
0 1 1 1 0
0 0 2 2 0
1 2 0 0 -1
1 1 0 1 0
1 0 0 1 -1
```

c) Use command "coPLINK --ped2mdr a1 --out b --pre" to generate a new group of MDR files after preprocessing.

b.dat:

```
0 1 1 1
0 1 1 1
0 0 0 0
1 1 2 1
```

b.map:

```
1 rs571227 0 0
0 rs2827188 0 0
X rs2835744 0 0
```

31 --ped2me

This parameter is used to convert a file with PED format to ME format.

The indicators supported by the parameter include (See previous specifications to find the functions of the top 3 indicators):

- --swap
- --pre

- --hwe
- --phenotype
  - Specify the phenotypes of inputted PED to a constant.
- --nor -1
  - Tell the program not to count the numbers of major and minor alleles thus to reduce run-time.
  - Used only when the source is a normalized data.
- --out
  - Specify the output main filename of ME format.

Example 31: Suppose we have the files with PED format are the same as example 24.

a) Use command "coPLINK --ped2me a1 --out b.me" to generate a ME file b.me.

```
1 1 2 1 2 1 1 2 1 1
2 1 2 1 1 2 1 2 1 1
3 1 1 1 2 2 1 1 1 1
4 2 2 2 1 1 2 2 1 -1
5 2 1 2 1 1 2 1 1 1
6 2 1 1 1 1 2 1 -1 1
```

b) Use command "coPLINK --ped2me a1 --out b.me --swap" to generate a swapped ME file b.me.

```
1 1 2 1 2 1 2 1 1 1
2 1 2 1 1 2 2 1 1 1
3 1 1 1 2 2 2 2 1 1
4 2 2 2 1 1 1 1 1 -1
5 2 1 2 1 1 1 2 1 1
6 2 1 1 1 1 1 2 -1 1
```

c) Use command "coPLINK --ped2me a1 --out b.me --pre" to generate a new ME file after preprocessing.

```
1 1 2 1 2 1 1 2
2 1 2 1 1 2 1 2
3 1 1 1 2 2 1 1
4 2 1 2 1 1 2 1
```

32 --ped2other

Contrast to parameter --other2ped, this parameter is used to convert PED files to

any format files. The format of using this parameter is:

```
coPLINK --other2ped <in> --file2 <ped> ...
```

It is like the command lines of parameter --insert and --other2ped that the <in> is not the PED file but an arguments file. The PED file is specified by indicator --file2. And, the usage of the parameter is similar to the --insert and --other2ped too.

- The "other" files are specified in an arguments file which tells coPLINK what the components of PED files are used to generate "other" files by which formats.
- If the <in> you typed does not exist, a template file of arguments named coPLINK-2other.txt will be produced at current path/directory. The template explains the format of an argument line and the usage of the items involed in the line, and gives some examples. It looks like this:

```
# coPLINK (V4.0) by H.M. LIU et al. (Feb. 2020). Bugs report: lhmgzjx@163.com
# Arguments for parameter "--ped2other"     ← symbol '#' means current line is a comment
# Note:
# 1. Start with "#" comments a row.
# 2. Section names need be enclosed by a pair of [].
# 3. A null line will be ignored.
# 4. It represents absent if an argument line is not given.
# 5. All spaces/tabs in the argument line will be ignored, except for a delimiter.
# 6. The section names cannot be deleted or renamed, even if it is null.
# 7. To "--ped2other", the genotype matrix in PED must be stored as a whole into
#       "other" file, that is, it cannot have other rows or columns in its area, but
#       it can be transposed.
# 8. To "--ped2other", the column in the MAP must be stored as an entire row or
#       column into "other" file, that is, there cannot be other rows or columns in
#       the middle of it, but it can be turned into a row, and each column does not need
#       to be stored continuously.
# This section specifies the top 6 columns in PED and the whole 4 columns in MAP.
# Each argument line specifies a column in PED/MAP.
# Format:
#     <type>,[row/col. No.],[begin], [delimiter],[direction],[filename]
#     type - Type of a column. It may be "fam"(Family ID), "ind"(Individual ID), "pat"
#         (Paternal ID), "mat"(Maternal ID), "sex"(Sex), "phe"(Phenotype), "chr"(Chromosome),
#         "snp"(rs# or snp identifier), "gen"(Genetic distance) and "pos"(Base-pair position).
#         If the type "sex" is not given, will generate randomly.
#     row/col. No. - Row/column No. in [filename]. 0, null or a nonnumeric character represents
#         being next the row/col. from above line. A negative number means inverse order.
#     begin - Beginning position of the row/column in [filename]. Null, 0 or a negative number
#         indicates the head of the row/column.
#     delimiter - Delimiter of columns in [filename]. Null means a space/tab will be used,
```

```
#          and "\," represents a comma while "\t" is a tab (tab is used to "--ped2other").
#          Only the first character is valid when meeting more than 1.
#      direction - Direction of the data in [filename]. 0 or null means in column,
#          otherwise in row.
#      filename - Filename including the type (including the path if need). Null means
#          using the filename specified in the nearest line from above.
#      CAUTIONS:
#          a. There are 10 argument lines at most in this section. The excess will be ignored.
#          b. The <type> cannot be null.
#          c. The [filename] in the first line cannot be null.
#          d. It is case insensitive.
#          e. To "--other2ped", the null rows in [filename] will be skipped, but needs be counted
#              in argument lines.
#          f. To "--ped2other", the null rows in "other" file(s) need be counted in argument
#              lines.
#Example:
#      We have two files as follows,
#         Alleles.txt:
#         Family Individual Paternal Maternal Sex A1 A2 A1 A2 Phenotype
#         1 1 0 0 1 A A G T 1
#         <--- null line
#         2 1 0 0 1 A C T G 2
#         3 1 0 0 1 C C - G 2
#         End
#      and,
#         Snps.dat:
#         Platform,Illumina,Agilent
#         Chr,1,1
#         Rs#,snp1,snp2
#         Material,lung,musle
#         <--- null line
#         Genetic distance,0,0
#         Position,5000650,5000830
#      Then, 10 argument lines may be coded as
#         Fam,1,2, ,,alleles.txt
#         Ind,2,2
#         Pat,3,2
#         Mat,4,2
#         Sex,5,2
#         Phe,10,2
#         Chr,2,2,\,,1,snps.dat
#         Snp,3,2,\,,1
#         Gen,6,2,\,,1
#         Pos,7,2,\,,1
```

Or,
Fam,1,2, ,,alleles.txt
Ind,0,2
Pat,0,2
Mat,0,2
Sex,0,2
Phe,-1,2
Chr,2,2,\,,1,snps.dat
Snp,0,2,\,,1
Gen,6,2,\,,1
Pos,0,2,\,,1
At last, we put the 10 lines at the next line of [VECTOR BEGIN].

[VECTOR BEGIN]   ← section name, do not rename or remove it
    Fam, ← the type names of column in PED were given by the template, if the "other" files have not a column, delete or comment it
    Ind,
    Pat,
    Mat,
    Sex,
    Phe,
    Chr,
    Snp,
    Gen,
    Pos,
[VECTOR END]   ← section name, do not rename or remove it

This section specifies the rectangle area of the genotypes in the source file for PED.
Format:
<filename>,[left],[top], [delimiter],[SNP rows/col.s],[character count],[missing],[case], [ctrl],[male],[female],[AA],[Aa],[aa]
filename - Filename including the genotypes (adding the path if need). It cannot be null.
left - Left column No. of the rectangle in the source file. 1 will be used while meeting
null.
top - Top row No. of the rectangle in the source file. 1 will be used while meeting null.
delimiter - Delimiter of columns in <filename>. Null means a space, and "\," represents
a comma while "\t" is a tab(tab is used to "--ped2other").
SNP rows/col.s - Number of rows/col.s in <filename>. 0, null or out of 0~3 means
every two columns is a SNP; 1 is each column a SNP; 2 is every two rows a SNP;
and 3 is each row a SNP.
character count - Number of characters in a genotype. 1 or 2 is allowed. 0, null or
out of 1 and 2 means 2. Its value will be fixed to 2 when[SNP rows/col.s] = 0 or 2.
missing - String representing missing alleles. Null means PLINK's missing characters.
case - Character of cases. The first character will be used only. Null means using PLINK's.

```
#      ctrl - Character of controls. The first character will be used only. Null means using PLINK's.
#      male - Character of males. The first character will be used only. Null means using PLINK's.
#      female - Character of females. The first character will be used only. Null means using
#           PLINK's.
#      AA - Characters of genotype AA. When [character count] = 1, only the first character
#           is valid and null is 0. When[character count] = 2, only the first 2 characters are
#           valid and null or only one character means the two characters are the same as
#           the first character in[Aa]. The item is case sensitive.
#      Aa - Characters of genotype Aa. When [character count] = 1, only the first character is
#           valid and null is 1. When[character count] = 2, only the first 2 character are valid
#           and the first one is major allele, the other is minor allele; And, null or only one
#           character means the characters in source are used. The item is case sensitive.
#      aa - Characters of genotype aa. When [character count] = 1, only the first character
#           is valid and null is 2. When[character count] = 2, only the first 2 characters are
#           valid and null or only one character means the two characters are the same as
#           the second character in[Aa]. The item is case sensitive.
#      CAUTION:
#           a. This section cannot be null, and only the first line is valid while being more than 1.
#           b. To "--other2ped", the null rows in [filename] will be skipped, but needs be counted
#                in argument lines.
#           c. To "--ped2other", the null rows in "other" file(s) need be counted in argument
#                lines.
#
# Example:
#      Let Alleles.txt listed in section [VECTOR] as the source file, we may code like this:
#         alleles.txt,6,2, ,0,2,-,2,1,1,2
#      At last, we put the line at the next line of [MATRIX BEGIN].

[MATRIX BEGIN]      ← section name, do not rename or remove it
      ← put argument line here
[MATRIX END]        ← section name, do not rename or remove it

# This section is for "--ped2other" and "--insert", which gives the row(s)/col.(s) inserted into
#      the destination file(s).
# Format:
#      <row/col. No.>,[filename],[begin],[delimiter],[direction],[item1],[item2],...
#      row/col.No. - Position in <filename>.It cannot be null. 0, null or a nonnumeric
#           character represents being next the row/col. from above line. A negative number
#           means inverse order.    When the specified position exceeds the maximum row/col. No.,
#           the inserted text will be appended to the end.
#      filename - Filename of the inserted file. (including the path if need).Null means
#           using the filename specified in the nearest line from above (for "--ped2other")
#           or the first one (for "--insert").
#      begin - Beginning position of the row/column in "other" file(s). Null, 0 or a negative
```

```
#          number indicates the head of the row/column.
#      delimiter - Delimiter of columns. Null means a space, and "\," represents a comma
#          "\t" is a tab.
#      direction - Direction of the inserted texts in <filename>. 0 or null means in column,
#          otherwise in row.
#      item1, item2, ... - Inserted items. Their number may be or not limited, and a
#          regular expression may be used here.
#      CAUTION:
#          a. This section may be null and be allowed any number of lines.
#          b. The null lines in destination needs be counted in [begin].
#          c. The [filename] cannot be null in the first argument line, and the first one is
#              accepted only for "--insert".
#          d. For an argument line here, only ONE wildcard can be included in ONE item.
#
# Example:
#      Let Alleles.txt listed in section [VECTOR] as the "other" file, we may code like this:
#         1,alleles.txt,1, ,1,Family,Individual,Paternal,Maternal,Sex,A1,A2,A3,A4,Phenotype
#         3,,1,,1
#      Or,
#         1,alleles.txt,1,,1,Family,Individual,Paternal,Maternal,Sex,A#,Phenotype
#         3,,1,,1
#      At last, we put the lines at the next line of [INSERT BEGIN].

[INSERT BEGIN]      ← section name, do not rename or remove it
      ← put argument lines here
[INSERT END]   ← section name, do not rename or remove it
```

Notes:

- Read the specifications in the template carefully.
- The arguments lines of the components of PED files used in "other" files such as Family ID, Individual ID, and Chr.-Number need be putted between the section-names [VECTOR BEGIN] and [VECTOR END], while the arguments line of the genotypes in .ped file should be putted between [MATRIX BEGIN] and [MATRIX END]. And, if some rows will be inserted into the destination, put their arguments lines between [INSERT BEGIN] and [INSERT END].
- A line lies between the two section-names is named as an argument line.
- The null arguments lines and the spaces or TABs involed in an arguments line will be ignored by the program.
- The escape character "\," represents a comma.
- For VETOR section:
  - A repeated type name is not allowed.
  - The columns in the sources (PED files) may be saved into different "other" files which are specified by the item [filename]. The [filename]s

except the first one may be null which means using the same filename above line.

- The genotypes are considered a matrix, specified in the section MATTRIX.
  - Only one line is allowed, namely, the genotypes cannot be separated into several "other" files. If there are multiple lines in the section, only the first one is accepted.
  - Item [SNP rows/col.s] is used to specify the construct of the genotypes of a SNP in the "other" file including 0 - every two columns are a SNP, 1 - each column a SNP, 2 - every two rows a SNP and 3 - each row a SNP. Note that the value of the item may be only 1 or 3 when item [character count] was set to 1, and the case [character count] = 2 is similar.
- For section INSERT:
  - Each line indicates one row/column will be inserted into the destination file specified by the item [filename] in the line.
  - You can insert multiple rows/columns by putting multiple argument lines. The number of inserted rows/columns is not limited.
  - This section supports four wildcards to allow inserting some regular items.
    - #: Serial No. of alleles starting from 1.
    - $: Serial No. of SNPs starting from 1.
    - @: Serial No. of individuals starting from 1.
    - ^: Insert alternately the two characters following the wildcard until the end of the inserted row or column. The default values of these two characters are '0' and '1'. If the number of characters following the wildcard is less than 1, the default values will be used. For example, suppose the 'other' file has 5 columns at most. The row inserting item "A^,Class" means " A1,A2,A1,A2,Class".
    - Only one wildcard is allowed in one argument line. If there are multiple wildcards, they will be treated as ordinary characters except for the first one.
    - The escape characters of the wildcards are \#, \$, \@ and \^ respectivley.
  - It is not allowed that the number of rows of an inserted column is greater than the number of rows in the 'other' file.
- If the parameter throws any error or produces incorrect results, check the arguments file.

The indicators supported by the parameter include:

- --file2
  - Specify the main filename of PED.
- --mode
  - Specify running mode. 0 - generate the "other" files (default), 1 - list the information of PED such as the numbers of SNPs and individuals, which

may help you to design the arguments file.

- --phenotype
  - Specify the phenotypes of inputted PED to a constant.
- --out
  - Specify the main filename of log.

Example 32: Suppose we have a group of files with PED format are the same as example 24 listed here again.

a1.ped:

```
1 1 1 1 1 1 A T G C T A C C
2 1 1 1 2 1 A T C G T A C C
1 2 1 1 1 1 T T G G T T C C
1 2 2 2 2 2 A A C C A A C N
5 1 1 1 1 2 T A C C A T C C
6 1 1 1 1 2 T T C C A T - C
```

a1.map:

```
1 rs571227  0 738547
0 rs2827188 0 5597094
X rs2835744 0 9424115
Y rs181146  0 1387981
```

a) Suppose we want to convert the PED files to a group GEO files named as b.geo and b.map by using the parameter. We design the arguments file (named as a.txt) firstly:

```
[VECTOR BEGIN]  ← <type>,[row/col. No.],[begin], [delimiter],[direction],[filename]
phe,    1,    1,    \,,    1,    b.geo
pos,    1,    2,    \,,    ,     b.map
gen,    ,     2,    \,    ← two null items mean the column No. is next type POS and
        filename the same as above
chr,    ,     2,    \,
snp,    ,     2,    \,
 [VECTOR END]
 [MATRIX BEGIN]       ← <filename>,[left],[top], [delimiter], [SNP rows/col.s], [character
                      count],[missing],[case],[ctrl],[male], [female],[AA],[Aa],[aa]
b.geo,1,2, \,,3,1,-1,1,0,,,2,0,1
[MATRIX END]
[INSERT BEGIN]  ← <row/col. No.>,[filename],[begin],[delimiter],[direction],[item1],,...
1,b.map,1,\,,1,Affymetrix ID,Target Description,Gene Title,Gene Symbol
[INSERT END]
```

b) Use command "coPLINK --ped2other a.txt --file2 a1 --out b" to generate two files with GEO format.

b.geo:

```
0,0,0, 1,1, 1
0,0,2, 1,0, 2
0,0,1, 2,2, 2
0,0,2, 1,0, 0
2,2,2,-1,2,-1
```

b.map:

Affymetrix ID,	Target Description,	Gene Title,	Gene Symbol
738547,	0,	1,	rs571227
5597094,	0,	0,	rs2827188
9424115,	0,	X,	rs2835744
1387981,	0,	Y,	rs181146

Compare to example 26, the results above are the same.

c) Some examples of arguments file.

Ped to tped:

```
[VECTOR BEGIN]
    Fam,1,,,,1.tfam
    Ind,0
    Pat,0
    Mat,0
    Sex,0
    Phe,0
    Chr,1,,,,1.tped
    Snp,0
    Gen,0
    Pos,0
[VECTOR END]
[MATRIX BEGIN]
1.tped,5,1, ,3,2
[MATRIX END]
[INSERT BEGIN]

[INSERT END]
```

Ped to boost:

```
[VECTOR BEGIN]
Phe,1,1,,,1.txt
[VECTOR END]
[MATRIX BEGIN]
1.txt,2,1, ,1,1,-1,1,0,,,0,1,2
[MATRIX END]
[INSERT BEGIN]

[INSERT END]
```

Ped to beam:

```
[VECTOR BEGIN]
Snp,1,2,\t,,1.txt
Chr,0,2,\t
Pos,0,2,\t
phe,1,4,,1
[VECTOR END]
[MATRIX BEGIN]
1.txt,4,2, ,3,1,-,1,0,,,2,0,1
[MATRIX END]
[INSERT BEGIN]
1,1.txt,1,\t,1,ID,Chr,Pos
[INSERT END]
```

Ped to mdr:

```
[VECTOR BEGIN]
phe,1,1,\t,,1.dat
chr,1,1,\t,,1.map
snp,,1,\t
pos,,1,\t
[VECTOR END]
[MATRIX BEGIN]
1.dat,2,1, \t,1,1,-1,1,0,,,0,1,2
[MATRIX END]
[INSERT BEGIN]

[INSERT END]
```

33 --ped2ped

This parameter is not a converting parameter. It is mainly used to preprocess PED

files or to generate sexes randomly for the files (PLINK does not analyze a file without sexes sometimes).

The indicators supported by the parameter include (See previous specifications to find the functions of the top 6 indicators):

- --pre
- --rnd-sex
- --swap
- --seed
- --hwe
- --nor
- --phenotype
  - Specify the phenotypes of inputted PED to a constant.
- --mode
  - Specify running mode. 0 - save destinations (default); 1 - does not save destinations but lists results after preprocessing.
  - If the indicator --rnd-sex, --swap or --nor is used, the destinations will be saved regardless of its value.
- --out
  - Specify the output main filename of PED format.

Example 33: Suppose we have the files with PED format are the same as example 24.

a) Use command "coPLINK --ped2ped a1 --out b --rnd-sex" to generate sexes randomly for a1.

```
1 1 1 1 2 1 A T G C T A C C
2 1 1 1 1 1 A T C G T A C C
1 2 1 1 1 1 T T G G T T C C
1 2 2 2 2 2 A A C C A A C N
5 1 1 1 1 2 T A C C A T C C
6 1 1 1 2 2 T T C C A T N C
```

b) Command "coPLINK --ped2ped a1 --out b --pre --mode 1" lists the results after preprocessing.

```
...
There are 6 sample(s) before alleles missing rates of samples checking.
    Sample 4 failed in alleles missing rate checking.
    Sample 5 failed in alleles missing rate checking.
There are 4 sample(s) after alleles missing rates of samples checking.
It is total 4 SNPs before HWE and MAF test.
    SNP 4 failed in MAF and HWE testing.
    100% completed.
```

```
It is total 3 SNPs after HWE and MAF test.
...
```

34 --ped2pretty

This parameter converts a group of PED files to a file with prettybase format. A prettybase file is formatted in plain-text with TAB delimiter, which includes two formats:

- Slider version. This format is used in tool Slider (http://genapps.uchicago.edu/slider/). Each row of the file has the following formats: site position, individual ID, first allele and second allele. A polymorphism data file of this format with N individuals and M sites has the following format:

```
pos_1     dip_id_1     allele1a_1     allele1b_1
pos_1     dip_id_2     allele2a_1     allele2b_1
pos_1     dip_id_3     allele3a_1     allele3b_1
.         .            .              .
.         .            .              .
.         .            .              .
pos_1     dip_id_N     alleleNa_1     alleleNb_1
pos_2     dip_id_1     allele1a_2     allele1b_2
pos_2     dip_id_2     allele2a_2     allele2b_2
pos_2     dip_id_3     allele3a_2     allele3b_2
.         .            .              .
.         .            .              .
.         .            .              .
pos_2     dip_id_N     alleleNa_2     alleleNb_2
.         .            .              .
.         .            .              .
.         .            .              .
pos_M     dip_id_N     alleleNa_M     alleleNb_M
```

  - TAB delimited.
  - Using 'N' or '?' represents a missing allele.

- SeattleSNPs version. This format describes all SNP sites discovered by the SeattleSNPs PGA (https://pga.gs.washington.edu/). The row format of this file is:

```
<chromosome position-HUGO_NAME-chromosome> <PGA Sample ID> <Allele1> <Allele2>

Example: 74772592-PLAU-10     D001     G     T
```

- The 'chromosome position' is generated from mapping to the most recent genome assembly available from the UCSC Genome Assembly.
- TAB delimited.
- An allele may be several letters, and the first letter is used in this case.
- Character '-' or 'N' is used to indicate an allele.

The parameter supports all the three missing characters in the two versions. The indicators supported by the parameter include (See previous specifications to find the functions of the top 4 indicators):

- --nor
- --pre
- --hwe
- --swap
- --mode
  - Specify the version of the destination. 0 - Slider version (default); 1 - SeattleSNPs version.
- --memo
  - Specify HUGO_NAME for the old version only. Default "NO_NAME".
- --out
  - Specify the output filename of prettybase format.

Example 34: Suppose we have the files with PED format are the same as example 24.

a) Use command "coPLINK --ped2pretty a1 --out b" to produce a Slider version prettybase file b.pretty.

```
738547	1	A	T
738547	1	A	T
738547	2	T	T
738547	2	A	A
738547	1	T	A
738547	1	T	T
5597094	1	G	C
5597094	1	C	G
5597094	2	G	G
5597094	2	C	C
5597094	1	C	C
5597094	1	C	C
9424115	1	T	A
9424115	1	T	A
9424115	2	T	T
9424115	2	A	A
9424115	1	A	T
9424115	1	A	T
```

1387981	1	C	C
1387981	1	C	C
1387981	2	C	C
1387981	2	C	N
1387981	1	C	C
1387981	1	N	C

b) Change the command into "coPLINK --ped2pretty a1 --out b --swap" to produce a Slider version swapped prettybase file b.pretty.

738547	1	A	T
738547	1	A	T
738547	2	T	T
738547	2	A	A
738547	1	T	A
738547	1	T	T
5597094	1	G	C
5597094	1	C	G
5597094	2	G	G
5597094	2	C	C
5597094	1	C	C
5597094	1	C	C
9424115	1	A	T
9424115	1	A	T
9424115	2	A	A
9424115	2	T	T
9424115	1	T	A
9424115	1	T	A
1387981	1	C	C
1387981	1	C	C
1387981	2	C	C
1387981	2	C	N
1387981	1	C	C
1387981	1	N	C

c) Use command "coPLINK --ped2pretty a1 --out b --pre" to generate a new prettybase file of Slider version after preprocessing.

738547	1	A	T
738547	1	A	T
738547	2	T	T
738547	1	T	A
5597094	1	G	C
5597094	1	C	G
5597094	2	G	G

```
5597094   1   C   C
9424115   1   T   A
9424115   1   T   A
9424115   2   T   T
9424115   1   A   T
```

d) Use command "coPLINK --ped2pretty a1 --out b --mode 1 --memo PLAU" to produce a SeattleSNPs version prettybase file b.pretty with specified HUGO_NAME as "PLAU".

```
738547-PLAU-1   1   A   T
738547-PLAU-1   1   A   T
738547-PLAU-1   2   T   T
738547-PLAU-1   2   A   A
738547-PLAU-1   1   T   A
738547-PLAU-1   1   T   T
5597094-PLAU-0 1   G   C
5597094-PLAU-0 1   C   G
5597094-PLAU-0 2   G   G
5597094-PLAU-0 2   C   C
5597094-PLAU-0 1   C   C
5597094-PLAU-0 1   C   C
9424115-PLAU-X 1   T   A
9424115-PLAU-X 1   T   A
9424115-PLAU-X 2   T   T
9424115-PLAU-X 2   A   A
9424115-PLAU-X 1   A   T
9424115-PLAU-X 1   A   T
1387981-PLAU-Y 1   C   C
1387981-PLAU-Y 1   C   C
1387981-PLAU-Y 2   C   C
1387981-PLAU-Y 2   C   N
1387981-PLAU-Y 1   C   C
1387981-PLAU-Y 1   N   C
```

35 --ped2svmsnp

This parameter converts a group of PED files to a group of files with SVMSNPs format. SVMSNPs format is a format for the GWAS software SVMSNPs. It includes two or three files:

- Data file (with extension .dat here). Each row is an individual and each column is a SNP. The program defines '-1' as a missing allele or genotype.

The genotypes can be (1) encoded by simply converting each SNP A/B into the number of copies of the major allele (here names as Major counting), or (2) encoded as follows:

- If the SNP is A/B, where A and B denote nucleotides in sorted order, then the encoded genotype of AA is 0, AB or BA is 1, and BB is 2.
- For example if we have two cases (or controls) genotypes for 3 SNPs A/C, C/G, and C/T, then the encoding would be

```
AA CC CT => 0 0 1
AC GG CC => 1 2 0
```

- We name the encoding method as Letter order.

- Label file (with extension .lab here). Each row is corresponding to an individual in data file, which contains two integers. The first one is the phenotypes using 0 to denote case and 1 to denote control, and second the row number encoding from 0. Note that the statement of the two integers on web site "http://svmsnps.njit.edu/help.html" of SVMSNPs is in inverse which is different from the example data on the web. Here, the definition of the example is used.
- Optional SNP names (with extension .snp here). Each row is a SNP corresponding to the SNP in data file as represented in column. Only one column in the file.

All files are space delimited.

The indicators supported by the parameter include (See previous specifications to find the functions of the top 3 indicators):

- --nor
- --pre
- --hwe
- --phenotype
  - Specify the phenotypes of inputted PED to a constant.
- --mode
  - Specify the encoding method of genotypes. 0 - Letter order (default); 1 - Major counting.
- --out
  - Specify the output main filename of SVMSNPs format.

Example 35: Suppose we have the files with PED format are the same as example 24.

a) Use command "coPLINK --ped2svmsnp a1 --out b" to produce a group of SVMSNPs files with encoding Letter order.

b.dat:

1 1 1 2
1 1 1 2
2 2 2 2
0 0 0 -1
1 0 1 2
2 0 1 -1

b.lab:

1 0
1 1
1 2
0 3
0 4
0 5

b.snp:

rs571227
rs2827188
rs2835744
rs181146

b) Change the command into "coPLINK --ped2svmsnp a1 --out b --mode 1" to generate a new data file encoding Major counting.

1 1 1 2
1 1 1 2
2 0 0 2
0 2 2 -1
1 2 1 2
2 2 1 -1

c) Use command "coPLINK --ped2svmsnp a1 --out b --pre" to produce a group of files after preprocessing.

b.dat:

1 1 1
1 1 1
2 2 2
1 0 1

b.lab:

1 0
1 1
1 2
0 3

b.snp:

rs571227
rs2827188
rs2835744

36 --ped2tped

This parameter converts a group of PED files to a group of files with TPED format. A TPED format is a transposed format of PED, which is explained in details in PLINK document. The parameter is different from PLINK's --transpose in that it does not change the SNP's order of the source, and in that it supports two formats of TPED which one is standard format as described in PLINK document and the other is a user-defined format as described below:

- Use 0, 1 and 2 to represent genotypes AA, Aa and aa respectively. And, "-1" denotes a missing genotype.
- The other definitions are the same as the standard format.

The indicators supported by the parameter include (See previous specifications to find the functions of the top 4 indicators):

- --swap
- --nor
- --pre
- --hwe
- --phenotype
  - Specify the phenotypes of inputted PED to a constant.
- --mode
  - Specify the output format. 0 - standard (default); 1 - user-defined.
  - The genotypes will be normalized by force when the mode is specified to 1. If the PED files are normalized, the indicator '--nor -1' may be used to not normalize the genotypes to reduce run-time.
- --out
  - Specify the output main filename of TPED format.

Example 36: Suppose we have the files with PED format are the same as example 24.

a) Use command "coPLINK --ped2tped a1 --out b" to produce a group of TPED files with standard format.

b.tped:

```
1 rs571227  0 738547  A T A T T T A A T A T T
0 rs2827188 0 5597094 G C C G G G C C C C C C
X rs2835744 0 9424115 T A T A T T A A A T A T
Y rs181146  0 1387981 C C C C C C C N C C N C
```

b.tfam:

```
1 1 1 1 1 1
2 1 1 1 2 1
1 2 1 1 1 1
1 2 2 2 2 2
5 1 1 1 1 2
6 1 1 1 1 2
```

b) Change the command into "coPLINK --ped2tped a1 --out b --mode 1" to generate a new data file with user-defined format.

```
1 rs571227  0 738547  1 1 0  2 1  0
0 rs2827188 0 5597094 1 1 2  0 0  0
X rs2835744 0 9424115 1 1 0  2 1  1
Y rs181146  0 1387981 0 0 0 -1 0 -1
```

c) Use command "coPLINK --ped2tped a1 --out b --pre" to produce a group of files after preprocessing.

b.tped:

```
1 rs571227  0 738547  A T A T T T T A
0 rs2827188 0 5597094 G C C G G G C C
X rs2835744 0 9424115 T A T A T T A T
```

b.tfam:

```
1 1 1 1 1 1
2 1 1 1 2 1
1 2 1 1 1 1
5 1 1 1 1 2
```

37 --pretty2ped

This parameter converts a prettybase file to a group of files with PED format. A prettybase format file (1) is the version of either Slider or SeattleSNPs; (2) may have the same site position in different SNPs; (3) may have the same individual ID in one SNP (e.g., a prettybase file converted from PED files because of that PED format has a family ID and an individual ID leading to the same individual ID in different family IDs). We can identify the SNPs based on the individual IDs for the case (2), and based on the site positions for the case (3). The parameter can automatically recognize the version of a prettybase file for the case (1), and uses --mode indicator to determine the case (2) or (3).

The parameter supports all the three missing characters in the two versions, '-', 'N' and '?'. The indicators supported by the parameter include (See previous specifications to find the functions of the first indicator):

- --seed
- --phenotype
  - Specify the phenotype of destination. Default "1".
- --chr-no
  - Specify the chromosome No. in the destination with Slider version only.
  - Default "0".
- --mode
  - Specify which identifying mode to use. 0 - based on individual IDs (default); 1 - based on site positions.
- --out
  - Specify the output main filename of PED format.

Example 37: Suppose we have a file a.pretty with Slider version format, as listed below:

```
738547    1  A  T
738547    2  A  T
738547    3  T  T
738547    4  A  A
738547    5  T  A
738547    6  T  T
5597094   1  G  C
5597094   2  C  G
5597094   3  G  G
5597094   4  C  C
5597094   5  C  C
5597094   6  C  C
5597015   1  T  A
```

5597094	2	T	A
5597015	3	T	T
5597094	4	A	A
5597094	5	A	T
5597094	6	A	T
1387981	1	C	C
1387981	2	C	C
1387981	3	C	C
1387981	4	C	N
1387981	5	C	C
1387981	6	N	C

a) Use command "coPLINK --pretty2ped a.pretty --out b" to produce a group of PED files with main filename b.

b.ped:

```
1 1 0 0 2 1 A T G C T A C C
1 2 0 0 1 1 A T C G T A C C
1 3 0 0 1 1 T T G G T T C C
1 4 0 0 1 1 A A C C A A C N
1 5 0 0 2 1 T A C C A T C C
1 6 0 0 2 1 T T C C A T N C
```

b.map:

```
0 SNP1 0 738547
0 SNP2 0 5597094
0 SNP3 0 5597015
0 SNP4 0 1387981
```

b) Change the command to "coPLINK --pretty2ped a.pretty --out b --chr-no X --phenotype 2" to produce a new group of PED files with main filename b.

b.ped:

```
1 1 0 0 2 2 A T G C T A C C
1 2 0 0 1 2 A T C G T A C C
1 3 0 0 1 2 T T G G T T C C
1 4 0 0 2 2 A A C C A A C N
1 5 0 0 1 2 T A C C A T C C
1 6 0 0 2 2 T T C C A T N C
```

b.map:

```
X SNP1 0 738547
X SNP2 0 5597094
X SNP3 0 5597015
X SNP4 0 1387981
```

c) If we change the command to "coPLINK --pretty2ped a.pretty --out b --mode 1" further, the program will throw error information as follow and abort.

```
Error: The numbers of SNPs and samples do not match the number of alleles (3*6 != 13).
```

d) Suppose another prettybase file a1.pretty as listed below need be converted to PED. Use command "coPLINK --pretty2ped a1.pretty --out b --mode 1" to reach the target.

```
738547-PLAU-1   1   A   T
738547-PLAU-1   1   A   T
738547-PLAU-1   2   T   T
738547-PLAU-1   2   A   A
738547-PLAU-1   1   T   A
738547-PLAU-1   1   T   T
5597094-PLAU-0 1   G   C
5597094-PLAU-0 1   C   G
5597094-PLAU-0 2   G   G
5597094-PLAU-0 2   C   C
5597094-PLAU-0 1   C   C
5597094-PLAU-0 1   C   C
9424115-PLAU-X 1   T   A
9424115-PLAU-X 1   T   A
9424115-PLAU-X 2   T   T
9424115-PLAU-X 2   A   A
9424115-PLAU-X 1   A   T
9424115-PLAU-X 1   A   T
1387981-PLAU-Y 1   C   C
1387981-PLAU-Y 1   C   C
1387981-PLAU-Y 2   C   C
1387981-PLAU-Y 2   C   N
1387981-PLAU-Y 1   C   C
1387981-PLAU-Y 1   N   C
```

The results:

b.ped:

```
1 1 0 0 1 1 A T G C T A C C
1 1 0 0 1 1 A T C G T A C C
```

```
1 2 0 0 2 1 T T G G T T C C
1 2 0 0 1 1 A A C C A A C N
1 1 0 0 2 1 T A C C A T C C
1 1 0 0 2 1 T T C C A T N C
```

b.map:

```
1 SNP1 0 738547
0 SNP2 0 5597094
X SNP3 0 9424115
Y SNP4 0 1387981
```

The results show the parameter recognizes the version of the source correctly.

e) Suppose an additional prettybase file a2.pretty as listed below need be converted to PED. Use command "coPLINK --pretty2ped a2.pretty --out b --mode 1" to reach the target.

```
738547    1   A   T
738547    1   A   T
738547    2   T   T
738547    2   A   A
738547    1   T   A
738547    1   T   T
5597094   1   G   C
5597094   1   C   G
5597094   2   G   G
5597094   2   C   C
5597094   1   C   C
5597094   1   C   C
9424115   1   T   A
9424115   1   T   A
9424115   2   T   T
9424115   2   A   A
9424115   1   A   T
9424115   1   A   T
1387981   1   C   C
1387981   1   C   C
1387981   2   C   C
1387981   2   C   N
1387981   1   C   C
1387981   1   N   C
```

The results:

b.ped:

```
1 1 0 0 2 1 A T G C T A C C
1 1 0 0 1 1 A T C G T A C C
1 2 0 0 1 1 T T G G T T C C
1 2 0 0 1 1 A A C C A A C N
1 1 0 0 1 1 T A C C A T C C
1 1 0 0 1 1 T T C C A T N C
```

b.map:

```
0 SNP1 0 738547
0 SNP2 0 5597094
0 SNP3 0 9424115
0 SNP4 0 1387981
```

The results tell a correct command line.

f) If a command "coPLINK --pretty2ped a2.pretty --out b" is used, an incorrect result will be produced.

b.ped:

```
1 1 0 0 2 1 A T A T
```

b.map:

```
0 SNP1 0 738547
0 SNP2 0 738547
```

38 --space2csv

This parameter converts a space delimited file to a comma delimited file.

The parameter supports indicator --out only:

- --out
  - Specify the main filename of output delimited by a comma.

Note: the parameter considers consecutive spaces as one space.

Example 38: Suppose we have a space delimited file a.txt with consecutive spaces, as listed below:

```
Y SNP1   0  1387981
0 SNP2 0 5597094
1 SNP3 0   738547
X SNP4 0    9424115
```

a) Command "coPLINK --space2csv a.txt --out b.csv" produces a comma delimited file b.csv.

```
Y,SNP1,0,1387981
0,SNP2,0,5597094
1,SNP3,0,738547
X,SNP4,0,9424115
```

Open b.csv by Excel:

	A	B	C	D
1	Y	SNP1	0	1387981
2	0	SNP2	0	5597094
3	1	SNP3	0	738547
4	X	SNP4	0	9424115
5				

39 --splice-ped

This parameter splices the individuals of two PED files. Its command line looks like this:

```
coPLINK --splice-ped <in> --file2 <file2> [indicator1 [value1]] ...
```

Here, we call <in> as file1. The parameter appends the individuals of file2 to file1 based on the SNPs of file1. If a SNP of file1 does not exist in file2, the SNP's genotypes of the individual in file2 will be encoded as missing. It is worth pointing out that the parameter can align the SNPs of file2 with that of file1.

The indicators supported by the parameter include (See previous specifications to find the functions of the first indicator):

- --nor
- --phenotype
  - Specify a phenotype to replace the phenotypes of file2. Default "0".
  - If the indicator is absent, file2 will reserve the phenotypes of itself.
- --file2
  - Specify the splicing file file2.

- --out
    - Specify the output main filename of PED format.

Example 39: Suppose we have a group of PED files with main filename "a1" are the same as example 24, and a group of PED files with main filename "b" having the same .map file with a1.map. The b.* are listed below. Compare with example 24, the order of SNP3 and SNP4 is swapped.

b.ped:

```
1 1 0 0 1 1 A T G C T A G C
1 2 0 0 2 1 A A C G T A C G
1 3 0 0 2 1 T T G G T T G G
1 4 0 0 2 1 T A C C A A C N
```

b.map:

```
1 rs571227  0 738547
0 rs2827188 0 5597094
Y rs181146  0 1387981
X rs2835744 0 9424115
```

a) Use command "coPLINK --splice-ped a1 --file2 b --out c" to produce a group of PED files with main filename c.

c.ped:

```
1 1 1 1 1 1 A T G C T A C C
2 1 1 1 2 1 A T C G T A C C
1 2 1 1 1 1 T T G G T T C C
1 2 2 2 2 2 A A C C A A C N
5 1 1 1 1 2 T A C C A T C C
6 1 1 1 1 2 T T C C A T - C
1 1 0 0 1 1 A T G C G C T A
1 2 0 0 2 1 A A C G C G T A
1 3 0 0 2 1 T T G G G G T T
1 4 0 0 2 1 T A C C C N A A
```

c.map:

```
1 rs571227  0 738547
0 rs2827188 0 5597094
X rs2835744 0 9424115
Y rs181146  0 1387981
```

From the results, we can learn the data of b.ped was appended to a.ped and the phenotypes of b.ped were reserved. Furthermore, the SNPs of b were aligned to a.

b) Change the command into "coPLINK --splice-ped a1 --file2 b --out c --nor --phenotype 2" to produce a normalized PED file. The phenotypes of b will be set to "2".

```
1 1 1 1 1 1 d D d D D d D D
2 1 1 1 2 1 d D D d D d D D
1 2 1 1 1 1 D D d d D D D D
1 2 2 2 2 2 d d D D d d D N
5 1 1 1 1 2 D d D D d D D D
6 1 1 1 1 2 D D D D d D N D
1 1 0 0 1 2 D d D d D d D d
1 2 0 0 2 2 D D d D d D D d
1 3 0 0 2 2 d d D D D D D D
1 4 0 0 2 2 d D d d d N d d
```

c) Suppose another group of PED files "b1" as listed below need be appended to a.ped. Compared to a, the files lost the second SNP. Use command "coPLINK --splice-ped a1 --file2 b1 --out c" to reach the target.

b1.ped:

```
1 1 0 0 1 1 A T T A G C
1 2 0 0 2 1 A A T A C G
1 3 0 0 2 1 T T T T G G
1 4 0 0 2 1 T A A A C N
```

b1.map:

```
1 rs571227  0 738547
X rs2835744 0 9424115
Y rs181146  0 1387981
```

The results:

c.ped:

```
1 1 1 1 1 1 A T G C T A C C
2 1 1 1 2 1 A T C G T A C C
1 2 1 1 1 1 T T G G T T C C
1 2 2 2 2 2 A A C C A A C N
5 1 1 1 1 2 T A C C A T C C
6 1 1 1 1 2 T T C C A T - C
```

```
1 1 0 0 1 1 A T N N T A G C
1 2 0 0 2 1 A A N N T A C G
1 3 0 0 2 1 T T N N T T G G
1 4 0 0 2 1 T A N N A A C N
```

c.map:

```
1 rs571227  0 738547
0 rs2827188 0 5597094
X rs2835744 0 9424115
Y rs181146  0 1387981
```

Compare with the results of step a, here the genotypes of the first half of c.ped is the same as that of step a, but the genotypes of the second SNP of the second half of c.ped were set to missing since the SNP is missing in b1.

40 --svmsnp2ped

This parameter converts a group of SVMSNPs files to a group of PED files.

The indicators supported by the parameter include (See previous specifications to find the functions of the top 2 indicators):

- --seed
- --chr-no
- --mode
  - Specify the encoding method of genotypes. 0 - Letter order (default); 1 - Major counting.
- --out
  - Specify the output main filename of PED format.

Example 40: Suppose we have the files with SVMSNPs format as follows:

a.dat:

```
1 1 1 2
1 1 1 2
2 2 2 2
0 0 0 -1
1 0 1 2
2 0 1 -1
```

a.lab:

```
1 0
1 1
1 2
0 3
0 4
0 5
```

a.snp:

```
rs571227
rs2827188
rs2835744
rs181146
```

a) Use command "coPLINK --svmsnp2ped a --out b" to produce a group of PED files by using encoding Letter order.

b.ped:

```
1 0 0 0 2 1 d D d D d D d d
1 1 0 0 2 1 d D d D d D d d
1 2 0 0 1 1 d d d d d d d d
1 3 0 0 2 2 D D D D D D N N
1 4 0 0 2 2 d D D D d D d d
1 5 0 0 1 2 d d D D d D N N
```

b.map:

```
0 rs571227  0 0
0 rs2827188 0 0
0 rs2835744 0 0
0 rs181146  0 0
```

b) Change the command into "coPLINK --svmsnp2ped a --out b --mode 1 --chr-no Y" to generate a new data file by using encoding Major counting and set the chromosome No. to Y.

b.ped:

```
1 0 0 0 1 1 d D d D d D D D
1 1 0 0 2 1 d D d D d D D D
1 2 0 0 1 1 D D D D D D D D
1 3 0 0 1 2 d d d d d d N N
1 4 0 0 2 2 d D d d d D D D
1 5 0 0 1 2 D D d d d D N N
```

b.map:

```
Y rs571227  0 0
Y rs2827188 0 0
Y rs2835744 0 0
Y rs181146  0 0
```

41 --test-allele

This parameter is used to test the composition of the genotypes of the PED files in a specified folder. Its command line looks like this:

```
coPLINK --test-allele <in> [indicator1 [value1]] ...
```

Here, <in> is the tested folder.

The indicators supported by the parameter include (See previous specifications to find the functions of the first indicator):

- --nor
- --mode
  - Specify the test mode.
    - 0 - Heterozygote Only (default). It tests the composition of the heterozygotes only.
    - 1 - All genotypes. This mode tests not only the composition of the heterozygotes but also that of the homozygotes.
- --out
  - Specify the output main filename of testing results.

Example 41: Suppose we want to test the folder "a" which includes two group of PED files as follows:

test.ped:

```
1 1 0 0 1 1 A C T G C A C C
2 1 0 0 1 1 A C T G C A C C
3 1 0 0 1 1 C C G G C C C C
4 1 0 0 1 2 A A T T A A C C
5 1 0 0 1 2 C A T T A C C C
6 1 0 0 1 2 C C T T A C C C
```

test.map:

```
1 snp1 0 1
```

```
1 snp2 0 2
1 snp3 0 3
1 snp4 0 4
```

tt.ped:

```
1 1 0 0 1 1 A A G T
1 1 0 0 2 2 A C T G
1 1 0 0 1 1 C C N G
```

tt.map:

```
1 snp1 0 5000650
1 snp2 0 5000830
```

a) Use command "coPLINK --test-allele a --out b" to test the composition of the heterozygotes in folder "a" and produce result file b.csv.

```
File Name,  SNP,   Genotype,    Test mode          ← tittle
a\test.ped, snp1,  AC|CA,       Heterozygote Only  ← two types of heterozygotes
a\test.ped, snp2,  TG,          Heterozygote Only
a\test.ped, snp3,  AC|CA,       Heterozygote Only
a\tt.ped,   snp1,  AC,          Heterozygote Only
a\tt.ped,   snp2,  GT|NG|TG,    Heterozygote Only
```

The results suggest the snp4 in test.ped has only homozygotes.

b) Change the command to "coPLINK --test-allele a --out b --mode 1" to test the composition of all types of the genotypes. Open the result file with Excel.

File Name	SNP	Genotype	Test mode
a\test.ped	snp1	AA|AC|CA|CC	All genotypes
a\test.ped	snp2	GG|TG|TT	All genotypes
a\test.ped	snp3	AA|AC|CA|CC	All genotypes
a\test.ped	snp4	CC	All genotypes
a\tt.ped	snp1	AA|AC|CC	All genotypes
a\tt.ped	snp2	GT|NG|TG	All genotypes

The results above tell us that the genotypes of snp2 in tt.ped have only heterozygotes.

c) Change the command to "coPLINK --test-allele a --out b --mode 1 --nor" to test the composition of all types of normalized genotypes. Open the result file with Excel.

File Name	SNP	Genotype	Test mode
a\test.ped	snp1	DD|Dd|dD|dd	All genotypes
a\test.ped	snp2	DD|Dd|dd	All genotypes
a\test.ped	snp3	DD|Dd|dD|dd	All genotypes
a\test.ped	snp4	DD	All genotypes
a\tt.ped	snp1	DD|Dd|dd	All genotypes
a\tt.ped	snp2	Dd|ND|dD	All genotypes

42 --tped2bed

This parameter is used to convert a group of TPED files to PLINK BED format. The file-set of TPED format in PLINK has no an optional file TSNP but some platform (such as WTCCC) has. A TSNP file records the information of SNPs, which includes several rows and one SNP per row. E.g.,

```
1   15385344    EGAV00000562210    SNP_A-1938722    rs761296
1   243847383   EGAV00007281350    SNP_A-4217222    rs11582843
    ...
```

The file is a TAB delimited plain-text file.

- Column 1: chromosome No.
- Column 2: base-pair position
- Column 3: EGAV No.
- Column 4: SNP label
- Column 5: RS#

The program does not care about the first three columns. And, it will check whether there is a .tsnp file in the path of source files. If it is, it gets RS#s from the file, otherwise takes them from the .tped file.

The indicators supported by the parameter include (See previous specifications to find the functions of the top 2 indicators):

- --pre
- --hwe
- --nor -1
  - Tell the program not to count the numbers of major and minor alleles thus to reduce run-time.
  - Used only when the source is a normalized data such as a TPED data converted from a normalized PED data.
- --phenotype
  - Specify the phenotype of .fam file.
- --out

- Specify the output main filename of BED format.

Example 42: Suppose we have a file-set of TPED format as listed below.

a.tped:

```
1 EGAV1 0 738547   A T A T T T A A T A T T
0 EGAV2 0 5597094  G C C G G G C C C C C C
X EGAV3 0 9424115  T A T A T T A A A T A T
Y EGAV4 0 1387981  C C C C C C C N C C N C
```

a.tfam:

```
1 1 1 1 1 1
2 1 1 1 2 1
1 2 1 1 1 1
1 2 2 2 2 2
5 1 1 1 1 2
6 1 1 1 1 2
```

a.tsnp:

```
1 738547    EGAV1  rs571227  0
Y 1387981   EGAV4  rs181146  0
0 5597094   EGAV2  rs2827188 0
X 9424115   EGAV3  rs2835744 0
```

a) Use command "coPLINK --tped2bed a --out b" to generate a group of BED files with main filename b.

b.bed:

```
6C 1B 01 3A 0E CA 0F 3A 0A 7F 07
```

b.bim:

```
1 rs571227   0  738547  A  T
0 rs2827188  0  5597094 G  C
X rs2835744  0  9424115 A  T
Y rs181146   0  1387981 N  C
```

b.fam:

```
1 1 1 1 1 1
2 1 1 1 2 1
1 2 1 1 1 1
```

1 2 2 2 2 2
5 1 1 1 1 2
6 1 1 1 1 2

b) Use command "coPLINK --tped2bed a --out b --pre 0.2" to re-generate BED files with main filename b.

b.bed:

6C 1B 01 3A 0E CA 0F CA 0A

b.bim:

1 rs571227 0 738547 A T
0 rs2827188 0 5597094 G C
X rs2835744 0 9424115 A T

b.fam:

1 1 1 1 1 1
2 1 1 1 2 1
1 2 1 1 1 1
1 2 2 2 2 2
5 1 1 1 1 2
6 1 1 1 1 2

c) Remove a.tsnp, and type command "coPLINK --tped2bed a --out b" to re-generate BED files with main filename b.

b.bed:

6C 1B 01 3A 0E CA 0F 3A 0A 7F 07

b.bim:

1 EGAV1 0 738547 A T
0 EGAV2 0 5597094 G C
X EGAV3 0 9424115 A T
Y EGAV4 0 1387981 N C

b.fam:

1 1 1 1 1 1
2 1 1 1 2 1
1 2 1 1 1 1
1 2 2 2 2 2

```
5 1 1 1 1 2
6 1 1 1 1 2
```

43 --tped2ped

This parameter is used to convert a group of TPED files to PLINK PED format.

Like --tped2bed, the parameter does not care about the first three columns in a .tnsp file too. And, it will check whether there is a .tsnp file in the path of source files. If it is, it gets RS#s from the file, otherwise takes them from the .tped file.

The indicators supported by the parameter include (See previous specifications to find the functions of the top 6 indicators):

- --pre
- --nor
- --rnd-sex
- --seed
- --hwe
- --phenotype
- --out
    - Specify the output main filename of PED format.

Example 43: Suppose we have a file-set of TPED format are the same as example 42.

a) Use command "coPLINK --tped2ped a --out b" to generate a group of PED files with main filename b.

b.ped:

```
1 1 1 1 1 1 A T G C T A C C
2 1 1 1 2 1 A T C G T A C C
1 2 1 1 1 1 T T G G T T C C
1 2 2 2 2 2 A A C C A A C N
5 1 1 1 1 2 T A C C A T C C
6 1 1 1 1 2 T T C C A T N C
```

b.map:

```
1 rs571227  0 738547
0 rs2827188 0 5597094
X rs2835744 0 9424115
Y rs181146  0 1387981
```

b) Use command "coPLINK --tped2ped a --out b --pre 0.2" to re-generate PED files with main filename b.

b.ped:

```
1 1 1 1 1 1 A T G C T A
2 1 1 1 2 1 A T C G T A
1 2 1 1 1 1 T T G G T T
1 2 2 2 2 2 A A C C A A
5 1 1 1 1 2 T A C C A T
6 1 1 1 1 2 T T C C A T
```

b.map:

```
1 rs571227  0 738547
0 rs2827188 0 5597094
X rs2835744 0 9424115
```

c) Use command "coPLINK --tped2ped a --out b --nor --phenotype 2 --rnd-sex" to re-generate PED files with main filename b.

b.ped:

```
1 1 1 1 2 2 d D d D D d D D
2 1 1 1 1 2 d D D d D d D D
1 2 1 1 1 2 D D d d D D D D
1 2 2 2 2 2 d d D D d d D N
5 1 1 1 1 2 D d D D d D D D
6 1 1 1 1 2 D D D D d D N D
```

b.map:

```
1 rs571227  0 738547
0 rs2827188 0 5597094
X rs2835744 0 9424115
Y rs181146  0 1387981
```

d) Remove a.tsnp, and type command "coPLINK --tped2ped a --out b" to re-generate PED files with main filename b.

b.ped:

```
1 1 1 1 1 1 A T G C T A C C
2 1 1 1 2 1 A T C G T A C C
1 2 1 1 1 1 T T G G T T C C
1 2 2 2 2 2 A A C C A A C N
```

```
5 1 1 1 1 2 T A C C A T C C
6 1 1 1 1 2 T T C C A T N C
```

b.map:

```
1 EGAV1 0 738547
0 EGAV2 0 5597094
X EGAV3 0 9424115
Y EGAV4 0 1387981
```

44 --tped2usr-tped

This parameter is used to convert a group of user-defined TPED (see --ped2tped) files to TPED format.

Like --tped2bed, the parameter does not care about the first three columns in a .tnsp file too. And, it will check whether there is a .tsnp file in the path of source files. If it is, it gets RS#s from the file, otherwise takes them from the .tped file.

The indicators supported by the parameter include (See previous specifications to find the functions of the top 3 indicators):

- --pre
- --hwe
- --nor -1
  - Tell the program not to count the numbers of major and minor alleles thus to reduce run-time.
  - Used only when the source is a normalized data such as a TPED data converted from a normalized PED data.
- --phenotype
- --out
  - Specify the output main filename of user-defined TPED format.

Example 44: Suppose we have a file-set of TPED format are the same as example 42.

a) Use command "coPLINK --tped2usr-tped a --out b" to generate a group of user-defined TPED files with main filename b.

b.tped:

```
1 rs571227  0 738547  1 1 0  2 1  0
0 rs2827188 0 5597094 1 1 2  0 0  0
X rs2835744 0 9424115 1 1 0  2 1  1
Y rs181146  0 1387981 0 0 0 -1 0 -1
```

b.tfam:

```
1 1 1 1 1 1
2 1 1 1 2 1
1 2 1 1 1 1
1 2 2 2 2 2
5 1 1 1 1 2
6 1 1 1 1 2
```

b) Use command "coPLINK --tped2usr-tped a --out b --pre 0.2" to re-generate user-defined TPED files with main filename b.

b.tped:

```
1 rs571227  0 738547  1 1 0 2 1 0
0 rs2827188 0 5597094 1 1 2 0 0 0
X rs2835744 0 9424115 1 1 0 2 1 1
```

b.tfam:

```
1 1 1 1 1 1
2 1 1 1 2 1
1 2 1 1 1 1
1 2 2 2 2 2
5 1 1 1 1 2
6 1 1 1 1 2
```

c) Use command "coPLINK --tped2usr-tped a --out b --phenotype 2" to re-generate TPED files with main filename b while setting the phenotypes to "2".

b.tped:

```
1 rs571227  0 738547  1 1 0  2 1  0
0 rs2827188 0 5597094 1 1 2  0 0  0
X rs2835744 0 9424115 1 1 0  2 1  1
Y rs181146  0 1387981 0 0 0 -1 0 -1
```

b.tfam:

```
1 1 1 1 1 2
2 1 1 1 2 2
1 2 1 1 1 2
1 2 2 2 2 2
5 1 1 1 1 2
6 1 1 1 1 2
```

d) Remove a.tsnp, and type command "coPLINK --tped2usr-tped a --out b" to re-generate TPED files with main filename b.

b.tped:

```
1 EGAV1 0 738547   1 1 0  2 1  0
0 EGAV2 0 5597094  1 1 2  0 0  0
X EGAV3 0 9424115  1 1 0  2 1  1
Y EGAV4 0 1387981  0 0 0 -1 0 -1
```

b.tfam:

```
1 1 1 1 1 1
2 1 1 1 2 1
1 2 1 1 1 1
1 2 2 2 2 2
5 1 1 1 1 2
6 1 1 1 1 2
```

45 --transpose

This parameter is used to transpose a plain-text file of any format. The result will reserve only one space or TAB between two strings regardless of the number of the source, and will not reserve the null rows at the end of the source.

The indicators supported by the parameter include:

- --delim
  - Specify the delimiter of the source. Default is space.
  - Escape character "\t" represents a TAB.
  - If the source mixes spaces and TABs as delimiters, the delimiter in the result file is only the specified space or TAB.
- --mode
  - Specify the running mode of the program. 0 - auto (default); 1 - memory first.
  - The parameter supports two running mode, speed first and memory first. The speed first mode has faster speed but more run-time memory (3~12 times of the size of the source relating to the numbers of columns and rows), and the other has slower speed but less run-time memory (about 1 times of the size of the source). Auto mode can detect the free memory to determine which mode will be used.
- --out
  - Specify the output filename.

Example 45:

a) Suppose we have a file a.tped as listed below. Use command "coPLINK --transpose a.tped --out b.tped" to generate a transposed file.

a.tped:

```
1 EGAV1 0 738547   A T A T T T A A T A T T
0 EGAV2 0 5597094  G C C G G G C C C C C C
X EGAV3 0 9424115  T A T A T T A A A T A T
Y EGAV4 0 1387981  C C C C C C C N C C N C
```

b.tped:

```
1 0 X Y
EGAV1 EGAV2 EGAV3 EGAV4
0 0 0 0
738547 5597094 9424115 1387981
A G T C
T C A C
A C T C
T G A C
T G T C
T G T C
A C A C
A C A N
T C A C
A C T C
T C A N
T C T C
```

b) Re-run the command again by --mode 1.

```
1 0 X Y
EGAV1 EGAV2 EGAV3 EGAV4
0 0 0 0
738547 5597094 9424115 1387981
A G T C
T C A C
A C T C
T G A C
T G T C
T G T C
A C A C
A C A N
```

```
T C A C
A C T C
T C A N
T C T C
```

c) Suppose another file a.txt as listed below needs be transposed to b.txt. Type command "coPLINK --transpose a.txt --out b.txt" to reach the target.

a.txt:

```
In linear algebra,
the transpose of a matrix is an operator which flips a matrix over its diagonal,
that is it switches the row and column indices of the matrix by producing another matrix denoted as A'.
```

b.txt:

```
In the that
linear transpose is
algebra, of it
 a switches
 matrix the
 is row
 an and
 operator column
 which indices
 flips of
 a the
 matrix matrix
 over by
 its producing
 diagonal, another
  matrix
  denoted
  as
  A'.
```

From b.txt, we can learn that there are some spaces at the beginning of the rows starting from row 4, which is caused by the one by one increase in the length of the row in the source.

d) A comma delimited file a.csv listed in Excel as shown below. Command "coPLINK --transpose a.csv --out b.csv --delim ," transposes a.csv into b.csv.

a.csv:

0	SNP4	0	0
0	SNP3	0	0
0	SNP2	0	1
0	SNP1	0	0

b.csv:

0	0	0	0
SNP4	SNP3	SNP2	SNP1
0	0	0	0
0	0	1	0

History

- V1: May, 2012. Implement the conversion of BEAM, BOOST, MDR and ME formats to PLINK PED/BED and inverse conversion, and the conversion between CSV and SPACE each other, as well as PED splicing, comparison, deleting and inserting functions.
- V1.1: July, 2012. Fix bugs, optimize algorithms and add/adjust some indcators.
- V2: April, 2013. Add Linkage, GS-Linkage, LogicReg, PrettyBase, SVMSNPs and GEO conversions. The parameters '--ped2other' and '--other2ped' were added.
- V2.1~2.4: May~June, 2013. Fix bugs, optimize algorithms and add/adjust some indcators.
- V3: October, 2013. Add TPED and Usr-TPED conversions, as well as the parameters '--transpose' and '--test-allele'.
- October, 2013 ~ December, 2019: Fix bugs, optimize algorithms and add/adjust some indcators.
- V4: Feburary, 2020. Add automation, reorgnize codes, improve the prompts of information and errors, and write the manual.
