# Supplementary material for: coPLINK: A complementary tool to PLINK
Source: PLoS One. 2020 Sep 18;15(9):e0239144. doi: 10.1371/journal.pone.0239144 (PMC7500662; doi:10.1371/journal.pone.0239144)
Supplement: S2 File — (DOCX) [file pone.0239144.s002.docx]

**Supplementary 2**

# Details of functional test

## Test cases of functional test

The steps of functional test by using spliced CAD data as the input are as follows:

1. Use the parameter "--ped2bed" with indicator "--nor 1" to generate a normalized PED file while converting the PED format file to a BED format. Its purpose is to ensure that SNPs with the same number of major and minor alleles in BED and PED files have the same composition of major and minor alleles.
2. Convert the normalized PED file into two files of BOOST format, one is not swapped, the other is swapped.
3. Use the tested function to convert the normalized PED file.
4. If there is no loss of information (such as the family ID in the PED) or alleles swapping during the conversion, compare the result with the PED or BED file. Otherwise, convert the result to BOOST further, and then compared respectively the new result with the two BOOST files produced in step (1). As long as one of the comparisons succeeds, the function is considered correct. In all conversions, add indicator "--nor -1" when a parameter supports it, to ensure that SNPs with the same number of major and minor alleles will not swap the major and minor alleles.

The test cases and results of the functional test with real data as input are shown in Table S1.

**Table S1** Test cases and results of functional test

| **Test case** | **Tested function** | **Passed** |
| --- | --- | --- |
| --ped2beam 🡪 --beam2ped 🡪--ped2boost | --ped2beam, --beam2ped | Yes |
| --ped2beam 🡪--beam2boost | --ped2beam, --beam2boost | Yes |
| --bed2beam 🡪--beam2ped 🡪--ped2boost | --bed2beam, --beam2ped | Yes |
| --bed2boost 🡪--boost2ped 🡪--ped2boost | --bed2boost, --boost2ped | Yes |
| --bed2me 🡪--me2boost | --bed2me, --me2boost | Yes |
| --bed2tped 🡪--tped2ped 🡪--ped2boost | --bed2tped, --tped2ped | Yes |
| --bed2boost 🡪--boost2bed 🡪--bed2boost | --bed2boost, --boost2bed | Yes |
| --space2csv 🡪--csv2space^(a)^ | --space2csv, --csv2space | Yes |
| --ped2geo 🡪--geo2ped 🡪--ped2boost | --ped2geo, --geo2ped | Yes |
| --ped2gs-linkage 🡪--gs-linkage2ped 🡪--ped2boost | --ped2gs-linkage, --gs-linkage2ped | Yes |
| --ped2linkage 🡪--linkage2boost | --ped2linkage, --linkage2boost | Yes |
| --ped2linkage 🡪--linkage2ped 🡪--ped2boost^(b)^ | --linkage2ped | Yes |
| --ped2logicreg 🡪--logicreg2ped 🡪--ped2boost | --ped2logicreg, --logicreg2ped | Yes |
| --ped2mdr 🡪--mdr2boost | --ped2mdr, --mdr2boost | Yes |
| --ped2mdr 🡪--mdr2ped 🡪--ped2boost | --mdr2ped | Yes |
| --ped2me 🡪--me2ped 🡪--ped2boost | --ped2me, --me2ped | Yes |
| --ped2bed 🡪--bed2boost | --ped2bed | Yes |
| --ped2other 🡪--other2ped ^(c)^ | --ped2other, -- other2ped | Yes |
| --ped2svmsnp 🡪--svmsnp2ped 🡪--ped2ped --nor 🡪--ped2boost | --ped2svmsnp, --svmsnp2ped | Yes |
| --ped2tped 🡪--tped2ped 🡪 --ped2boost | --ped2tped, --tped2ped | Yes |
| --tped2bed 🡪--bed2boost | --tped2bed | Yes |
| --ped2tped 🡪--tped2usr-tped 🡪--other2ped^(d)^ 🡪--ped2boost | --tped2usr-tped | Yes |
| --transpose 🡪--transpose^(e)^ | --transpose | Yes |

^(a)^Use --space2csv to convert the space delimited .ped file into comma delimited, and then use --csv2space to restore to space delimited. ^(b)^Don't append indicator '--nor -1', because '--linkage2ped' may swap the alleles. ^(c)^Use --ped2other to convert .ped files into .tped files, and then use --other2ped to restore to .ped files. ^(d)^Use --other2ped to convert the files of usr-tped format into ped format. ^(e)^Use the parameter twice to restore the .ped file to the original file.

## Script in Windows OS for functional test

The script shown in List S1 can test all converting functions of coPLINK and produce a test results named as coPLINK-Test-Res.csv.

**List S1** Script of functional test

| for /l %%i in (1,1,9) do (  del $*.*  coPLINK --ped2bed With_NBS_Ctrls_Affx_gt_CAD_Chiamo_0%%i --out $bed --file2 $nor --nor 1  coPLINK --ped2boost $nor --out $cmp --nor -1  coPLINK --ped2boost $nor --out $cmp-swap --swap --nor -1  coPLINK --ped2beam $nor --out $1 --nor -1  coPLINK --beam2boost $1 --out $2 --nor -1  coPLINK --compare $2.txt --file2 $cmp.txt --out coPLINK-Test-Res.csv --memo ped2beam_0%%i  coPLINK --compare $2.txt --file2 $cmp-swap.txt --out coPLINK-Test-Res.csv --memo ped2beam_0%%i_Swap  coPLINK --bed2beam $bed --out $1  coPLINK --beam2ped $1 --out $2  coPLINK --ped2boost $2 --out $1 --nor -1  coPLINK --compare $1.txt --file2 $cmp.txt --out coPLINK-Test-Res.csv --memo bed2beam_0%%i  coPLINK --compare $1.txt --file2 $cmp-swap.txt --out coPLINK-Test-Res.csv --memo bed2beam_0%%i_Swap  coPLINK --bed2boost $bed --out $1  coPLINK --boost2ped $1 --out $2  coPLINK --ped2boost $2 --out $1 --nor -1  coPLINK --compare $1.txt --file2 $cmp.txt --out coPLINK-Test-Res.csv --memo bed2boost_0%%i  coPLINK --compare $1.txt --file2 $cmp-swap.txt --out coPLINK-Test-Res.csv --memo bed2boost_0%%i_Swap  coPLINK --bed2me $bed --out $1  coPLINK --me2boost $1.me --out $2  coPLINK --compare $2.txt --file2 $cmp.txt --out coPLINK-Test-Res.csv --memo bed2me_0%%i  coPLINK --compare $2.txt --file2 $cmp-swap.txt --out coPLINK-Test-Res.csv --memo bed2me_0%%i_Swap  coPLINK --bed2tped $bed --out $1  coPLINK --tped2ped $1 --out $2  coPLINK --ped2boost $2 --out $1 --nor -1  coPLINK --compare $1.txt --file2 $cmp.txt --out coPLINK-Test-Res.csv --memo bed2tped_0%%i  coPLINK --compare $1.txt --file2 $cmp-swap.txt --out coPLINK-Test-Res.csv --memo bed2tped_0%%i_Swap  coPLINK --bed2boost $bed --out $1  coPLINK --boost2bed $1 --out $2  coPLINK --bed2boost $2 --out $1  coPLINK --compare $1.txt --file2 $cmp.txt --out coPLINK-Test-Res.csv --memo bed2boost_0%%i  coPLINK --compare $1.txt --file2 $cmp-swap.txt --out coPLINK-Test-Res.csv --memo bed2boost_0%%i_Swap  coPLINK --space2csv $nor.ped --out $1.csv  coPLINK --csv2space $1.csv --out $2.txt  coPLINK --compare $2.txt --file2 $nor.ped --out coPLINK-Test-Res.csv --memo space2csv_0%%i  coPLINK --ped2geo $nor --out $1 --nor -1  coPLINK --geo2ped $1 --out $2 --nor -1  coPLINK --ped2boost $2 --out $1 --nor -1  coPLINK --compare $1.txt --file2 $cmp.txt --out coPLINK-Test-Res.csv --memo ped2geo_0%%i  coPLINK --compare $1.txt --file2 $cmp-swap.txt --out coPLINK-Test-Res.csv --memo ped2geo_0%%i_Swap  coPLINK --ped2gs-linkage $nor --out $1 --nor -1  coPLINK --gs-linkage2ped $1 --out $2  coPLINK --ped2boost $2 --out $1 --nor -1  coPLINK --compare $1.txt --file2 $cmp.txt --out coPLINK-Test-Res.csv --memo ped2gs-linkage_0%%i  coPLINK --compare $1.txt --file2 $cmp-swap.txt --out coPLINK-Test-Res.csv --memo ped2gs-linkage_0%%i_Swap  coPLINK --ped2linkage $nor --out $1  coPLINK --linkage2boost $1 --out $2  coPLINK --compare $2.txt --file2 $cmp.txt --out coPLINK-Test-Res.csv --memo ped2linkage_0%%i  coPLINK --compare $2.txt --file2 $cmp-swap.txt --out coPLINK-Test-Res.csv --memo ped2linkage_0%%i_Swap  coPLINK --ped2linkage $nor --out $1  coPLINK --linkage2ped $1 --out $2  coPLINK --ped2boost $2 --out $1  coPLINK --compare $1.txt --file2 $cmp.txt --out coPLINK-Test-Res.csv --memo linkage2ped_0%%i  coPLINK --compare $1.txt --file2 $cmp-swap.txt --out coPLINK-Test-Res.csv --memo linkage2ped_0%%i_Swap  coPLINK --ped2logicreg $nor --out $1 --nor -1  coPLINK --logicreg2ped $1 --out $2 --nor -1  coPLINK --ped2boost $2 --out $1 --nor -1  coPLINK --compare $1.txt --file2 $cmp.txt --out coPLINK-Test-Res.csv --memo ped2logicreg_0%%i  coPLINK --compare $1.txt --file2 $cmp-swap.txt --out coPLINK-Test-Res.csv --memo ped2logicreg_0%%i_Swap  coPLINK --ped2mdr $nor --out $1 --nor -1  coPLINK --mdr2boost $1 --out $2 --nor -1  coPLINK --compare $2.txt --file2 $cmp.txt --out coPLINK-Test-Res.csv --memo ped2mdr_0%%i  coPLINK --compare $2.txt --file2 $cmp-swap.txt --out coPLINK-Test-Res.csv --memo ped2mdr_0%%i_Swap  coPLINK --ped2mdr $nor --out $1 --nor -1  coPLINK --mdr2ped $1 --out $2 --nor -1  coPLINK --ped2boost $2 --out $1 --nor -1  coPLINK --compare $1.txt --file2 $cmp.txt --out coPLINK-Test-Res.csv --memo mdr2ped_0%%i  coPLINK --compare $1.txt --file2 $cmp-swap.txt --out coPLINK-Test-Res.csv --memo mdr2ped_0%%i_Swap  coPLINK --ped2me $nor --out $1 --nor -1  coPLINK --me2ped $1.me --out $2 --nor -1  coPLINK --ped2boost $2 --out $1 --nor -1  coPLINK --compare $1.txt --file2 $cmp.txt --out coPLINK-Test-Res.csv --memo ped2me_0%%i  coPLINK --compare $1.txt --file2 $cmp-swap.txt --out coPLINK-Test-Res.csv --memo ped2me_0%%i_Swap  coPLINK --ped2bed $nor --out $1 --nor -1  coPLINK --bed2boost $1 --out $2 --nor -1  coPLINK --compare $2.txt --file2 $cmp.txt --out coPLINK-Test-Res.csv --memo ped2bed_0%%i  coPLINK --compare $2.txt --file2 $cmp-swap.txt --out coPLINK-Test-Res.csv --memo ped2bed_0%%i_Swap  coPLINK --ped2other p2tped.txt --file2 $nor --out $1  coPLINK --other2ped tped2p.txt --out $2  coPLINK --compare $2.ped --file2 $nor.ped --out coPLINK-Test-Res.csv --memo ped2other_0%%i  rem --pretty2ped set the phenotypes to default 1, since Prettybase doesn't include phenotypes.  coPLINK --ped2boost $nor --out $cmp1 --nor -1 --phenotype 1  coPLINK --ped2boost $nor --out $cmp1-swap --swap --nor -1 --phenotype 1  coPLINK --ped2pretty $nor --out $1  coPLINK --pretty2ped $1.pretty --out $2  coPLINK --ped2boost $2 --out $1 --nor -1  coPLINK --compare $1.txt --file2 $cmp1.txt --out coPLINK-Test-Res.csv --memo ped2pretty_0%%i  coPLINK --compare $1.txt --file2 $cmp1-swap.txt --out coPLINK-Test-Res.csv --memo ped2pretty_0%%i_Swap  coPLINK --ped2svmsnp $nor --out $1 --mode 1 --nor -1  coPLINK --svmsnp2ped $1 --out $2 --mode 1 --nor -1  coPLINK --ped2ped $2 --out $1 --nor  coPLINK --ped2boost $1 --out $2 --nor -1  coPLINK --compare $2.txt --file2 $cmp.txt --out coPLINK-Test-Res.csv --memo ped2svmsnp_0%%i  coPLINK --compare $2.txt --file2 $cmp-swap.txt --out coPLINK-Test-Res.csv --memo ped2svmsnp_0%%i_Swap  coPLINK --ped2tped $nor --out $1 --nor -1  coPLINK --tped2ped $1 --out $2 --nor -1  coPLINK --ped2boost $2 --out $1 --nor -1  coPLINK --compare $1.txt --file2 $cmp.txt --out coPLINK-Test-Res.csv --memo ped2tped_0%%i  coPLINK --compare $1.txt --file2 $cmp-swap.txt --out coPLINK-Test-Res.csv --memo ped2tped_0%%i_Swap  coPLINK --ped2tped $nor --out $1 --nor -1  coPLINK --tped2bed $1 --out $2 --nor -1  coPLINK --bed2boost $2 --out $1  coPLINK --compare $1.txt --file2 $cmp.txt --out coPLINK-Test-Res.csv --memo tped2bed_0%%i  coPLINK --compare $1.txt --file2 $cmp-swap.txt --out coPLINK-Test-Res.csv --memo tped2bed_0%%i_Swap  coPLINK --ped2tped $nor --out $1 --nor -1  coPLINK --tped2usr-tped $1 --out $2 --nor -1  coPLINK --other2ped usrt2p.txt --out $1  coPLINK --ped2boost $1 --out $2 --nor -1  coPLINK --compare $2.txt --file2 $cmp.txt --out coPLINK-Test-Res.csv --memo tped2usr-tped_0%%i  coPLINK --compare $2.txt --file2 $cmp-swap.txt --out coPLINK-Test-Res.csv --memo tped2usr-tped_0%%i_Swap  coPLINK --transpose $nor.ped --out $1.txt  coPLINK --transpose $1.txt --out $2.txt  coPLINK --compare $2.txt --file2 $nor.ped --out coPLINK-Test-Res.csv --memo transpose_0%%i  )  for /l %%i in (10,1,22) do (  del $*.*  coPLINK --ped2bed With_NBS_Ctrls_Affx_gt_CAD_Chiamo_%%i --out $bed --file2 $nor --nor 1  coPLINK --ped2boost $nor --out $cmp  coPLINK --ped2boost $nor --out $cmp-swap --swap  coPLINK --ped2beam $nor --out $1 --nor -1  coPLINK --beam2boost $1 --out $2 --nor -1  coPLINK --compare $2.txt --file2 $cmp.txt --out coPLINK-Test-Res.csv --memo ped2beam_%%i  coPLINK --compare $2.txt --file2 $cmp-swap.txt --out coPLINK-Test-Res.csv --memo ped2beam_%%i_Swap  coPLINK --bed2beam $bed --out $1  coPLINK --beam2ped $1 --out $2  coPLINK --ped2boost $2 --out $1 --nor -1  coPLINK --compare $1.txt --file2 $cmp.txt --out coPLINK-Test-Res.csv --memo bed2beam_%%i  coPLINK --compare $1.txt --file2 $cmp-swap.txt --out coPLINK-Test-Res.csv --memo bed2beam_%%i_Swap  coPLINK --bed2boost $bed --out $1  coPLINK --boost2ped $1 --out $2  coPLINK --ped2boost $2 --out $1 --nor -1  coPLINK --compare $1.txt --file2 $cmp.txt --out coPLINK-Test-Res.csv --memo bed2boost_%%i  coPLINK --compare $1.txt --file2 $cmp-swap.txt --out coPLINK-Test-Res.csv --memo bed2boost_%%i_Swap  coPLINK --bed2me $bed --out $1  coPLINK --me2boost $1.me --out $2  coPLINK --compare $2.txt --file2 $cmp.txt --out coPLINK-Test-Res.csv --memo bed2me_%%i  coPLINK --compare $2.txt --file2 $cmp-swap.txt --out coPLINK-Test-Res.csv --memo bed2me_%%i_Swap  coPLINK --bed2tped $bed --out $1  coPLINK --tped2ped $1 --out $2  coPLINK --ped2boost $2 --out $1 --nor -1  coPLINK --compare $1.txt --file2 $cmp.txt --out coPLINK-Test-Res.csv --memo bed2tped_%%i  coPLINK --compare $1.txt --file2 $cmp-swap.txt --out coPLINK-Test-Res.csv --memo bed2tped_%%i_Swap  coPLINK --bed2boost $bed --out $1  coPLINK --boost2bed $1 --out $2  coPLINK --bed2boost $2 --out $1  coPLINK --compare $1.txt --file2 $cmp.txt --out coPLINK-Test-Res.csv --memo bed2boost_%%i  coPLINK --compare $1.txt --file2 $cmp-swap.txt --out coPLINK-Test-Res.csv --memo bed2boost_%%i_Swap  coPLINK --space2csv $nor.ped --out $1.csv  coPLINK --csv2space $1.csv --out $2.txt  coPLINK --compare $2.txt --file2 $nor.ped --out coPLINK-Test-Res.csv --memo space2csv_%%i  coPLINK --ped2geo $nor --out $1 --nor -1  coPLINK --geo2ped $1 --out $2 --nor -1  coPLINK --ped2boost $2 --out $1 --nor -1  coPLINK --compare $1.txt --file2 $cmp.txt --out coPLINK-Test-Res.csv --memo ped2geo_%%i  coPLINK --compare $1.txt --file2 $cmp-swap.txt --out coPLINK-Test-Res.csv --memo ped2geo_%%i_Swap  coPLINK --ped2gs-linkage $nor --out $1 --nor -1  coPLINK --gs-linkage2ped $1 --out $2  coPLINK --ped2boost $2 --out $1 --nor -1  coPLINK --compare $1.txt --file2 $cmp.txt --out coPLINK-Test-Res.csv --memo ped2gs-linkage_%%i  coPLINK --compare $1.txt --file2 $cmp-swap.txt --out coPLINK-Test-Res.csv --memo ped2gs-linkage_%%i_Swap  coPLINK --ped2linkage $nor --out $1  coPLINK --linkage2boost $1 --out $2  coPLINK --compare $2.txt --file2 $cmp.txt --out coPLINK-Test-Res.csv --memo ped2linkage_%%i  coPLINK --compare $2.txt --file2 $cmp-swap.txt --out coPLINK-Test-Res.csv --memo ped2linkage_%%i_Swap  coPLINK --ped2linkage $nor --out $1  coPLINK --linkage2ped $1 --out $2  coPLINK --ped2boost $2 --out $1  coPLINK --compare $1.txt --file2 $cmp.txt --out coPLINK-Test-Res.csv --memo linkage2ped_%%i  coPLINK --compare $1.txt --file2 $cmp-swap.txt --out coPLINK-Test-Res.csv --memo linkage2ped_%%i_Swap  coPLINK --ped2logicreg $nor --out $1 --nor -1  coPLINK --logicreg2ped $1 --out $2 --nor -1  coPLINK --ped2boost $2 --out $1 --nor -1  coPLINK --compare $1.txt --file2 $cmp.txt --out coPLINK-Test-Res.csv --memo ped2logicreg_%%i  coPLINK --compare $1.txt --file2 $cmp-swap.txt --out coPLINK-Test-Res.csv --memo ped2logicreg_%%i_Swap  coPLINK --ped2mdr $nor --out $1 --nor -1  coPLINK --mdr2boost $1 --out $2 --nor -1  coPLINK --compare $2.txt --file2 $cmp.txt --out coPLINK-Test-Res.csv --memo ped2mdr_%%i  coPLINK --compare $2.txt --file2 $cmp-swap.txt --out coPLINK-Test-Res.csv --memo ped2mdr_%%i_Swap  coPLINK --ped2mdr $nor --out $1 --nor -1  coPLINK --mdr2ped $1 --out $2 --nor -1  coPLINK --ped2boost $2 --out $1 --nor -1  coPLINK --compare $1.txt --file2 $cmp.txt --out coPLINK-Test-Res.csv --memo mdr2ped_%%i  coPLINK --compare $1.txt --file2 $cmp-swap.txt --out coPLINK-Test-Res.csv --memo mdr2ped_%%i_Swap  coPLINK --ped2me $nor --out $1 --nor -1  coPLINK --me2ped $1 --out $2 --nor -1  coPLINK --ped2boost $2 --out $1 --nor -1  coPLINK --compare $1.txt --file2 $cmp.txt --out coPLINK-Test-Res.csv --memo ped2me_%%i  coPLINK --compare $1.txt --file2 $cmp-swap.txt --out coPLINK-Test-Res.csv --memo ped2me_%%i_Swap  coPLINK --ped2bed $nor --out $1 --nor -1  coPLINK --bed2boost $1 --out $2 --nor -1  coPLINK --compare $2.txt --file2 $cmp.txt --out coPLINK-Test-Res.csv --memo ped2bed_%%i  coPLINK --compare $2.txt --file2 $cmp-swap.txt --out coPLINK-Test-Res.csv --memo ped2bed_%%i_Swap  coPLINK --ped2other p2tped.txt --file2 $nor --out $1  coPLINK --other2ped tped2p.txt --out $2  coPLINK --compare $2.ped --file2 $nor.ped --out coPLINK-Test-Res.csv --memo ped2other_%%i  rem --pretty2ped set the phenotypes to default 1, since Prettybase doesn't include phenotypes.  coPLINK --ped2boost $nor --out $cmp1 --nor -1 --phenotype 1  coPLINK --ped2boost $nor --out $cmp1-swap --swap --nor -1 --phenotype 1  coPLINK --ped2pretty $nor --out $1  coPLINK --pretty2ped $1.pretty --out $2  coPLINK --ped2boost $2 --out $1 --nor -1  coPLINK --compare $1.txt --file2 $cmp1.txt --out coPLINK-Test-Res.csv --memo ped2pretty_%%i  coPLINK --compare $1.txt --file2 $cmp1-swap.txt --out coPLINK-Test-Res.csv --memo ped2pretty_%%i_Swap  coPLINK --ped2svmsnp $nor --out $1 --mode 1 --nor -1  coPLINK --svmsnp2ped $1 --out $2 --mode 1 --nor -1  coPLINK --ped2ped $2 --out $1 --nor  coPLINK --ped2boost $1 --out $2 --nor -1  coPLINK --compare $2.txt --file2 $cmp.txt --out coPLINK-Test-Res.csv --memo ped2svmsnp_%%i  coPLINK --compare $2.txt --file2 $cmp-swap.txt --out coPLINK-Test-Res.csv --memo ped2svmsnp_%%i_Swap  coPLINK --ped2tped $nor --out $1 --nor -1  coPLINK --tped2ped $1 --out $2 --nor -1  coPLINK --ped2boost $2 --out $1 --nor -1  coPLINK --compare $1.txt --file2 $cmp.txt --out coPLINK-Test-Res.csv --memo ped2tped_%%i  coPLINK --compare $1.txt --file2 $cmp-swap.txt --out coPLINK-Test-Res.csv --memo ped2tped_%%i_Swap  coPLINK --ped2tped $nor --out $1 --nor -1  coPLINK --tped2bed $1 --out $2 --nor -1  coPLINK --bed2boost $2 --out $1  coPLINK --compare $1.txt --file2 $cmp.txt --out coPLINK-Test-Res.csv --memo tped2bed_%%i  coPLINK --compare $1.txt --file2 $cmp-swap.txt --out coPLINK-Test-Res.csv --memo tped2bed_%%i_Swap  coPLINK --ped2tped $nor --out $1 --nor -1  coPLINK --tped2usr-tped $1 --out $2 --nor -1  coPLINK --other2ped usrt2p.txt --out $1  coPLINK --ped2boost $1 --out $2 --nor -1  coPLINK --compare $2.txt --file2 $cmp.txt --out coPLINK-Test-Res.csv --memo tped2usr-tped_%%i  coPLINK --compare $2.txt --file2 $cmp-swap.txt --out coPLINK-Test-Res.csv --memo tped2usr-tped_%%i_Swap  coPLINK --transpose $nor.ped --out $1.txt  coPLINK --transpose $1.txt --out $2.txt  coPLINK --compare $2.txt --file2 $nor.ped --out coPLINK-Test-Res.csv --memo transpose_%%i  )  del $*.*  coPLINK --ped2bed With_NBS_Ctrls_Affx_gt_CAD_Chiamo_X --out $bed --file2 $nor --nor 1  coPLINK --ped2boost $nor --out $cmp  coPLINK --ped2boost $nor --out $cmp-swap --swap  coPLINK --ped2beam $nor --out $1 --nor -1  coPLINK --beam2boost $1 --out $2 --nor -1  coPLINK --compare $2.txt --file2 $cmp.txt --out coPLINK-Test-Res.csv --memo ped2beam_X  coPLINK --compare $2.txt --file2 $cmp-swap.txt --out coPLINK-Test-Res.csv --memo ped2beam_X_Swap  coPLINK --bed2beam $bed --out $1  coPLINK --beam2ped $1 --out $2  coPLINK --ped2boost $2 --out $1 --nor -1  coPLINK --compare $1.txt --file2 $cmp.txt --out coPLINK-Test-Res.csv --memo bed2beam_X  coPLINK --compare $1.txt --file2 $cmp-swap.txt --out coPLINK-Test-Res.csv --memo bed2beam_X_Swap  coPLINK --bed2boost $bed --out $1  coPLINK --boost2ped $1 --out $2  coPLINK --ped2boost $2 --out $1 --nor -1  coPLINK --compare $1.txt --file2 $cmp.txt --out coPLINK-Test-Res.csv --memo bed2boost_X  coPLINK --compare $1.txt --file2 $cmp-swap.txt --out coPLINK-Test-Res.csv --memo bed2boost_X_Swap  coPLINK --bed2me $bed --out $1  coPLINK --me2boost $1.me --out $2  coPLINK --compare $2.txt --file2 $cmp.txt --out coPLINK-Test-Res.csv --memo bed2me_X  coPLINK --compare $2.txt --file2 $cmp-swap.txt --out coPLINK-Test-Res.csv --memo bed2me_X_Swap  coPLINK --bed2tped $bed --out $1  coPLINK --tped2ped $1 --out $2  coPLINK --ped2boost $2 --out $1 --nor -1  coPLINK --compare $1.txt --file2 $cmp.txt --out coPLINK-Test-Res.csv --memo bed2tped_X  coPLINK --compare $1.txt --file2 $cmp-swap.txt --out coPLINK-Test-Res.csv --memo bed2tped_X_Swap  coPLINK --bed2boost $bed --out $1  coPLINK --boost2bed $1 --out $2  coPLINK --bed2boost $2 --out $1  coPLINK --compare $1.txt --file2 $cmp.txt --out coPLINK-Test-Res.csv --memo bed2boost_X  coPLINK --compare $1.txt --file2 $cmp-swap.txt --out coPLINK-Test-Res.csv --memo bed2boost_X_Swap  coPLINK --space2csv $nor.ped --out $1.csv  coPLINK --csv2space $1.csv --out $2.txt  coPLINK --compare $2.txt --file2 $nor.ped --out coPLINK-Test-Res.csv --memo space2csv_X  coPLINK --ped2geo $nor --out $1 --nor -1  coPLINK --geo2ped $1 --out $2 --nor -1  coPLINK --ped2boost $2 --out $1 --nor -1  coPLINK --compare $1.txt --file2 $cmp.txt --out coPLINK-Test-Res.csv --memo ped2geo_X  coPLINK --compare $1.txt --file2 $cmp-swap.txt --out coPLINK-Test-Res.csv --memo ped2geo_X_Swap  coPLINK --ped2gs-linkage $nor --out $1 --nor -1  coPLINK --gs-linkage2ped $1 --out $2  coPLINK --ped2boost $2 --out $1 --nor -1  coPLINK --compare $1.txt --file2 $cmp.txt --out coPLINK-Test-Res.csv --memo ped2gs-linkage_X  coPLINK --compare $1.txt --file2 $cmp-swap.txt --out coPLINK-Test-Res.csv --memo ped2gs-linkage_X_Swap  coPLINK --ped2linkage $nor --out $1  coPLINK --linkage2boost $1 --out $2  coPLINK --compare $2.txt --file2 $cmp.txt --out coPLINK-Test-Res.csv --memo ped2linkage_X  coPLINK --compare $2.txt --file2 $cmp-swap.txt --out coPLINK-Test-Res.csv --memo ped2linkage_X_Swap  coPLINK --ped2linkage $nor --out $1  coPLINK --linkage2ped $1 --out $2  coPLINK --ped2boost $2 --out $1  coPLINK --compare $1.txt --file2 $cmp.txt --out coPLINK-Test-Res.csv --memo linkage2ped_X  coPLINK --compare $1.txt --file2 $cmp-swap.txt --out coPLINK-Test-Res.csv --memo linkage2ped_X_Swap  coPLINK --ped2logicreg $nor --out $1 --nor -1  coPLINK --logicreg2ped $1 --out $2 --nor -1  coPLINK --ped2boost $2 --out $1 --nor -1  coPLINK --compare $1.txt --file2 $cmp.txt --out coPLINK-Test-Res.csv --memo ped2logicreg_X  coPLINK --compare $1.txt --file2 $cmp-swap.txt --out coPLINK-Test-Res.csv --memo ped2logicreg_X_Swap  coPLINK --ped2mdr $nor --out $1 --nor -1  coPLINK --mdr2boost $1 --out $2 --nor -1  coPLINK --compare $2.txt --file2 $cmp.txt --out coPLINK-Test-Res.csv --memo ped2mdr_X  coPLINK --compare $2.txt --file2 $cmp-swap.txt --out coPLINK-Test-Res.csv --memo ped2mdr_X_Swap  coPLINK --ped2mdr $nor --out $1 --nor -1  coPLINK --mdr2ped $1 --out $2 --nor -1  coPLINK --ped2boost $2 --out $1 --nor -1  coPLINK --compare $1.txt --file2 $cmp.txt --out coPLINK-Test-Res.csv --memo mdr2ped_X  coPLINK --compare $1.txt --file2 $cmp-swap.txt --out coPLINK-Test-Res.csv --memo mdr2ped_X_Swap  coPLINK --ped2me $nor --out $1 --nor -1  coPLINK --me2ped $1 --out $2 --nor -1  coPLINK --ped2boost $2 --out $1 --nor -1  coPLINK --compare $1.txt --file2 $cmp.txt --out coPLINK-Test-Res.csv --memo ped2me_X  coPLINK --compare $1.txt --file2 $cmp-swap.txt --out coPLINK-Test-Res.csv --memo ped2me_X_Swap  coPLINK --ped2bed $nor --out $1 --nor -1  coPLINK --bed2boost $1 --out $2 --nor -1  coPLINK --compare $2.txt --file2 $cmp.txt --out coPLINK-Test-Res.csv --memo ped2bed_X  coPLINK --compare $2.txt --file2 $cmp-swap.txt --out coPLINK-Test-Res.csv --memo ped2bed_X_Swap  coPLINK --ped2other p2tped.txt^#^ --file2 $nor --out $1  coPLINK --other2ped tped2p.txt^##^ --out $2  coPLINK --compare $2.ped --file2 $nor.ped --out coPLINK-Test-Res.csv --memo ped2other_X  rem --pretty2ped set the phenotypes to default 1, since Prettybase doesn't include phenotypes.  coPLINK --ped2boost $nor --out $cmp1 --nor -1 --phenotype 1  coPLINK --ped2boost $nor --out $cmp1-swap --swap --nor -1 --phenotype 1  coPLINK --ped2pretty $nor --out $1  coPLINK --pretty2ped $1.pretty --out $2  coPLINK --ped2boost $2 --out $1 --nor -1  coPLINK --compare $1.txt --file2 $cmp1.txt --out coPLINK-Test-Res.csv --memo ped2pretty_X  coPLINK --compare $1.txt --file2 $cmp1-swap.txt --out coPLINK-Test-Res.csv --memo ped2pretty_X_Swap  coPLINK --ped2svmsnp $nor --out $1 --mode 1 --nor -1  coPLINK --svmsnp2ped $1 --out $2 --mode 1 --nor -1  coPLINK --ped2ped $2 --out $1 --nor  coPLINK --ped2boost $1 --out $2 --nor -1  coPLINK --compare $2.txt --file2 $cmp.txt --out coPLINK-Test-Res.csv --memo ped2svmsnp_X  coPLINK --compare $2.txt --file2 $cmp-swap.txt --out coPLINK-Test-Res.csv --memo ped2svmsnp_X_Swap  coPLINK --ped2tped $nor --out $1 --nor -1  coPLINK --tped2ped $1 --out $2 --nor -1  coPLINK --ped2boost $2 --out $1 --nor -1  coPLINK --compare $1.txt --file2 $cmp.txt --out coPLINK-Test-Res.csv --memo ped2tped_X  coPLINK --compare $1.txt --file2 $cmp-swap.txt --out coPLINK-Test-Res.csv --memo ped2tped_X_Swap  coPLINK --ped2tped $nor --out $1 --nor -1  coPLINK --tped2bed $1 --out $2 --nor -1  coPLINK --bed2boost $2 --out $1  coPLINK --compare $1.txt --file2 $cmp.txt --out coPLINK-Test-Res.csv --memo tped2bed_X  coPLINK --compare $1.txt --file2 $cmp-swap.txt --out coPLINK-Test-Res.csv --memo tped2bed_X_Swap  coPLINK --ped2tped $nor --out $1 --nor -1  coPLINK --tped2usr-tped $1 --out $2 --nor -1  coPLINK --other2ped usrt2p.txt^###^ --out $1  coPLINK --ped2boost $1 --out $2 --nor -1  coPLINK --compare $2.txt --file2 $cmp.txt --out coPLINK-Test-Res.csv --memo tped2usr-tped_X  coPLINK --compare $2.txt --file2 $cmp-swap.txt --out coPLINK-Test-Res.csv --memo tped2usr-tped_X_Swap  coPLINK --transpose $nor.ped --out $1.txt  coPLINK --transpose $1.txt --out $2.txt  coPLINK --compare $2.txt --file2 $nor.ped --out coPLINK-Test-Res.csv --memo transpose_X  pause |
| --- |

| ^#^p2tped.txt: | ^##^tped2p.txt: | ^###^usrt2p.txt: |
| --- | --- | --- |
| [VECTOR BEGIN]  Fam,1,,,,$1.tfam  Ind,0  Pat,0  Mat,0  Sex,0  Phe,0  Chr,1,,,,$1.tped  Snp,0  Gen,0  Pos,0  [VECTOR END]  [MATRIX BEGIN]  $1.tped,5,1, ,3,2  [MATRIX END]  [INSERT BEGIN]  [INSERT END] | [VECTOR BEGIN]  Fam,1,,,,$1.tfam  Ind  Pat  Mat  Sex  Phe  Chr,1,,,,$1.tped  Snp  Gen  Pos  [VECTOR END]  [MATRIX BEGIN]  $1.tped,5,1, ,3,2  [MATRIX END] | [VECTOR BEGIN]  Fam,1,,,,$2.tfam  Ind  Pat  Mat  Sex  Phe  Chr,1,,,,$2.tped  Snp  Gen  Pos  [VECTOR END]  [MATRIX BEGIN]  $2.tped,5,1, ,3,2,,,,,,0,1,2  [MATRIX END] |

# Details of performance test

## Elapsed time and peak memory of coPLINK

Table S2 shows the results of coPLINK conversions with the data of chromosome 19 as inputs.

**Table S2** Elapsed time and peak memory of coPLINK^#^

| **Parameter** | **Elapse (s)** | **Peak memory (KB)** | **Ratio**^##^ |
| --- | --- | --- | --- |
| --pretty2ped | 5206 | 704,816 | 1.234 |
| --ped2beam | 11 | 69,560 | 0.796 |
| --beam2ped | 8 | 26,020 | 0.595 |
| --beam2boost | 5 | 25,996 | 0.594 |
| --ped2bed | 11 | 47,604 | 0.544 |
| --bed2beam | 5 | 8,400 | 1.489 |
| --bed2me | 6 | 8,448 | 1.498 |
| --bed2tped | 9 | 8,428 | 1.494 |
| --bed2boost | 6 | 31,560 | 5.596 |
| --boost2bed | 6 | 24,520 | 0.562 |
| --boost2ped | 6 | 24,520 | 0.562 |
| --space2csv | 1 | 2,056 | 0.024 |
| --csv2space | 2 | 2,004 | 0.023 |
| --ped2geo | 12 | 47,412 | 0.542 |
| --geo2ped | 11 | 26,092 | 0.597 |
| --ped2gs-linkage | 11 | 47,016 | 0.538 |
| --gs-linkage2ped | 5 | 89,788 | 2.059 |
| --ped2linkage | 15 | 47,440 | 0.543 |
| --linkage2boost | 9 | 113,064 | 1.294 |
| --linkage2ped | 6 | 90,192 | 1.032 |
| --ped2logicreg | 7 | 90,824 | 1.039 |
| --logicreg2ped | 11 | 46,716 | 0.535 |
| --ped2mdr | 11 | 69,552 | 0.795 |
| --mdr2boost | 6 | 25,376 | 0.582 |
| --mdr2ped | 7 | 25,360 | 0.582 |
| --ped2me | 12 | 46,964 | 0.537 |
| --me2ped | 12 | 46,980 | 0.539 |
| --me2boost | 9 | 46,860 | 0.537 |
| --ped2other | 16 | 48,092 | 0.550 |
| --other2ped | 24 | 140,548 | 1.607 |
| --ped2boost | 9 | 113,016 | 1.292 |
| --ped2ped | 25 | 47,524 | 0.544 |
| --ped2svmsnp | 20 | 47,444 | 0.543 |
| --svmsnp2ped | 10 | 24,660 | 0.564 |
| --ped2tped | 9 | 47,484 | 0.543 |
| --tped2ped | 7 | 181,620 | 2.077 |
| --tped2bed | 11 | 140,224 | 1.604 |
| --tped2usr-tped | 9 | 140,220 | 1.604 |
| --transpose (mode 0) | 17 | 877,784 | 10.050 |
| --transpose (mode 1) | 155 | 899,16 | 1.029 |
| --ped2pretty | 51 | 47,520 | 0.543 |

^#^Environment：Windows 7 x86 OS, Intel i3 CPU @ 3.7GHz, integrated video adapter.
^##^Ratio = Peak memory/File size

## Script of coPLINK performance test

The script was written based on Windows OS.

**List S2** Script of coPLINK performance test

| del $*.*  copy With_NBS_Ctrls_Affx_gt_CAD_Chiamo_19.* $X.*  coPLINK --ped2beam $X --out $1  coPLINK --beam2ped $1 --out $2  coPLINK --beam2boost $1 --out $2  coPLINK --ped2bed $X --out $1  coPLINK --bed2beam $1 --out $2  coPLINK --bed2me $1 --out $2  coPLINK --bed2tped $1 --out $2  coPLINK --bed2boost $1 --out $2  coPLINK --boost2bed $2 --out $1  coPLINK --boost2ped $2 --out $1  coPLINK --space2csv $X.ped --out $1.csv  coPLINK --csv2space $1.csv --out $2.txt  coPLINK --ped2geo $X --out $1  coPLINK --geo2ped $1 --out $2  coPLINK --ped2gs-linkage $X --out $1  coPLINK --gs-linkage2ped $1 --out $2  coPLINK --ped2linkage $X --out $1  coPLINK --linkage2boost $1 --out $2  coPLINK --linkage2ped $1 --out $2  coPLINK --ped2logicreg $X --out $1  coPLINK --logicreg2ped $1 --out $2  coPLINK --ped2mdr $X --out $1  coPLINK --mdr2boost $1 --out $2  coPLINK --mdr2ped $1 --out $2  coPLINK --ped2me $X --out $1  coPLINK --me2ped $1.me --out $2  coPLINK --me2boost $1.me --out $1  coPLINK --ped2other p2tped.txt --file2 $X --out $1  coPLINK --other2ped tped2p.txt --out $2  coPLINK --ped2boost $X --out $1  coPLINK --ped2ped $X --out $1 --pre  coPLINK --ped2pretty $X --out $1  coPLINK --pretty2ped $1.pretty --out $2  coPLINK --ped2svmsnp $X --out $1  coPLINK --svmsnp2ped $1 --out $2  coPLINK --ped2tped $X --out $1  coPLINK --tped2ped $1 --out $2  coPLINK --tped2bed $1 --out $2 --nor -1  coPLINK --tped2usr-tped $1 --out $2  coPLINK --transpose $X.ped --out $1.txt  pause |
| --- |
